# Wildfire Drivers, Impacts, and Predictive Modeling: Opportunities in Sensing and Machine Learning

**DOI:** 10.1029/2025GH001734

**Published:** 2026-11-11

**Authors:** Jasmin Yujen Tung, Ashok Mishra, Mojtaba Sadegh, Amitesh Sabut, Amir Aghakouchak, John Abatzoglou, Naresh Devineni, A. Sankarasubramanian

**Affiliations:** ^1^ Zachry Department of Civil and Environmental Engineering Texas A&M University College Station TX USA; ^2^ Department of Civil Engineering Boise State University Boise ID USA; ^3^ Institute for Water, Environment, and Health (UNU‐INWEH) United Nations University Richmond Hill ON Canada; ^4^ Department of Civil & Environmental Engineering University of California Irvine CA USA; ^5^ Department of Earth System Science University of California Irvine CA USA; ^6^ Department of Management of Complex Systems University of California, Merced Merced CA USA; ^7^ Department of Civil Engineering City University of New York (City College) New York NY USA; ^8^ Department of Civil, Construction, and Environmental Engineering North Carolina State University Raleigh NC USA

**Keywords:** wildfire

## Abstract

Wildfire prediction and management remain inherently challenging because fire behavior is governed by complex, nonlinear interactions among the atmosphere, biosphere, geosphere, and human influences. Intensified by climate change and anthropogenic activities, wildfires are becoming more severe in portions of the world, degrading air quality, threatening public health, generating substantial economic losses, and producing secondary hazards such as debris flows and water contamination. This review synthesizes recent advances in multi‐sensor remote sensing and machine learning approaches for wildfire management, focusing on three key domains: pre‐fire risk monitoring, active‐fire detection, and post‐fire severity assessment. It highlights how emerging sensing technologies and predictive models integrate diverse environmental variables, remotely sensed spectral indices, and geospatial data sets to improve wildfire forecasting, situational awareness, and decision‐making. Despite these advances, important limitations remain, including constrained physical interpretability and operational reliability in purely data‐driven models, limited transferability of empirical approaches across regions, and sensor‐related constraints such as sparse revisit times, radiometric inconsistencies, and atmospheric interference from clouds and smoke that affect wildfire monitoring and post‐fire assessment. Overall, the review concludes that bridging the gap between research and operational implementation will require hybrid physics‐informed modeling frameworks and multi‐platform geospatial data fusion systems capable of delivering more accurate, interpretable, scalable, and operationally robust wildfire prediction and management.

## Plain Language Summary

Wildfires are growing more severe across certain regions as a result of rising temperatures, extended droughts, and increasing human influence. Wildfires can destroy homes, and infrastructure while producing smoke that harms air quality and public health. Predicting when and where wildfires will occur, and how they will spread, remains difficult because fire behavior depends on many changing factors, including weather conditions, vegetation, terrain, and human activities. This review summarizes how advances in remote sensing technologies and artificial intelligence (AI) are improving wildfire monitoring and prediction. Satellites, drones, aircraft, and ground‐based sensors can help identify fire‐prone conditions, detect active fires, and assess damage after a wildfire. methods can analyze large and complex data sets to support faster and more accurate forecasting. However, challenges remain, including limitations in sensor observations caused by clouds and smoke, as well as the limited transparency and reliability of some AI models. The review highlights the need to combine AI with scientific knowledge of fire behavior and to integrate data from multiple sensing platforms. These improvements could provide more accurate and timely information to support wildfire preparedness, response, and recovery, helping protect communities, ecosystems, and critical infrastructure.

## Key Points

- Effective prediction requires incorporating both natural and human ignition factors, particularly in wildland–urban interface zones
- Advances in remote sensing and machine learning have enhanced wildfire detection but face challenges in data integration and accessibility
- Addressing wildfire risks needs adaptive, tech‐driven, and community‐centered strategies backed by sustained research and collaboration

## 1 Introduction

Wildfires have increasingly become a major global crisis in recent years, posing severe threats to both society and the environment, including risks to lives, property, air quality, and local economies (Cunningham et al., 2025; Teymoor Seydi et al., 2025). For example, Australia faced a devastating bushfire season in 2019–2020 that impacted nearly 3 billion animals and caused significant ecological and environmental harm (Abram et al., 2021; Australia, 2020). Wildfires in the western U.S. have been a growing problem for communities, partially fueled by intensifying drought and vegetation stress linked to climate change (Abatzoglou & Williams, 2016; Dennison et al., 2014) and a century of fire exclusion in dry forests that have increased fuel loads (Parks et al., 2025). The wildfire damages in California only in 2018 totaled $148.5 billion, about 1.5% of the state's GDP, based on models estimating capital losses, health costs from air pollution, and indirect economic disruptions (Wang et al., 2021). Economically, wildfires inflict significant damage on property and infrastructure and require costly fire suppression and recovery efforts, placing pressure on local and national budgets (Bayham et al., 2022; Modaresi Rad, Abatzoglou, Kreitler, et al., 2023). Furthermore, wildfires disproportionately affect socioeconomically vulnerable groups (e.g., older adults and people with disabilities) due to a variety of reasons including the convergence of the geography of heightened wildfire risks and vulnerable communities, limited resources and constrained access to information (Lambrou et al., 2023; Modaresi Rad, Abatzoglou, Fleishman, et al., 2023). These catastrophic footprints, the billions in economic loss and the vest ecological devastation, serve as more than just evidence of severity. They provide compelling proof of a systemic failure in current single‐domain management paradigms. This is an urgent necessity to move beyond fragmented approaches toward an integrated Decision Support System (DSS) that effectively bridges the gap between high‐precision physical forecasting and long‐term socio‐economic resilience.

While fire naturally helps maintain ecological balance, increasing wildfire intensity driven by human activities and climate extremes has disrupted this role, turning fires into a destructive force. (Riviere et al., 2022; Zhai & Woodberry, 2011). Wildfires affect areas beyond the burn zone, harming health, recreation, property, and ecosystems. They damage wildlife habitats, release harmful pollutants, and cause soil erosion that contaminates water sources. For example, following the 2017 Thomas Fire in Ventura and Santa Barbara counties, increased sediment runoff into the Ventura River carried pollutants like nitrogen, phosphorus, organic carbon, and polycyclic aromatic hydrocarbons (PAHs) (Jumps et al., 2022; Shakesby, 2011; Smith et al., 2011). Additionally, the loss of vegetation and development of water‐repellent soils after fires heighten the risk of flooding, debris flows, and mudslides, especially in steep regions (Partington et al., 2022). Wildfires release harmful pollutants such as PM2.5, PM10, ozone, carbon monoxide (CO), nitrogen dioxide (NO_2_), PAHs, and volatile organic compounds (VOCs), all of which degrade air quality and pose serious health risks. These include respiratory and cardiovascular illnesses, burns, injuries, and even death (Isaak et al., 2010; Liu et al., 2015; Reid et al., 2016).

Wildfires need two key elements: fuel and an ignition source—from either human activity or lightning (Plucinski, 2012). Abundance, continuity, type and moisture level of fuels, as well as topography and weather, shape the underlying wildfire dynamics, which in turn determine wildfire behavior and outcome. Wildfires are also facilitated by hydroclimate extremes such as droughts and heatwaves. Rising global temperatures increase wildfire risk by increasing vapor pressure deficit (VPD) and reducing fuel and soil moisture levels (Ellis et al., 2022) thereby enabling larger fires—particularly in fuel‐rich forested regions (Juang et al., 2022; Williams et al., 2019). However, in fuel‐limited fire regimes, relationships between burned area and fuel moisture are more tenuous, with surface fuel accumulation driven by sequential wet–dry extremes in preceding years showing stronger associations (Abatzoglou et al., 2018).

Effective wildfire management depends on continuous monitoring, a task that is difficult globally and particularly challenging in remote or inaccessible regions. To this end, remote sensing technologies have become essential tools in both wildfire practice and research. Satellite platforms like Landsat (USGS), MODIS‐TERRA/AQUA (NASA), and Sentinel (ESA) offer valuable long‐term data for tracking environmental conditions associated with wildfires, either pre‐ or post‐burn, including live and dead fuel conditions (e.g., (Khorshidi et al., 2020)). Landsat imagery, for example, is widely used to derive indices such as normalized difference vegetation index (NDVI) for vegetation health and normalized difference water index (NDWI) for leaf water content (Karamihalaki et al., 2016; Rouse et al., 1973). These indices support early warning systems for fire potential (Yebra et al., 2013), aid in detecting wildfire events (Kong et al., 2015) and help assess post‐fire forest recovery (Hislop et al., 2018). Recent advances in ML and deep learning (DL) techniques have significantly contributed to wildfire prediction, monitoring and recovery simulation. These models integrate both spatial and temporal data to enhance risk assessment, fire behavior modeling, and decision‐making in wildfire management (Jain et al., 2020).

This review provides an integrated synthesis of the key processes influencing wildfire occurrence, impacts, and management under changing climatic conditions, linking wildfire drivers, climate change amplification, and cascading environmental and societal impacts with emerging technologies for improved risk assessment and management (Figure 1). It develops a unified conceptual framework connecting the major components shaping wildfire risk and response. The primary drivers of wildfire occurrence are examined in Section 2, including weather related factors such as temperature, drought, wind, and humidity, fuel characteristics such as vegetation type, biomass accumulation, and fuel moisture, and human influences including land use change, fire suppression, and ignition sources in the WUI. Climate change amplification of wildfire risk is discussed in Section 3, focusing on rising temperatures, altered precipitation patterns, longer fire seasons, and more frequent extreme events. The cascading impacts of wildfires across ecosystems, soil and water systems, air quality, public health, and socioeconomic conditions are synthesized in Section 4. Emerging technological approaches for wildfire prediction are reviewed in Section 5, including ML models and subseasonal to seasonal (S2S) forecasting using statistical, dynamical, and hybrid methods. Advances in satellite remote sensing for wildfire detection, monitoring, and post fire assessment are highlighted in Section 6, supporting the tracking of fire activity, vegetation conditions, and burned areas. Together, these approaches contribute to integrated decision support systems for wildfire early warning and risk assessment. Strategies for wildfire mitigation and resilient design are outlined in Section 7, followed by key challenges and future research directions in Section 8, emphasizing improved data integration, interdisciplinary collaboration, and stakeholder informed wildfire management.

**Figure 1:**
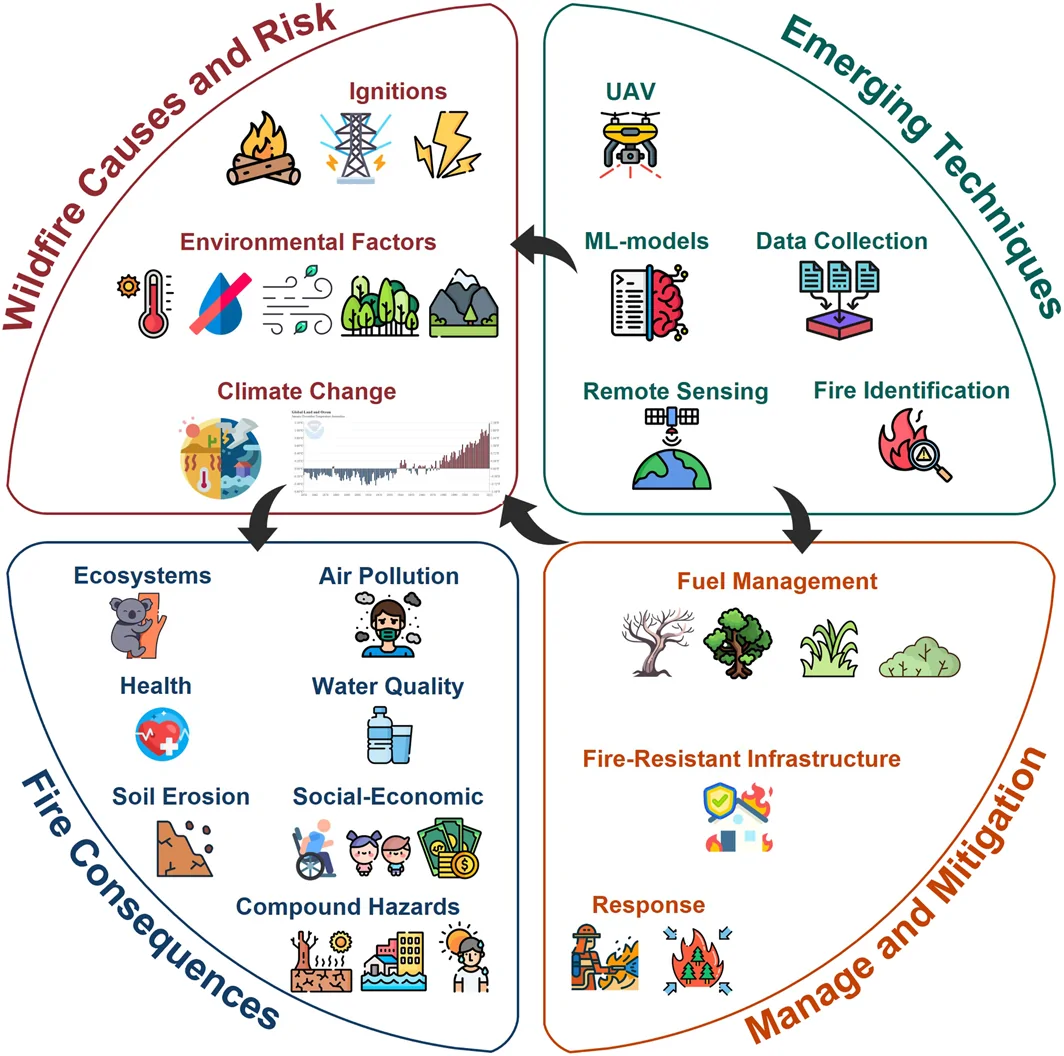
Overview of the interconnected components of wildfire science, including wildfire drivers, emerging technologies, impacts, and mitigation strategies.

## 2 Wildfire Drivers

Understanding wildfire behavior requires identifying the primary drivers—the key factors that determine when, where, and how fires ignite, develop, and spread (Benali et al., 2023). Elements such as fuel characteristics (e.g., land cover, biomass, fuel type), topography (e.g., elevation, slope, aspect), and weather conditions (e.g., temperature, humidity, wind, precipitation) collectively shape wildfire dynamics (Speer & Goodrick, 2022). These dynamics, in turn, dictate specific fire behaviors, including the rate of spread (ROS), fire growth rate, fire radiative energy (FRE), and flame length (Benali et al., 2023). Dynamic drivers can be broadly categorized into three groups: weather conditions, fuel characteristics, and human activities. Each of these plays a distinct but interconnected role in shaping the wildfire outcome. Weather drivers, such as temperature, wind, and humidity, can perturb wildfire dynamics, while drivers related to fuel, including vegetation type, moisture content, and fuel continuity determine the combustion processes and resultant fire behavior metrics, such as ROS and flame length. Factors associated with human activity, such as land use and fire suppression practices, further complicate the fire dynamics. As illustrated in Figure 2, these drivers do not act in isolation; rather, they interact dynamically within the wildfire system, collectively determining the likelihood, extent, and intensity of wildfire events.

**Figure 2:**
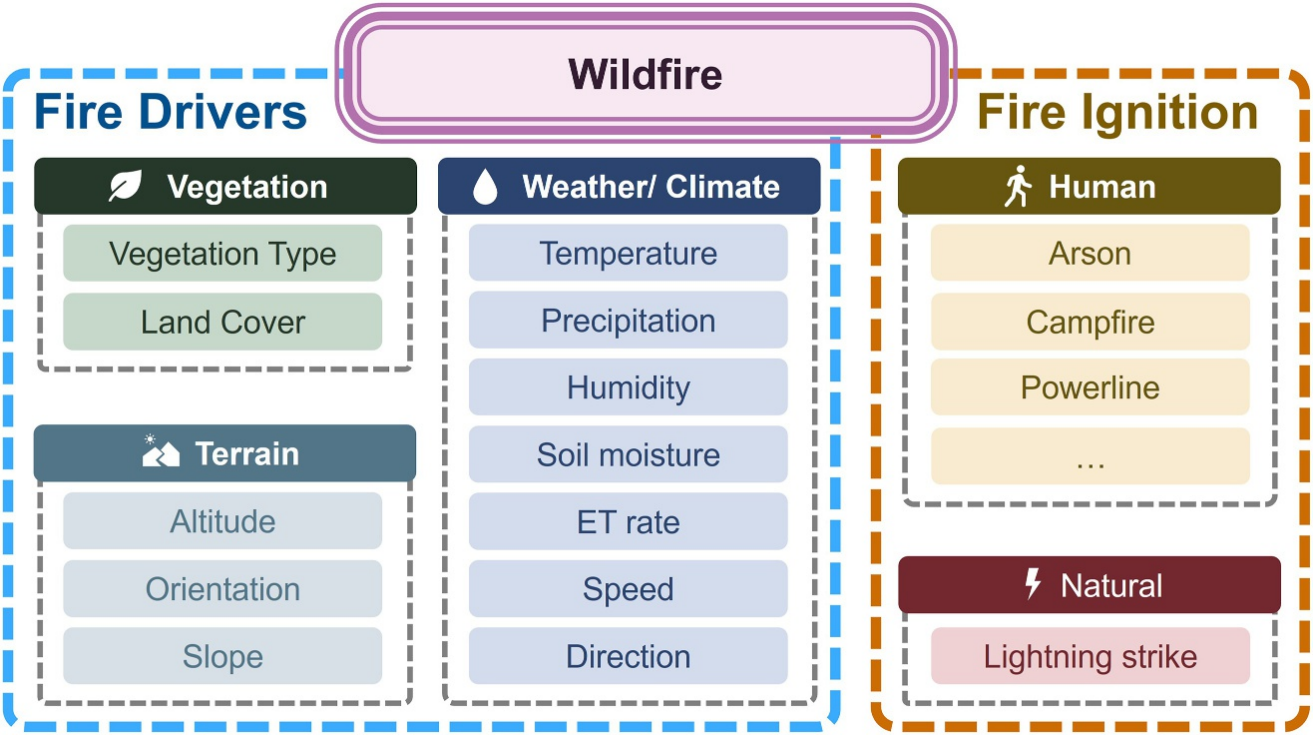
Conceptual diagram of the multi‐dimensional drivers of wildfire dynamics: weather conditions, fuel characteristics, and human activities interact across space and time to shape wildfire likelihood, behavior, and impacts..

### 2.1 Weather‐Related Factors

Regions characterized by seasonal low humidity, high temperatures, and abundant vegetation typically create favorable conditions for wildfire development (Jain et al., 2022). Relative humidity, which measures the percentage of actual water vapor pressure relative to the maximum the air can hold at a given temperature, is a key indicator of atmospheric moisture. Another important metric VPD, which quantifies the difference between actual and saturated vapor pressure. VPD serves as a reliable indicator of atmospheric dryness and the extent of water vapor deficiency in the air (Williams et al., 2019). Soil moisture also plays a critical role, as it affects plant hydration and, by extension, vegetation flammability (Holden et al., 2025). In addition, precipitation and evaporative demand influence fuel moisture and fire risk. Precipitation anomalies can be quantified using standardized indicators such as the Standard Precipitation Index (SPI), which is effective in detecting both droughts that may enable fire in forested regions and periods of unusually high rainfall that may promote fine fuel accumulation and fire potential in fuel‐limited fire regimes (Isia et al., 2022; Zubieta et al., 2023). When combined with evaporation anomalies, these indicators offer deeper insight into regional water availability and surface energy balance. A deficit in precipitation, coupled with high temperatures and VPD significantly increases wildfire risk. This combination creates conditions of elevated flammability and ignition potential, particularly during extended dry periods or drought events. This combined analysis helps assess atmospheric and soil dryness, providing a more comprehensive understanding of wildfire risk.

Precipitation conditions also affect the likelihood of ignition by lightning strikes. A crucial distinction exists between dry and wet lightning, as wildfire‐igniting events often occur under limited precipitation (e.g., Kalashnikov et al. (2023)). However, the ignition efficiency of lightning is a function of not only precipitation amount, but also vegetation and fuel moisture calling for the need for region‐specific thresholds to improve prediction accuracy and fire management strategies (Papavasileiou & Giannaros, 2024; Plucinski, 2012; Šturm et al., 2012).

Climate indices, such as the El Niño Southern Oscillation (ENSO), play a significant role in shaping regional hydrological cycles by influencing patterns of precipitation, temperature, and atmospheric circulation. Beyond immediate weather, these indices capture slowly evolving teleconnections that serve as critical precursor signals for subseasonal‐to‐seasonal (S2S) forecasting. Integrating these signals allows predictive models to extend forecasting horizons, up to six or 7 months during strong events, providing the essential lead‐time required for proactive resource allocation and strategic land management (Harrison & Meindl, 2001; Xu & Van Kooten, 2014). In addition to ENSO, other atmospheric oscillations (e.g., the Arctic Oscillation, Pacific North American pattern, Pacific Decadal Oscillation (PDO), East Pacific North Pacific pattern, and North Atlantic Oscillation) have been integrated into wildfire studies to account for their influence on regional climate variability (Ji et al., 2024; Meng et al., 2024).

### 2.2 Fuel Factors

Different types of vegetation have unique characteristics that significantly influence wildfire behavior. In semi‐arid grassland dominated regions, grasses typically grow during the wet season and cure in the dry season. Anomalous accumulation of dead fine fuels can aid in fuel continuity and fire intensity in fuel‐limited systems, resulting in increased fire potential in the following year, depending on the fuel decomposition rate. In contrast, wildfire probability in fuel‐rich forests is more directly driven by flammability facilitated by prolonged dry conditions and high VPD. This dichotomy of wildfire risk mediated through climate‐fuel relationships exists across global biomes but is also evident at finer spatial scales. For example, semi‐arid portions of southern California's is moisture‐limited, where large fire likelihood depends on fuel accumulation tied the prior year's precipitation, whereas large fire potential in wetter forested portions of the state are more directly influenced by warm and dry conditions during spring and summer that increase landscape flammability (Westerling & Bryant, 2008; Williams et al., 2019).

Vegetation exhibits substantial variability in composition, structure, and moisture, all of which influence wildfire risk, making accurate fuel assessment essential for wildfire research. Remote sensing indices such as NDVI, NDWI, Enhanced Vegetation Index (EVI), and Near‐Infrared Reflectance of Vegetation (NIRv) provide valuable information on vegetation health, but they do not always directly reflect fire susceptibility. Consequently, detailed studies that account for specific vegetation types and local conditions are critical for accurately identifying the factors driving wildfire risk and for informing effective fire management strategies. Beyond monitoring vegetation health and fuel conditions (Hernández Ayala et al., 2021), these spectral indices (e.g., NDVI, NIRv) serve as high‐resolution input features for the ML models in Section 5, providing the continuous variables necessary for the data‐driven engine to capture complex wildfire risk relationships. Historical records show that large wildfires (e.g., the 2025 Los Angeles Fires) are far more likely when fuel that accumulated during wet periods is later exposed to hot and dry conditions (AghaKouchak et al., 2025; Swain et al., 2025). Wet periods promote vegetation growth and fuel continuity, while rapid transitions to hot, dry conditions create ideal environments for extreme fire events (Abatzoglou & Kolden, 2013).

### 2.3 Human Activities

Wildfire typically starts from two primary types of ignition sources: natural and human‐related. Natural ignitions are mostly caused by lightning strikes, which mostly tend to occur in remote areas and are an integral part of many ecosystems' natural cycles. In contrast, human activities (e.g., campfires, downed power lines) have become a leading cause of wildfires in different parts of the world (Figure 3), especially near human settlement (Balch et al., 2017). Moreover, while lightning ignited fires predominantly occur during the core summer months, human‐caused fires can occur throughout the entire year where fuels are dry enough (Figure 4). Because these fires often ignite close to developed areas, they present significantly higher risks to lives, property, and infrastructure. For example, in Portugal, over 70% of ignitions occurred near municipalities with over 100 inhabitants per square kilometer, mostly within 500 m of urban areas or major roads (Catry et al., 2007; Ganteaume et al., 2013). In California, fire frequency peaks in WUI areas with intermediate population densities (35–45 people/km^2^), particularly where vegetation is sufficiently continuous to support fire spread (Syphard et al., 2007).

**Figure 3:**
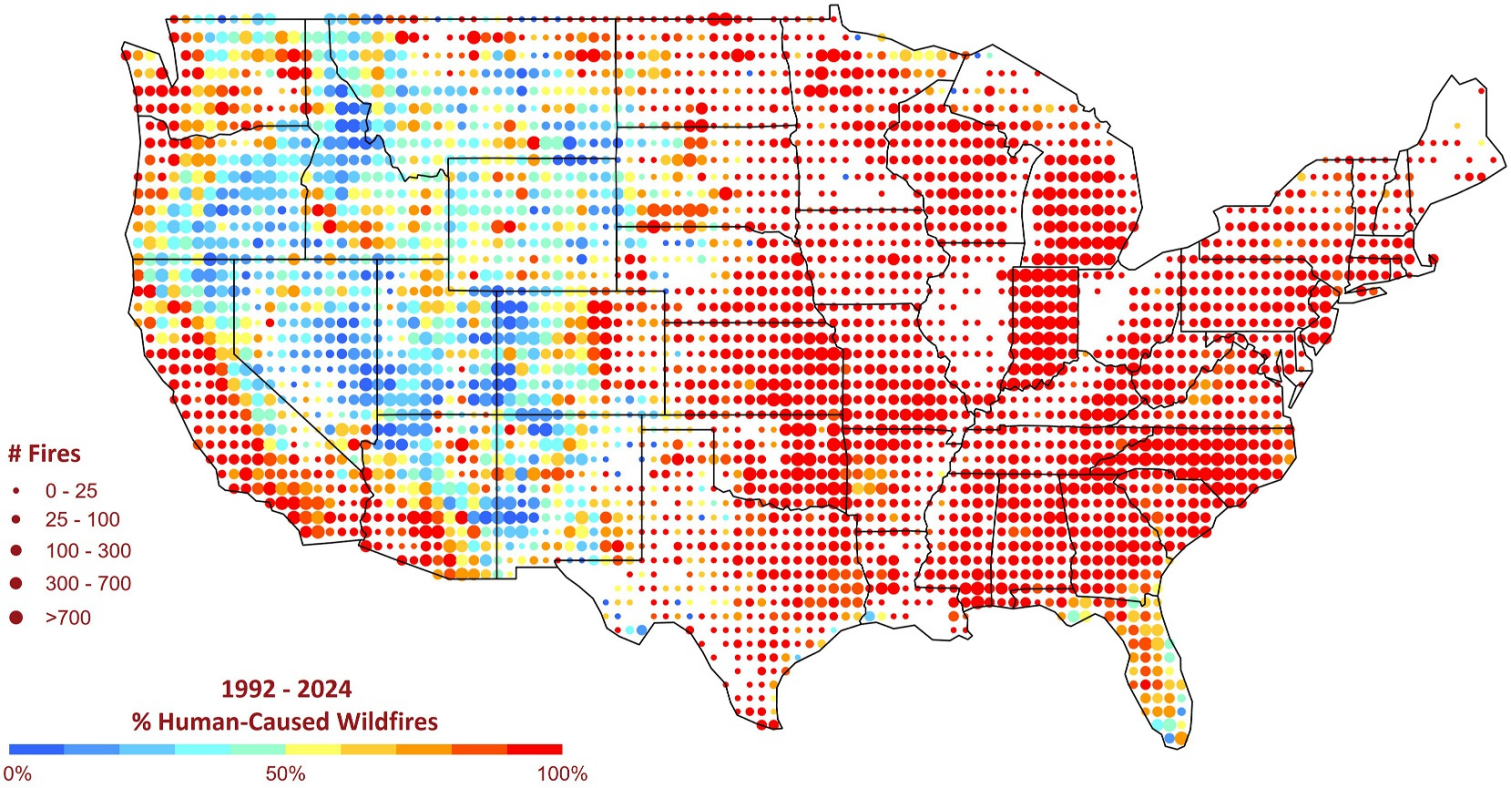
Wildfire occurrences in populated regions of the United States from 1992 to 2024, based on the National Interagency Fire Center (InFORM Fire Occurrence Data). The distribution of wildfire ignitions reflects the intetaction of human activities, natural ignition sources, and environmental conditions across the landscape. Human‐related ignitions are an important contributor in many regions, influenced by factors such as population density, land use, infrastructure, and socioeconomic conditions..

**Figure 4:**
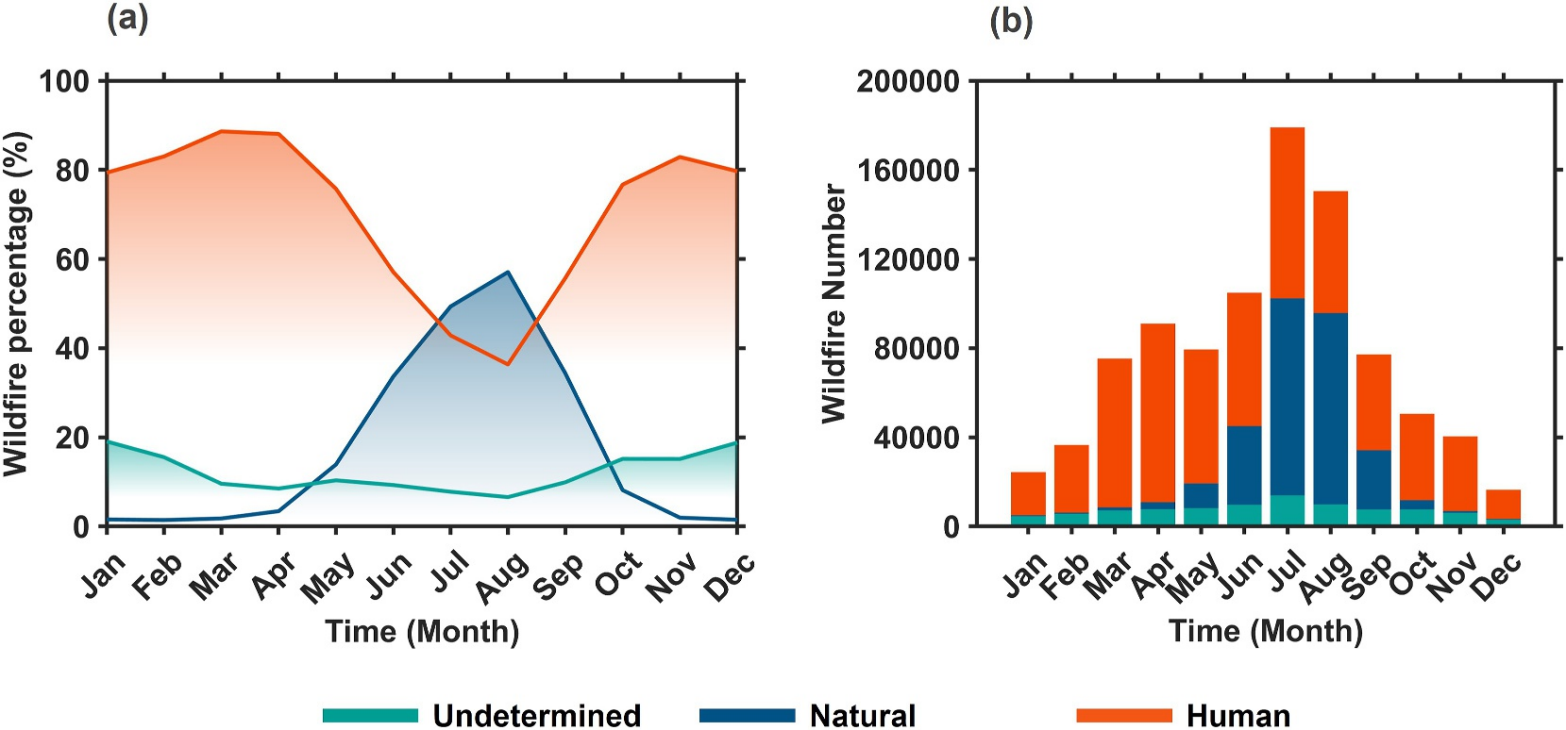
Temporal distribution of wildfire causes in the United States from 1992 to 2024, categorized into human‐caused ignitions (represented in blue), natural‐caused ignitions primarily due to lightning (represented in red), and fires with undetermined causes (represented in yellow). Panel (a) illustrates the percentage share of wildfires attributed to each cause category, while panel (b) shows the total number of wildfire incidents for each cause over the study period.

Human‐induced wildfires often lead to higher damage and greater economic cost. From 1992 to 2015, nearly one‐third of suppression costs and 97% of residential homes threatened by wildfires in the US were attributed to human‐caused incidents (Mietkiewicz et al., 2020). The WUI presents a unique challenge, where human development meets flammable vegetation. To assess the risk of human‐induced fires, studies frequently examine factors such as population density, proximity to roads, land use and land cover, and socioeconomic indicators like poverty and unemployment rates (Mercer & Prestemon, 2005). Additional considerations include public holidays and day‐of‐the‐week (Albertson et al., 2009), and powerline distribution (Malik et al., 2021). Despite the progress made in forecasting wildfires, predicting human behavior remains challenging. Consequently, much of the literature emphasizes environmental conditions conductive to wildfire ignition, aiming to mitigate risks through early intervention (Pourmohamad et al., 2025).

## 3 Climate Change and Regional Wildfire Intensification

Climate change is precipitating a qualitative shift in global fire regimes, increasingly destabilizing historical relationships between meteorological cycles and fuel availability. As escalating VPD drives unprecedented fuel desiccation, fire behavior frequently transcends historical precedents, rendering static historical indices less reliable. This transformation requires DSS to evolve from static frameworks toward the dynamic adaptation strategies powered by the advanced ML tools discussed later in this review. Specifically, rising global temperatures have contributed to more extreme weather events, such as prolonged heatwaves and persistent droughts, which in turn heighten wildfire risk in flammability‐limited areas (Senande‐Rivera et al., 2022). Figure 5 illustrates the interconnected relationship between climate change and wildfire occurrence through a conceptual diagram. Climate change has lengthened wildfire seasons by extending dry periods including that tied to reduced snowpack leaves soils and vegetation drier for longer (Boegelsack et al., 2018; Jones et al., 2024). Rising temperatures, particularly during heatwaves, increase both the likelihood and intensity of wildfires by enhancing ignition efficiency and promoting more severe burns (Jones et al., 2022). Fire intensity is further amplified by the interaction between strong winds and increased fuel aridity; even if such winds become less frequent, their occurrence under warmer and drier conditions can substantially escalate wildfire severity (Hawkins et al., 2022; Williams et al., 2019). These climatic shifts are expanding wildfire activity into previously low risk regions characterized by humid or cool climates, thereby increasing the frequency and geographic extent of fires and contributing to more widespread and severe wildfire threats globally (Abram et al., 2021; Burke et al., 2021; Jones et al., 2022).

**Figure 5:**
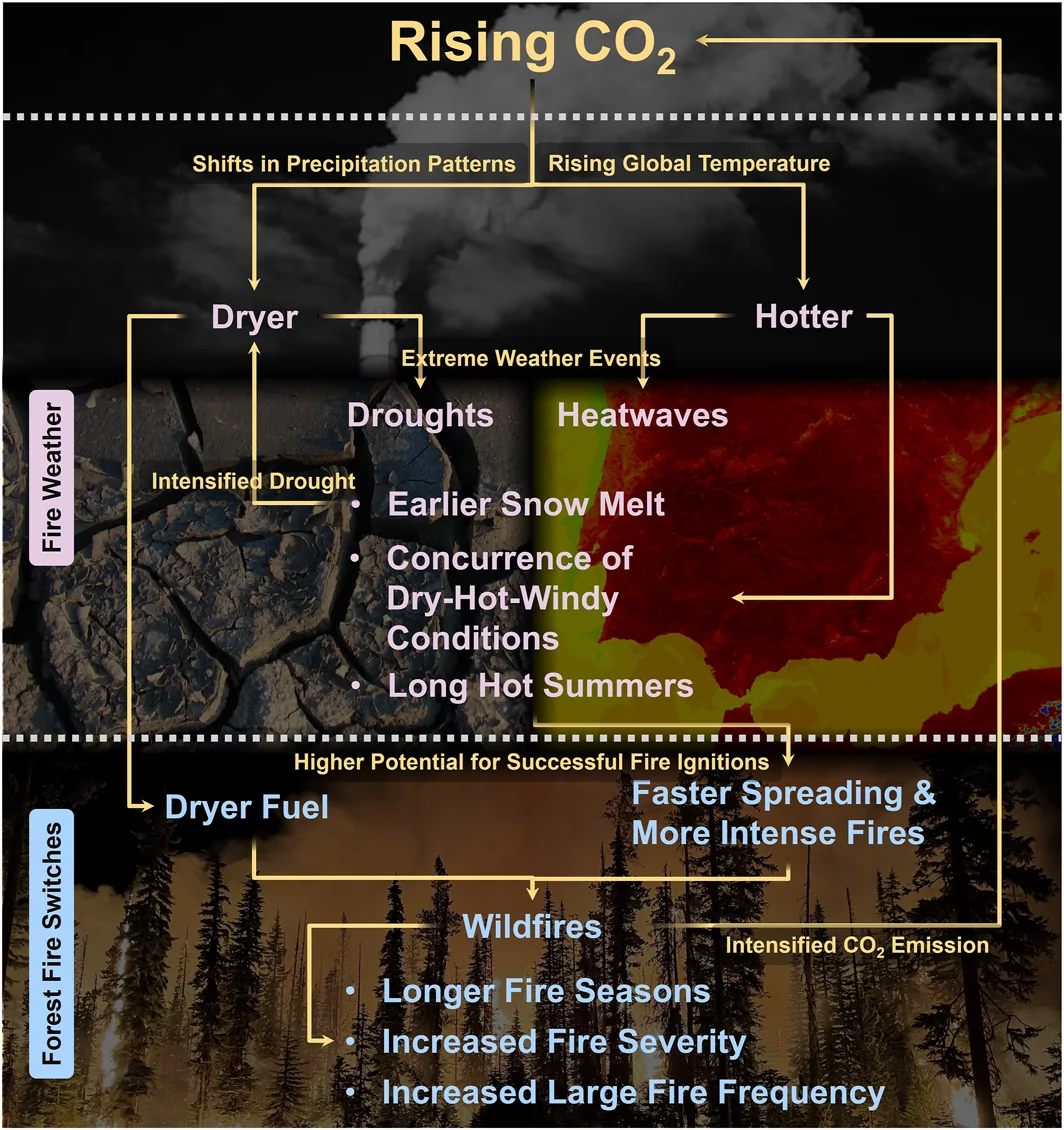
Conceptual diagram of the interconnected relationship between climate change and wildfire occurrence. Rising temperatures and shifting weather patterns are increasing wildfire frequency, intensity, and geographic spread, even in regions previously considered low risk.

Figure 6 presents major events worldwide between 2019 and 2025, highlighting how drought, extreme heat, and strong wind have collectively driven some of the most destructive fire seasons on record. Global wildfire trends indicate that the total area burned may be stable or declining, the frequency and intensity of the most extreme wildfire events (the top 0.01% in energy) have increased sharply by 2.2‐fold over the past two decades, particularly worsening in boreal and temperate conifer regions (Cunningham et al., 2024). In Australia, Canadell et al. (2021) reported a significant increase in forest area burned over a 32‐year period, marked by a steady annual rise and a sharp escalation in fire activity during autumn and winter months. These trends are closely linked to worsening fire weather conditions caused by climate change, with megafire years becoming more frequent since 2000. Similarly, in the Eastern Temperate Forests of the United States, wildfire activity has increased significantly over the past 36 years. Large fires have become more common, more intense, and have affected larger areas (Donovan et al., 2023).

**Figure 6:**
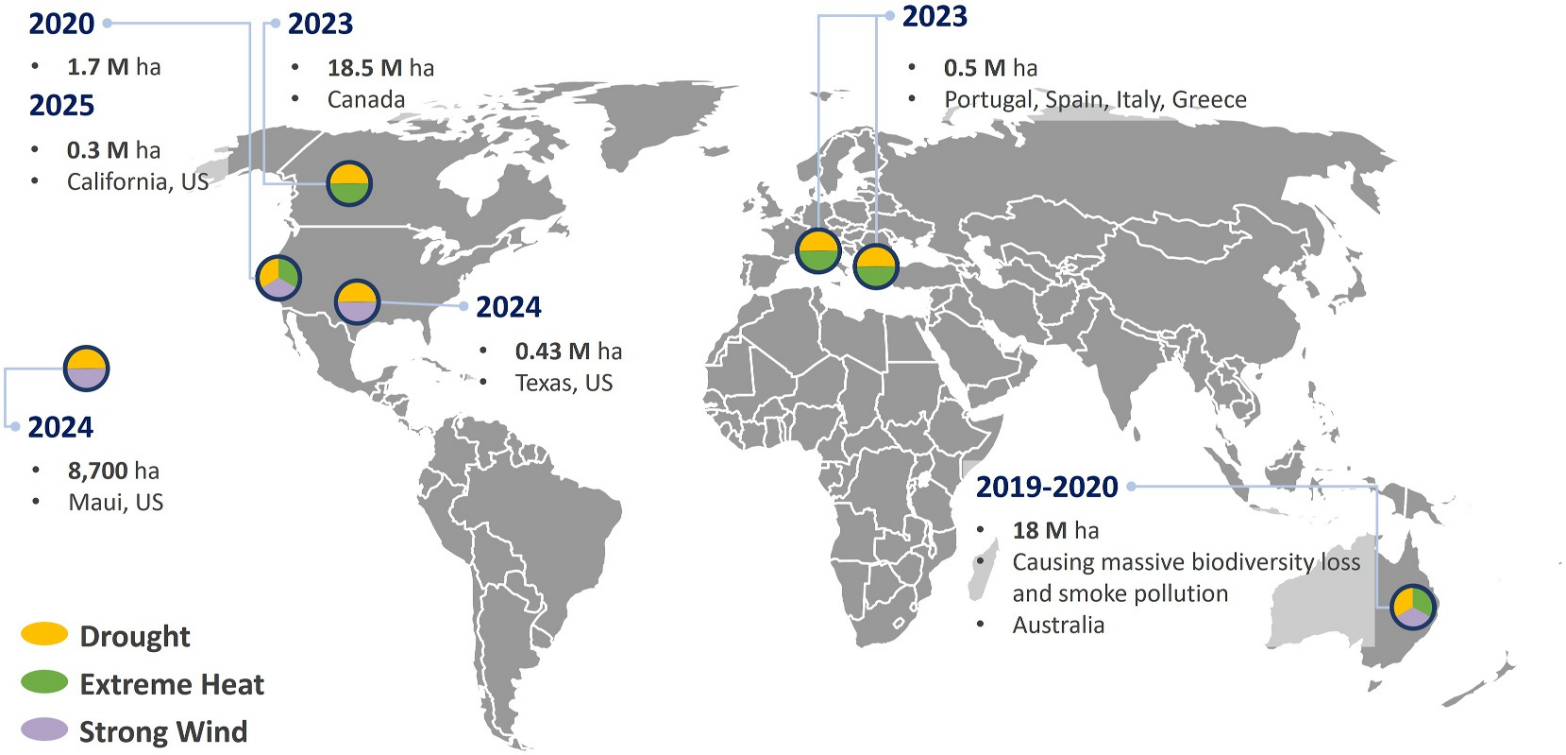
Global distribution of major wildfire events (2019–2025).

In February 2024, Northern Texas experienced an uncharacteristic winter wildfire event. According to the Texas House of Representatives (2024) report, the Smokehouse Creek Fire burned more than 1,058,000 acres, making it the largest wildfire in Texas history. This extraordinary event was driven by a combination of factors, including unseasonably warm temperatures exceeding 80°F (26.7°C) and strong winds over 50 mph, which created ideal conditions for rapid fire spread. Additionally, above‐average rainfall during the previous spring led to increased grass growth, which later dried out and served as abundant fuel. Rising temperatures accelerate vegetation drying between rainfall events, extending wildfire seasons and increasing the likelihood and severity of fires (Texas House of Representatives, 2024).

In the summer of 2023, Southern Europe, particularly Greece and Spain, was devastated by catastrophic wildfires fueled by record‐breaking heatwaves and prolonged drought. Greece experienced its largest wildfire on record, burning over 140,000 ha. These fires caused widespread environmental destruction and loss of life. The severity and frequency of such events are strongly tied to the intensifying impact of climate change on regional weather patterns (Dosiou et al., 2024; Koundouri, 2023). That same year, in August 2023, the Hawaiian island of Maui suffered destructive wildfires, especially in the historic town of Lahaina. Invasive grasses, such as Guinea grass, provided highly flammable fuel. A detailed methodological analysis indicates that the behavior of the Maui wildfire was primarily driven by strong downslope wind dynamics rather than by anomalous antecedent dryness (Mass & Ovens, 2024).

Climate change has significantly altered wildfire patterns, intensifying compound hazards (AghaKouchak et al., 2020; Zscheischler et al., 2018) by combining extreme weather events like droughts and heatwaves with wildfires. These conditions not only increase wildfire severity but also lead to secondary hazards such as air pollution, flooding, and water contamination (Littell et al., 2016). Figure 7 illustrates these interconnected risks, highlighting how climate‐driven extremes create an environment highly conducive to more frequent and intense wildfires. As climate change continues to alter global weather patterns, predicting wildfire behavior becomes more challenging. Traditional models that rely on stable seasonal patterns must be updated to include the growing frequency of extreme weather events and anomalies. Effective wildfire forecasting will require integrating both long‐term climate trends and the increasing likelihood of unusual weather conditions.

**Figure 7:**
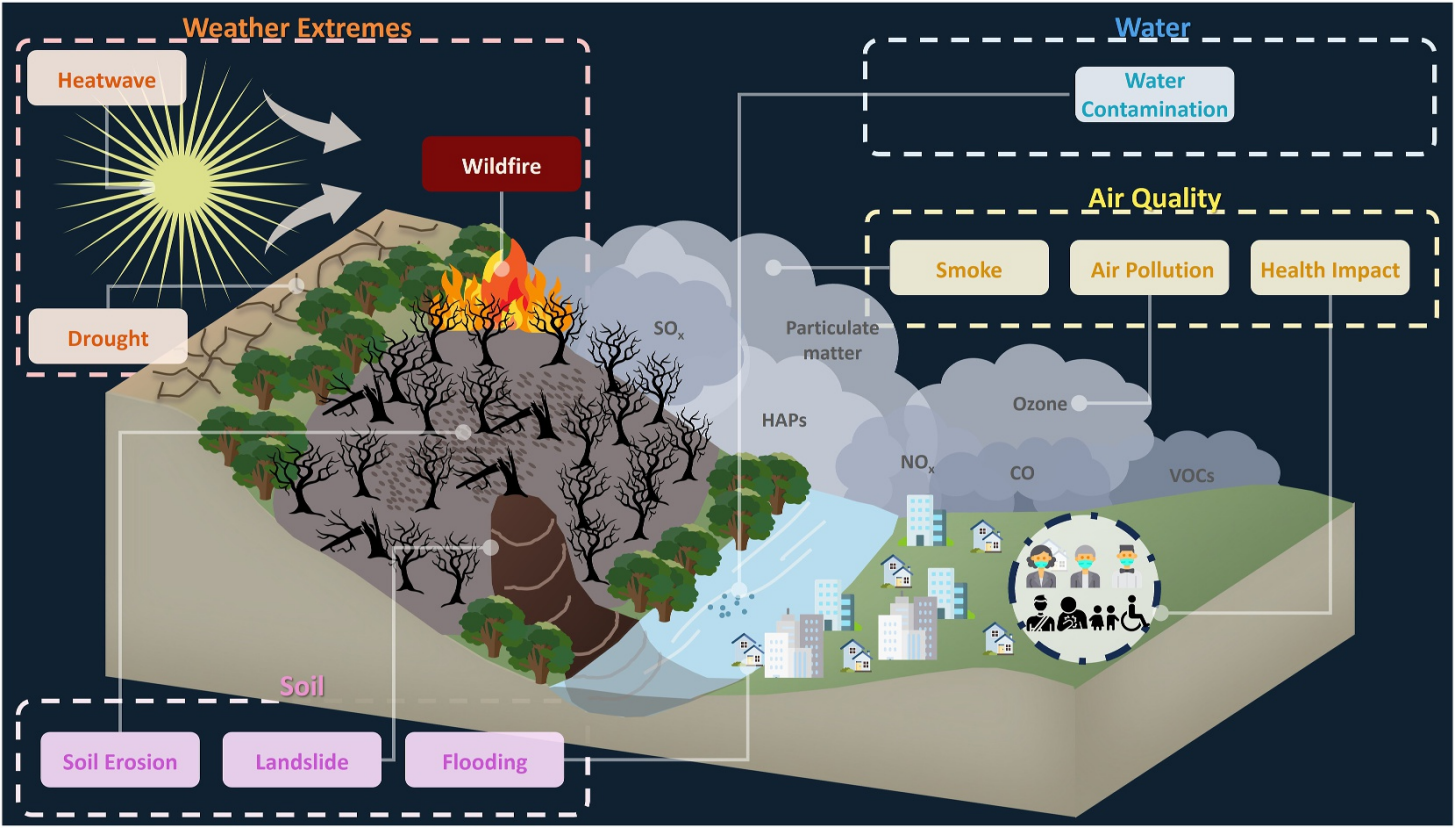
Conceptual diagram of interconnected and overlapping hazards induced by wildfire activity. These compound hazards may include secondary effects such as post‐fire flooding, landslides, degraded air quality, and long‐term ecosystem disruption. The diagram highlights how wildfire triggers a cascade of related events that collectively amplify risks to human health, safety, and the environment.

## 4 Consequences of Wildfires

The following sections analyze the multi‐dimensional consequences of wildfires, which serve as the primary objective functions for the DSS framework established in this review (see Figure 11). While traditional models often prioritize forecasting ignition probability, it is critical to recognize that burned area alone does not accurately represent risk. Instead, true wildfire risk is defined by the intersection of fire dynamics and specific vulnerability indicators, ranging from socio‐economic disparities to infrastructure resilience, at the impact site. Wildfires, while natural in many ecosystems and sometimes beneficial for ecological renewal, can become destructive when they occur too frequently or burn too intensely. In fire‐adapted regions, occasional fires help maintain healthy landscapes, but excessive wildfires can disrupt biodiversity, destroy habitats, and hinder the recovery of plant and animal species (Jones et al., 2022; Vidal‐Riveros et al., 2023). Burned areas are also more vulnerable to soil erosion and increased runoff, which can pollute water sources and increase the risk of flash flooding (Shakesby & Doerr, 2006; Smith et al., 2011; Williams et al., 2014). In addition, wildfires release large amounts of air pollutants (e.g., carbon dioxide and fine Particulate matter (PM)) that affect air quality across wide regions (Jaffe et al., 2020; Liu et al., 2015).

The health and social impacts of wildfires are equally precarious. Smoke exposure can lead to respiratory and cardiovascular issues, especially for vulnerable populations like children, the elderly, and individuals with pre‐existing health conditions (Liu et al., 2015). Mental health effects, injuries, and stress from evacuations or property loss are also common. On a broader scale, wildfires bring significant economic costs, from firefighting operations and infrastructure damage to long‐term displacement and loss of income. These burdens are often very high in rural and low‐income communities, where recovery resources may be limited (Rad et al., 2023). As illustrated in Figure 8, wildfire impacts ripple across environmental, health, and socio‐economic dimensions, weaving a complex web of consequences that can persist long after the flames are extinguished. By categorizing these impacts, DSS can shift from simple event detection to sophisticated risk prioritization. The following section offers an overview of the societal impacts of wildfire.

**Figure 8:**
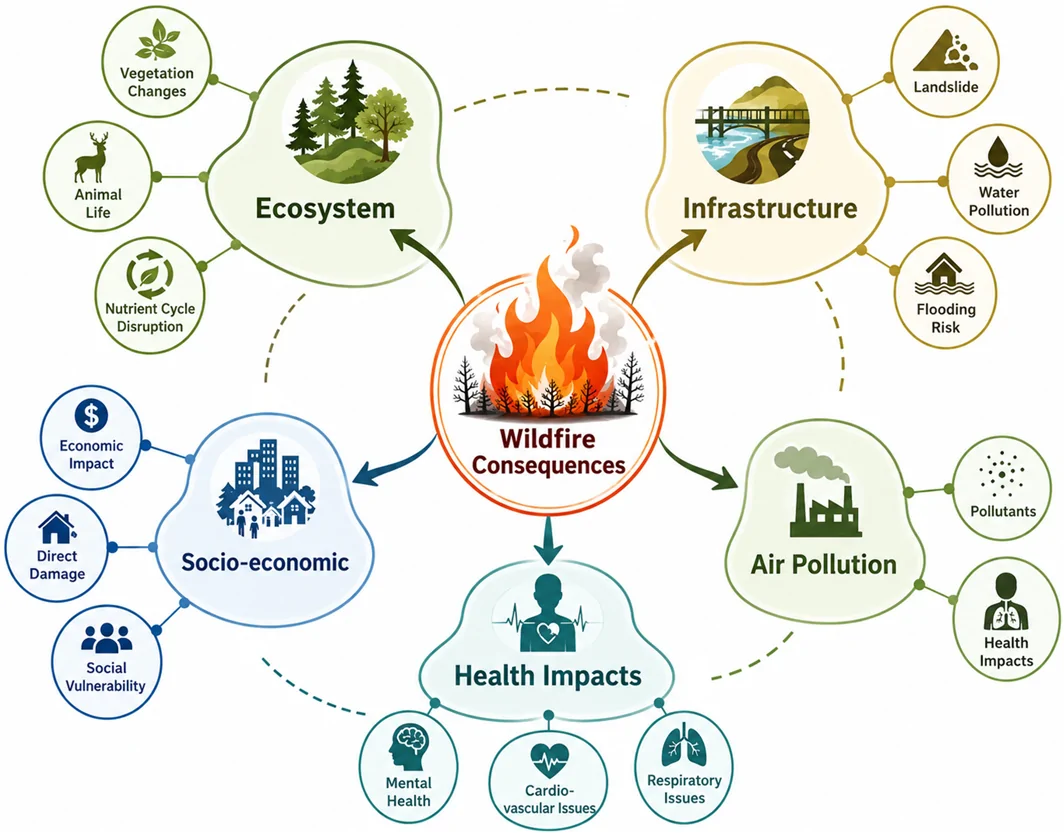
Consequences of wildfire on socioeconomic systems, ecosystems, human health, and infrastructure.

### 4.1 Impacts of Wildfires on Biodiversity and Ecosystem

Wildfires have profound and often lasting impacts on ecosystems, particularly vegetation type conversion and biodiversity (Lindenmayer et al., 2022). Intense fires can destroy plant cover, alter species composition, and encourage the spread of invasive species. Soil seed banks may be damaged, natural regeneration disrupted, and biodiversity significantly reduced (Kasel et al., 2024). In the Gran Chaco region, for instance, wildfires deplete essential soil nutrients, diminish water retention capacity, and shift ecological balance in favor of invasive species, threatening both grasslands and forests (Vidal‐Riveros et al., 2023). Wildlife is also severely affected, with many animals unable to escape fast‐moving flames, resulting in population declines and habitat fragmentation (Albery et al., 2021; Ayars et al., 2023). These changes not only reduce species numbers but also disrupt critical ecological interactions, such as mutualisms and commensal relationships that underpin ecosystem function. However, under natural fire regimes, periodic burning can also provide ecological benefits by maintaining habit diversity and supporting fire‐adapted species (Pausas & Keeley, 2019). Charles et al. (2025) examine 52 specialized plant‐animal interactions in fire‐prone ecosystems, highlighting how shifts in fire regimes can disrupt these relationships and lead to significant eco‐evolutionary impacts. Their findings suggest that while natural fire patterns help sustain and stabilize these partnerships, sudden changes in fire frequency or intensity pose a serious risk of collapse. A prominent example is the 2019/2020 Australian bushfires, which impacted over 30% of habitats for 70 vertebrate species, including 21 that were already classified as endangered (Jones et al., 2022).

Beyond immediate damage, wildfire disrupts key ecological processes like the carbon and nitrogen cycles (Dove et al., 2022; Granath et al., 2020; Pellegrini et al., 2020). They emit an estimated 8 billion tons of CO_2_ annually, and although some of this carbon is reabsorbed during vegetation regrowth, frequent and severe fires often lead to a net increase in atmospheric carbon (Jones et al., 2022). While many ecosystems have evolved to cope with occasional fires, current changes in fire regimes driven largely by climate change exceed natural thresholds, threatening ecosystem resilience and the continuity of vital services such as pollination and seed dispersal (Souto‐Veiga et al., 2022). Mitigating these ecological impacts requires urgent climate action and sustainable land management practices that reduce both the frequency and intensity of wildfires.

### 4.2 Soil Degradation and Water Quality Risks After Wildfires

Wildfires have significant and wide‐ranging effects on soil stability, water quality, and hydrological processes. By removing vegetation, altering soil structure, and depleting organic matter, fires leave soil exposed and highly susceptible to erosion. These changes disrupt natural water cycles and increase the risk of runoff and sediment transport (Sanchez‐Garcia et al., 2023; Stavi, 2019). Aquatic ecosystems also suffer as post‐fire runoff carries ash, sediment, and debris into rivers and lakes, reducing oxygen levels, increasing turbidity, and harming aquatic life (Murphy et al., 2023). Vegetation acts as a natural barrier against wind and water erosion, but once it is removed by fire, the soil becomes vulnerable to displacement. Burned soil frequently develops water‐repellent layers that inhibit water infiltration, intensifying surface runoff and reducing groundwater recharge. High‐intensity fires worsen this degradation by destroying organic matter essential for soil cohesion and nutrient cycling. Post‐wildfire hydrological responses vary across watersheds, with peak flows influenced not only by rainfall intensity and duration (Brogan et al., 2017), but also by antecedent groundwater storage and evaporative demand, which affect baseflow and water availability (Bart & Tague, 2017).

The degree of fire impact depends on fire severity, soil type, and topography. Soil affected by fire often shows increased water repellency and reduced aggregate stability, both of which raise the risk of erosion. For instance, raindrops striking bare soil surfaces can dislodge particles, while runoff accelerates sediment transport. These erosion processes are most intense within the first year after a fire but tend to decline as vegetation gradually recovers, particularly in fast‐regenerating landscapes like grasslands and shrublands (Certini, 2005; Mataix‐Solera et al., 2011; Shakesby & Doerr, 2006).

Post‐fire erosion also introduces sediments, ash, and excess nutrients into water bodies, leading to significant water quality degradation (Nitzsche et al., 2024). High concentrations of suspended solids increase turbidity, while runoff rich with nitrogen and phosphorus can trigger eutrophication, resulting in algal blooms and oxygen‐depleted zones that endanger aquatic life. Moreover, wildfire ash often contains organic carbon, salts, and toxic substances such as heavy metals, which further alter water chemistry (Cerrato et al., 2016). Runoff from burned areas can shift water pH, reduce dissolved oxygen levels, and introduce persistent organic pollutants like PAHs, posing threats to both ecosystems and human health (Rao & Parsai, 2023). Sediment accumulation in reservoirs also reduces water storage capacity, impairs water management, and heightens flood risks (Certini, 2005; Mishra et al., 2021; Smith et al., 2011).

The loss of vegetation and the development of hydrophobic soil layers after a wildfire dramatically increase the risk of flooding and debris flows (Movasat & Tomac, 2024; Samburova et al., 2023). With diminished capacity to absorb rainfall, fire‐affected landscapes generate rapid surface runoff during storms. According to Psomiadis et al. (2020), runoff on burned slopes may increase by 1.2%–14.7%, while the potential for erosion also rises significantly. This heightened runoff often leads to flash floods, landslides, and debris flows, particularly in steep terrain (Partington et al., 2022). Debris flows are fast‐moving mixtures of water, soil, rocks, ash, and vegetation that rush downhill following heavy rain on fire damaged land with water repellent soils. These hazards can persist for years after a wildfire but generally decline as vegetation re‐establishes and stabilizes the soil (Williams et al., 2014).

Regions such as the western United States vividly demonstrate the link between increasing wildfire frequency, climate change, and rising hydrological hazards. Post‐fire flooding in these areas has repeatedly caused widespread damage to infrastructure, ecosystems, and human settlements (Sankey et al., 2024). A single intense storm over burned rangeland in Colorado, for example, caused severe flooding, significant property damage, and major transportation disruptions (Williams et al., 2014). Additionally, wildfire ash, made up of both organic and mineral materials, plays a crucial role in shaping post‐fire environmental impacts. Its composition varies depending on burn severity and ecosystem type, but it often contributes to soil instability, water contamination, and air quality problems. When dispersed by wind or carried into water bodies, ash can increase sedimentation, contaminate drinking water sources, and pose respiratory risks to nearby communities (Sanchez‐Garcia et al., 2023).

### 4.3 Air Quality and Health Impacts of Wildfire Smoke

Wildfire releases a wide array of harmful air pollutants that affect both nearby and distant communities. These pollutants originate not only from burning vegetation but also from the combustion of human‐made structures, which emit additional toxic chemicals. Wildfire smoke contains fine particulate matter (PM_2.5_), CO, ozone, and various other hazardous air pollutants (HAPs), all of which contribute to respiratory and cardiovascular illnesses, worsen chronic conditions, and increase mortality rates, particularly among vulnerable populations (Jacob & Winner, 2009; Jaffe et al., 2020; McCracken et al., 2024).

Particulate matter (PM), especially PM_2.5_, is a major component of wildfire smoke and poses significant health risks because it can penetrate deeply into the lungs and enter the bloodstream, leading to severe respiratory and cardiovascular problems (Heaney et al., 2022). During wildfire events, PM_2.5_ concentrations often spike, frequently surpassing health safety limits. This fine PM causes oxidative stress, inflammation, and damage to the airway, worsening chronic respiratory conditions like asthma and COPD, and resulting in increased emergency department visits. Prolonged exposure further elevates the risk of respiratory infections, presenting serious public health concerns (Liu et al., 2015; Reid et al., 2016). Ozone (O_3_), while not directly emitted, forms through chemical reactions involving nitrogen oxides and VOCs released during fires (Lee et al., 2021). Elevated O_3_ levels exacerbate respiratory conditions such as asthma and reduce lung function in exposed individuals (Nuvolone et al., 2018; Uysal & Schapira, 2003). CO, produced by incomplete combustion, is another significant pollutant that impairs oxygen transport in the bloodstream, posing severe health risks (Ernst & Zibrak, 1998).

In addition to these pollutants, wildfires emit HAPs including acetaldehyde, acrolein, chloroform, formaldehyde, manganese, and tetrachloroethylene, many of which are carcinogenic or cause serious health problems (O’Dell et al., 2020; Rice et al., 2023). PAHs and sulfur oxides are also released during combustion, further degrading air quality and posing public health risks. A major concern is the ability of wildfire smoke to travel great distances; smoke plumes carried by atmospheric circulation can transport pollutants thousands of kilometers from the fire source, affecting air quality and health in far‐flung regions (Roiger et al., 2012). For example, smoke from wildfires in the western United States has been detected as far away as the eastern coast of the United States and even Europe, amplifying the environmental and health impacts of these fires (Jacob & Winner, 2009).

Exposure to wildfire smoke elevates the risk of cardiovascular morbidity and mortality, especially in individuals with pre‐existing heart diseases. The fine particles induce systemic inflammation and vascular stress, aggravating hypertension, arrhythmia, and heart failure (Liu et al., 2015; Reid et al., 2016). Wildfires also impact other health outcomes, including adverse birth effects such as reduced birth weight, systemic inflammation, bone marrow suppression, diabetes, and gastrointestinal illnesses. Physical injuries from direct wildfire contact further reduce health and recovery capacity (Liu et al., 2015). Notably, long‐term exposure to wildfire‐related PM_2.5_ is linked to a 5% increased risk of dementia per interquartile range increase (HR 1.05; 95% CI 1.02–1.08), even after adjusting for other pollutants (B. Zhang et al., 2023).

Mental health consequences of wildfire smoke are significant yet often overlooked. Survivors commonly suffer from major depressive disorder, generalized anxiety disorder, and post‐traumatic stress disorder. These effects can persist long‐term, impacting quality of life and community cohesion. Additionally, wildfire smoke exposure is linked to increased suicide rates in rural areas, especially among vulnerable groups (Molitor et al., 2023). Beyond public health, wildfire smoke significantly disrupts air transportation. Dense smoke reduces visibility, creating safety hazards for flights, and in severe cases, leading to airport closures that cause economic losses and logistical challenges. Furthermore, PM in the smoke can damage aircraft engines and critical components, increasing maintenance costs and operational risks (Gössling et al., 2023).

### 4.4 Socioeconomic Consequences of Wildfires: Economic Burdens and Community Vulnerabilities

Wildfires impose profound socioeconomic consequences, disrupting communities on multiple levels. Beyond their damage to ecosystems, geology, and human health, wildfires destabilize livelihoods, strain public and private resources, and deepen existing social inequalities (Paudel, 2023; Thomas et al., 2022). These impacts are far‐reaching and multifaceted, with long‐term effects on both urban and rural regions. Infrastructures such as homes, businesses, power lines, and transportation systems are frequently destroyed or rendered inoperable by wildfires, halting economic activity and daily life. Recovery efforts, including firefighting and rebuilding, further strain budgets. Tourism also suffers due to environmental degradation and perceived safety risks (Bayham et al., 2022). Indirect costs, though less visible, often exceed direct damages and may persist for years. For instance, in the case of the Schultz Fire, which occurred northeast of Flagstaff, Arizona, United States, in June 2010, approximately 88% of total costs were indirect. Largely due to post‐wildfire flooding and mitigation efforts, these expenses include flood control infrastructure, uninsured household damages, and the values of lost wildlife habitat (Colavito et al., 2021). Additionally, indirect impacts such as the loss of ecosystem services and community well‐being accumulate over time. In 2020 alone, wildfires caused an estimated $16.5 billion in direct damages across the United States (Bayham et al., 2022; Smith & Katz, 2013). Short‐term disruptions include a spike in demand for emergency personnel, while sectors like agriculture, forestry, and tourism often suffer operational setbacks and layoffs. Poor air quality caused by wildfire smoke reduces labor productivity, estimated to result in a $93 billion annual loss in labor earnings across the United States (Bayham et al., 2022; Borgschulte et al., 2024). Property values typically decline after wildfires, though this effect may fade over time (Conlisk et al., 2024; Xu & van Kooten, 2013). In addition, wildfire risk influences local economic viability: mandatory disclosure of wildfire hazard zones in California reduces home prices by an average of 4.3%, with stronger effects following major fire events, reflecting buyer awareness of heightened risk, potential insurance costs, and anticipated future damages (Ma et al., 2024). Escalating insurance costs in high‐risk areas further strain communities, sometimes forcing businesses to relocate or adapt their operations toward wildfire‐related services, reshaping local economies (Bayham et al., 2022; Gourevitch & Kousky, 2025).

Wildfires place a disproportionate burden on socioeconomically vulnerable populations, who are more likely to reside in remote or underserved areas with elevated fire risk. Limited financial resources, language barriers, and restricted access to timely information reduce their ability to prepare for, respond to, and recover from wildfire events, reinforcing long‐standing cycles of vulnerability. On the U.S. West Coast, for example, the number of highly vulnerable individuals exposed to wildfires increased by nearly 250% between 2000–2010 and 2011–2021. Those most at risk include older adults, people with disabilities, residents in group housing, and individuals with limited English proficiency, all of whom face heightened challenges in evacuation and access to safe temporary shelter. In California's 2018 Camp Fire, for example, 80% of the fatalities were among older adults (Modaresi Rad, Abatzoglou, Fleishman, et al., 2023). In addition to geographic and social disparities, biological and demographic factors significantly influence vulnerability to wildfire health impacts. Ethnic minorities, individuals with genetic predispositions, young children, older adults, and those with pre‐existing health conditions like asthma or cardiovascular disease face heightened risks due to weaker immune or respiratory systems. Low socioeconomic status and limited healthcare access further exacerbate these health outcomes (Kochi et al., 2010; Liu et al., 2015).

## 5 Wildfire Forecasting Using Machine Learning Models

Wildfire predictive modeling has increasingly benefited from advances in ML and DL, particularly for forecasting fire occurrence, mapping fire susceptibility, estimating fire spread, and assessing post‐fire impacts. Recent research highlights the growing role of these data‐driven approaches in hydroclimatic extreme forecasting and risk assessment, where complex interactions among climate, vegetation, terrain, and human activities must be captured effectively (Tripathy & Mishra, 2024). As illustrated in Figure 9, ML applications in wildfire management span the full fire lifecycle, including pre‐fire susceptibility mapping and fire occurrence forecasting, active‐fire detection and spread modeling, and post‐fire impact assessment through burned area estimation, burn severity classification, and carbon emission quantification.

**Figure 9:**
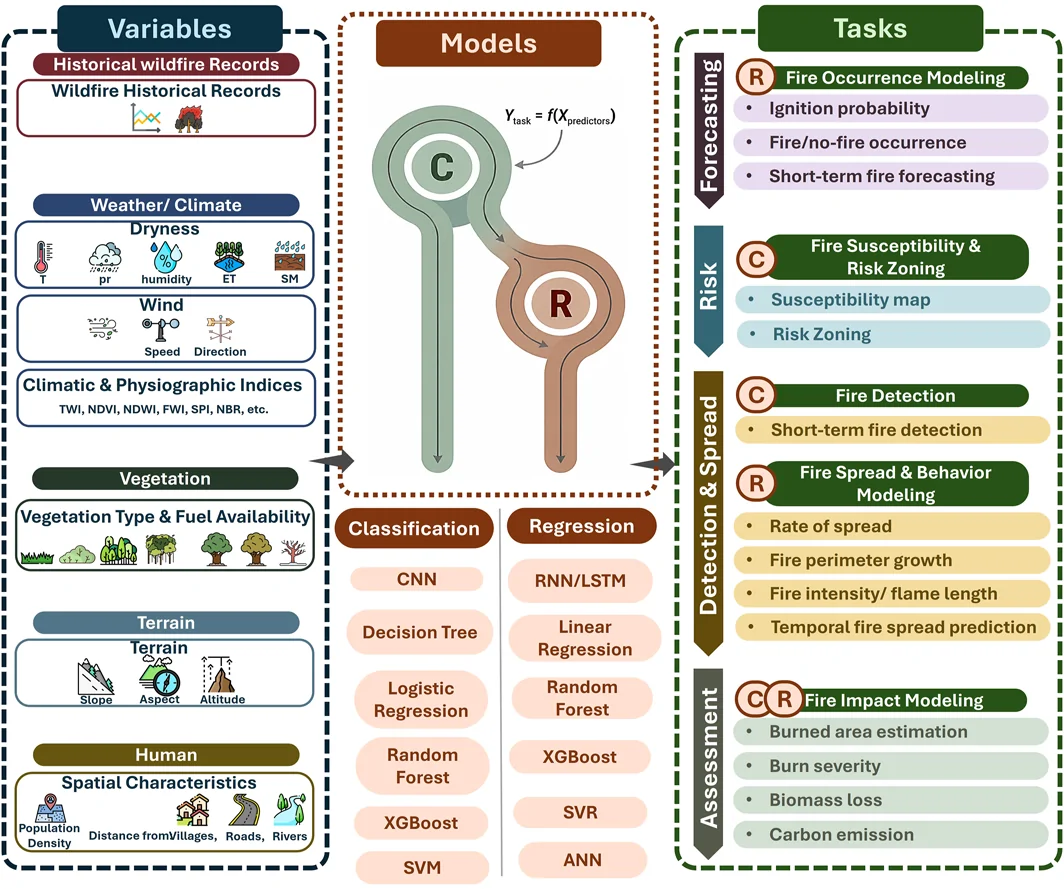
Schematic overview of machine learning tasks and modeling frameworks in wildfire research. The diagram depicts key input features including historical fire records, dryness indicators, fuel availability, terrain, wind, spatial features, and environmental indices used by ML applications. These models support diverse wildfire‐related tasks, such as fire occurrence forecasting, susceptibility and risk mapping, active fire detection, fire spread modeling, and post‐fire impact (See Table S3 in Supporting Information S1 for corresponding case studies and references).

Methodologically, wildfire prediction tasks can be broadly grouped into classification and regression problems. Classification‐based applications include burned area mapping, and categorical burn severity assessment, whereas regression‐based applications focus on predicting continuous outcomes such as ROS, fire intensity, biomass loss, and carbon emissions. Although wildfire modeling approaches have evolved from traditional ensemble methods, such as Random Forest and eXtreme Gradient Boosting (XGBoost), to more advanced DL architectures, including Convolutional Neural Networks (CNNs), Recurrent Neural Networks, and transformers, detailed technical descriptions and case studies are provided in Text S1 in Supporting Information S1.

The predictive performance of wildfire ML models depends strongly on the integration of diverse environmental, climatic, vegetation, terrain, and anthropogenic variables. Based on a systematic analysis of 30 representative wildfire modeling studies published between 2020 and 2025, Figure 10 summarizes the most commonly incorporated input variables across the fire management spectrum (Table S1 and Table S2 in Supporting Information S1 (Bürger et al., 2020; Casallas et al., 2022; Cetin et al., 2023; Coban & ERDİN, 2020; Çolak & Sunar, 2020; Dhakal et al., 2024; Feizizadeh et al., 2023; Fernández‐Manso et al., 2024; Heo et al., 2023; Juvanhol et al., 2021; Kumari & Pandey, 2020; Maniatis et al., 2022; Michael et al., 2021; Mohammed et al., 2022; Novo et al., 2020; Parajuli et al., 2020; Peng et al., 2024; Pourghasemi et al., 2020; Santos et al., 2022; Sapkota et al., 2025; Shafapourtehrany, 2023; Shi & Zhang, 2023; Sivrikaya & Demirel, 2025; Sivrikaya et al., 2024; Tan & Feng, 2023; C. Wang et al., 2025; Xu et al., 2023; Zhang et al., 2020; Zhao et al., 2021; Ziccardi et al., 2020)).

**Figure 10:**
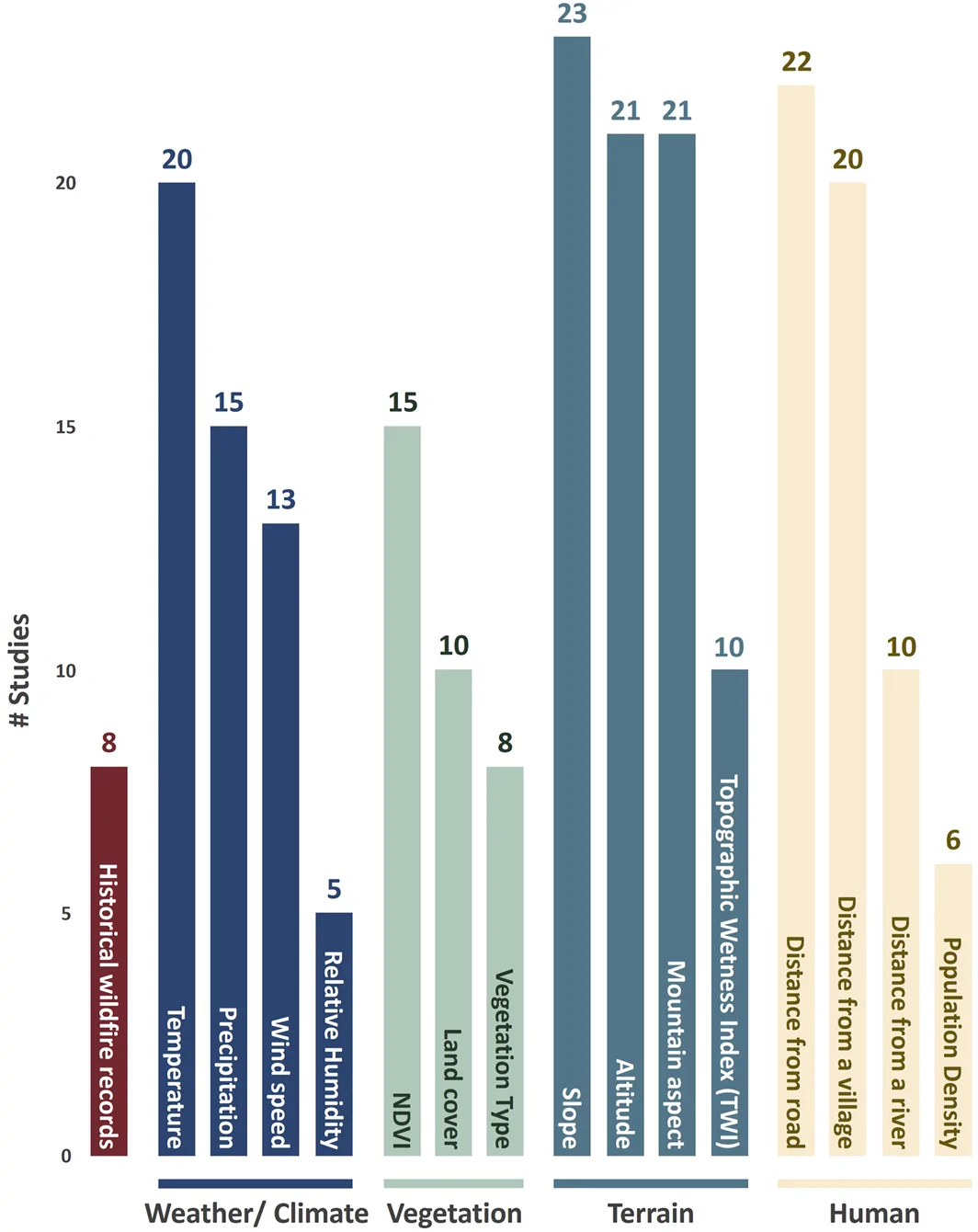
Comparison of the most commonly used drivers in wildfire forecasting models. This analysis is based on 30 representative wildfire risk modeling studies published between 2020 and 2025, sourced from the Web of Science database, highlighting the key roles and applications of these factors.

Weather and climate variables, including temperature, humidity, precipitation, and wind, are among the most widely used predictors because they directly influence fuel dryness, ignition probability, and fire spread potential. Vegetation characteristics, such as vegetation type, density, and moisture content, together with terrain features such as slope, aspect, and elevation, provide critical information on fuel availability and landscape controls on fire behavior. Derived indices, including NDVI, Normalized Burn Ratio (NBR), Fire Weather Index (FWI), and SPI, further synthesize vegetation condition, burn severity, fire‐weather risk, and drought stress into model‐ready inputs. Human‐related variables, particularly distance to roads, proximity to settlements, and population density, capture the strong anthropogenic influence on ignition patterns and spatial fire risk. The following subsections describe how these variables are integrated within classification and regression frameworks.

### 5.1 Wildfire Occurrence Prediction

Wildfire occurrence prediction focuses on estimating the likelihood, timing, and spatial distribution of future fire events using historical and near‐real‐time environmental, climatic, vegetation, terrain, and anthropogenic information. Accurate wildfire prediction is critical for protecting ecosystems, reducing economic losses, and supporting timely disaster preparedness, evacuation planning, and fire‐management strategies (Ali et al., 2024; Bao et al., 2024).

Early wildfire occurrence prediction was mainly based on statistical, stochastic, and expert‐based probabilistic models. These approaches treated fire occurrence as a probabilistic process influenced by fuel moisture, fire‐weather indices, and seasonal conditions. Cunningham and Martell (1973) used a Poisson‐based framework to model human‐caused fire occurrence, while Cunningham and Martell (1976) incorporated subjective probability assessments from experienced fire managers. Other early studies evaluated the predictive value of fire‐danger rating systems and climate anomalies, linking fire activity to indices such as fuel moisture, fire spread potential, and broader climate variability (Haines et al., 1983; Simard et al., 1985).

Regression‐based models later became central to wildfire occurrence prediction, providing interpretable estimates of fire probability. Logistic regression, Poisson regression, and structured additive models were used to relate fire occurrence to fire‐weather indices, seasonality, vegetation, terrain, and human‐accessibility variables. Martell et al. (1989) incorporated seasonal variation into logistic regression, while Mandallaz and Ye (1997) showed that fire‐danger indices became more useful when combined with additional explanatory variables. Spatial logistic‐regression approaches were later applied to human‐caused wildfire risk, where variables related to settlements, roads, land use, and WUI conditions became important predictors (Ríos‐Pena et al., 2017; Vilar del Hoyo et al., 2011).

Applications of regression‐based occurrence prediction across different regions have shown that wildfire drivers vary with ecological and social context. In protected areas, vegetation indices, temperature, elevation, rainfall, and settlement proximity often explain spatial differences in fire occurrence, whereas in more populated regions, road access, agricultural activity, and built‐up areas can become more influential (Nhongo et al., 2019; Peng et al., 2023). These studies demonstrate that regression models are useful not only for prediction but also for identifying dominant regional fire drivers. However, their capacity to represent nonlinear interactions among climate, vegetation, terrain, and human activity is limited compared with more flexible machine‐learning approaches.

Machine‐learning methods were introduced to overcome some of these limitations. Random Forest, Boosting Regression Trees, Support Vector Machine (SVM), XGBoost, and Artificial Neural Networks (ANNs) have been widely tested against traditional statistical models. Comparative studies generally show that ensemble tree‐based models, especially Random Forest and boosting methods, often outperform logistic regression because they can capture nonlinear relationships and interactions among predictors (Guo et al., 2016; Rodrigues & De la Riva, 2014). ANNs have also been used for wildfire occurrence and vulnerability prediction, but their advantage over simpler statistical models is not always large (de Bem et al., 2018). Overall, machine‐learning models improved wildfire occurrence prediction by better representing complex fire–environment relationships.

Recent occurrence‐prediction studies increasingly use ensemble learning, remote sensing, and human‐activity variables. Random Forest and XGBoost models have been applied to fire‐prone regions using climate, vegetation, terrain, and land‐use variables, while oversampling and imbalance‐handling techniques have been used to improve detection of rare fire events (Caiado & Marques, 2025; Moghim & Mehrabi, 2024). Human‐related factors have also become a major focus because many wildfires are associated with land use, road access, agricultural activity, settlements, and recreation. Adding anthropogenic variables to meteorological, topographic, and vegetation predictors improves model performance and helps identify ignition‐prone areas more effectively (Jeon et al., 2025; Lee & Park, 2026).

At larger spatial scales, wildfire occurrence prediction has shifted toward large fire inventories, improved sample design, and regional transferability. Large‐scale data sets make it possible to model daily fire probability across broad domains, but they also introduce challenges related to non‐fire sample selection, spatial autocorrelation, and regional heterogeneity. Recent studies have shown that stratified fire‐absence sampling and region‐specific calibration can improve wildfire prediction, especially when environmental controls differ across space (Illarionova et al., 2025; Pourmohamad et al., 2026). These findings indicate that model performance depends not only on algorithm choice but also on how fire and non‐fire samples are defined and how well the model accounts for regional differences in climate, fuels, and human activity.

Bayesian and probabilistic models provide another important direction because they support uncertainty‐aware wildfire prediction. Instead of only producing deterministic fire/no‐fire outputs, these models estimate probability distributions and can incorporate prior knowledge, expert information, or hierarchical spatial‐temporal structure (Charizanos & Demirhan, 2023; Pimont et al., 2021). Such models are particularly useful for decision‐making because they provide both prediction and uncertainty information, which is important when allocating firefighting resources or planning early‐warning systems.

Deep‐learning approaches represent the most recent stage of wildfire occurrence prediction. These methods can integrate large, multimodal data sets. Deep neural networks, convolutional models, Long Short‐Term Memory (LSTM)‐based architectures, and multimodal frameworks have been applied to wildfire occurrence, cause, size, and short‐term likelihood prediction (Bhowmik et al., 2023; P. Hu et al., 2024; Jiang et al., 2024). Their main strength is the ability to learn complex spatial and temporal patterns from large data sets. However, DL also requires careful validation, substantial training data, and interpretability tools to avoid black‐box predictions that are difficult for fire managers to trust.

Despite the significant progress, key challenges remain, including class imbalance, absence‐sample selection, regional transferability, uncertainty quantification, and model explainability. Future wildfire occurrence prediction systems should therefore integrate high‐resolution remote sensing, fire‐weather data, vegetation stress indicators, human‐activity variables, robust sampling strategies, uncertainty‐aware modeling, and explainable artificial intelligence (AI) to improve both predictive accuracy and operational usefulness.

### 5.2 Susceptibility Mapping and Risk Assessment

Wildfire susceptibility mapping is a key component of proactive wildfire management because it identifies areas where fire is more likely to occur. Unlike real‐time fire‐weather forecasting, which focuses on short‐term fire probability, susceptibility mapping emphasizes relatively stable or slowly varying factors such as vegetation type, fuel condition, terrain, drought exposure, land cover, and proximity to roads or settlements. These maps are useful for prioritizing fuel‐reduction treatments, locating firebreaks, planning prescribed burning, guiding patrol routes, managing the WUI, and supporting long‐term land‐use and conservation planning.

Wildfire susceptibility mapping approaches have gradually evolved from knowledge‐based methods to statistical, machine‐learning, deep‐learning, and hybrid frameworks. Early studies often relied on expert‐driven multi‐criteria decision‐making approaches such as Analytic Hierarchy Process, fuzzy‐AHP, weighted overlay, and frequency ratio because detailed fire inventories and high‐resolution geospatial data sets were not always available. These methods are transparent and easy to implement, making them useful in data‐scarce regions. However, their performance depends strongly on expert judgment, factor selection, and weighting schemes. Comparative studies have shown that fuzzy logic and frequency‐ratio methods can improve upon purely rule‐based approaches by better representing uncertainty and the spatial association between fire occurrence and conditioning factors (Arca et al., 2020; Pourghasemi et al., 2016; Sinha et al., 2023).

Statistical and ecological suitability models provided an important transition toward data‐driven susceptibility mapping. Logistic regression, MaxEnt, and structured regression models allow fire occurrence to be related to environmental predictors in a probabilistic and interpretable way. These methods are useful when the goal is to understand the influence of fire‐conditioning factors. For example, MaxEnt has been used with MODIS fire records to identify broad fire‐prone environments, while logistic regression has been used to compare data‐driven susceptibility maps with expert‐based risk‐zoning methods (Renard et al., 2012; Yathish et al., 2019). Although statistical models may not fully capture complex nonlinear interactions, they remain valuable as interpretable baselines for evaluating more advanced machine‐learning models.

Conventional machine‐learning models have become widely used in recent years because wildfire susceptibility is controlled by nonlinear interactions among environmental and human drivers. Random Forest, SVM, Decision Tree, ANN, and related classifiers have been applied across various landscapes. Among these, Random Forest has often performed strongly because it can handle nonlinear relationships, mixed predictor types, and variable interactions (Gholamnia et al., 2020; Ghorbanzadeh, Valizadeh Kamran, et al., 2019; Tonini et al., 2020). In general, ensemble tree‐based models are well‐suited for mapping fire‐prone landscapes where terrain, vegetation, climate, and human‐accessibility variables interact in complex ways.

Boosting and ensemble learning represent a further advance in data‐driven susceptibility modeling. Models such as Gradient Boosted Decision Trees, LogitBoost, XGBoost, and LightGBM improve susceptibility classification performance by combining weak learners, each sequentially correcting the errors of its predecessors. These methods often outperform single classifiers and conventional regression methods (Sachdeva et al., 2018; Sun et al., 2022; Tehrany et al., 2019).

Recent studies increasingly emphasize that susceptibility mapping accuracy depends on how fire and non‐fire samples are generated and how predictors are constructed. Because wildfire occurrence is spatially imbalanced, non‐fire sample selection can strongly influence model outcomes. Resampling, bootstrap validation, spatial cross‐validation, and constrained background sampling have therefore become important components of model development (Kalantar et al., 2020; L. Zhao et al., 2024). In addition, dynamic predictors such as antecedent dryness, vegetation stress, drought conditions, and multi‐temporal remote‐sensing indices can better represent fire‐prone fuel conditions than static variables alone.

Hybrid and optimized models have also become common in wildfire susceptibility studies. These approaches combine machine‐learning or neuro‐fuzzy models with optimization algorithms such as the Genetic Algorithm and Particle Swarm Optimization. The purpose of these hybrid models is to improve parameter tuning, reduce overfitting, and increase generalization. Adaptive neuro‐fuzzy inference system, Support Vector Regression, Random Forest, and CNN models optimized with metaheuristic algorithms have shown improved susceptibility prediction compared with their standalone versions (Jaafari & Pourghasemi, 2019; Mabdeh et al., 2022; Nur et al., 2022; Razavi‐Termeh et al., 2020). These methods are especially useful in regions where fire occurrence is controlled by nonlinear relationships and where model calibration is challenging. However, they also require careful validation because highly optimized models may appear accurate but may not always transfer well to new regions or time periods.

Deep‐learning approaches have expanded wildfire susceptibility mapping by allowing models to learn spatial patterns and high‐dimensional feature interactions. CNN‐based models are useful when neighborhood context, raster predictors, or spatial patterns are important, while deeper neural architectures can incorporate complex environmental inputs. Some studies have reported strong performance from CNN or hybrid CNN‐optimization models, especially when remote sensing and spatial predictors are available (Nur et al., 2022; Rihan et al., 2023; Zhang et al., 2019). However, recent comparative studies show that DL does not automatically outperform tree‐based ML. In some cases, Random Forest, XGBoost, or stacked ensemble models provide similar or better performance with lower computational cost and easier interpretation (Biswas et al., 2025; Shome et al., 2025). Therefore, DL should be selected when its spatial‐learning capability is necessary and supported by sufficient data, rather than used only because it is more complex.

Explainable AI is becoming increasingly important because susceptibility maps must be understandable to land managers and decision‐makers. Traditional machine‐learning studies often focused on accuracy but provided limited explanation of why specific areas were classified as high risk. Recent studies have used SHapley Additive exPlanations (SHAP) and related tools to identify the relative contribution of variables such as vegetation type, NDVI, slope, land surface temperature, rainfall, humidity, road distance, and settlement proximity (Abdollahi & Pradhan, 2023; Liao et al., 2025; Uthappa et al., 2025). This is an important methodological advance because it links model outputs to recognizable physical and human drivers. Explainability also improves trust in susceptibility maps and helps translate model predictions into practical fire‐management actions.

Recent research has also moved beyond susceptibility mapping toward risk and exposure assessment. Susceptibility maps identify where fires are more likely, but risk assessment requires understanding what communities, infrastructure, ecosystems, or future development may be exposed in those fire‐prone areas. Studies integrating susceptibility with social vulnerability, infrastructure exposure, or urban‐growth scenarios provide a more decision‐relevant view of wildfire risk (Ghorbanzadeh, Blaschke, et al., 2019; Yılmaz & Alphan, 2026). This shift is important because the consequences of wildfire depend not only on where fire is likely to occur, but also on the spatial overlap between fire‐prone landscapes and vulnerable human or ecological assets (Modaresi Rad, Abatzoglou, Fleishman et al., 2023; Modaresi Rad, Abatzoglou, Kreitler et al., 2023).

Overall, wildfire susceptibility mapping has progressed from expert‐based decision models and frequency‐ratio approaches to statistical, machine‐learning, deep‐learning, hybrid optimization, and explainable AI frameworks. Knowledge‐based models are useful for data‐limited regions, while statistical models provide interpretable baselines. Machine‐learning and boosting models generally improve predictive performance by capturing interactions among terrain, vegetation, climate, and human factors. Hybrid and deep‐learning methods can further enhance modeling when sufficient data and careful validation are available, while explainable AI improves the practical value of susceptibility maps. Future studies should prioritize robust sampling, spatially independent validation, uncertainty analysis, interpretable modeling, multi‐sensor remote sensing, and integration with exposure and vulnerability layers so that susceptibility maps are not only accurate but also useful for fire prevention, land‐use planning, and climate‐adaptation strategies.

### 5.3 Active Fire Detection and Wildfire Spread Forecasting

Active‐fire detection refers to the identification and geolocation of ongoing wildfire events using satellite observations, aerial imagery, Unmanned Aerial Vehicle (UAV) platforms, and ground‐based monitoring systems, whereas wildfire spread forecasting aims to predict the future spatial and temporal evolution of fires under changing environmental conditions. Together, these processes are essential for operational wildfire management because they support rapid emergency response, evacuation planning, suppression resource allocation, and infrastructure protection. Accurate and timely detection can reduce asset damage and economic losses by enabling earlier intervention, while spread forecasting provides critical foresight for anticipating fire behavior, identifying threatened communities, and prioritizing response strategies. These technologies therefore provide the real‐time monitoring and prediction capabilities needed for effective wildfire emergency management (Saranya et al., 2025).

Traditional wildfire detection and spread forecasting approaches primarily relied on threshold‐based remote‐sensing methods, empirical fire‐spread equations, and statistical fire‐risk models. Early spread models focused on estimating fire ROS from fuel, wind, and terrain conditions, while later statistical approaches linked fire size and intensity to a broader set of meteorological drivers. Beer (1991) discussed deterministic and statistical approaches for bushfire rate‐of‐spread forecasting and highlighted that single empirical models could produce biased estimates under different fuel and environmental conditions. Alexander and Cruz (2006) evaluated active crown‐fire rate‐of‐spread predictions using wildfire observations, demonstrating the operational value of empirical spread models while also emphasizing their uncertainty. Similarly, Preisler and Westerling (2007) developed a probabilistic framework for monthly large‐fire forecasting in the western United States, while Hernandez et al. (2015) linked wildfire size and fire radiative power (FRP) to meteorological conditions in the Mediterranean Basin. These studies provided the foundation for later data‐driven fire‐behavior forecasting by framing fire spread and intensity as predictable outcomes of weather, fuel, and landscape conditions.

With the expansion of Earth observation, satellite‐based active‐fire detection became a major operational tool. Polar‐orbiting sensors provided broad spatial coverage, while geostationary satellites enabled high‐frequency fire monitoring. Xu and Zhong (2017) demonstrated the usefulness of Himawari‐8 geostationary imagery for real‐time wildfire detection and tracking in Australia, showing that high temporal resolution can support monitoring of fire‐spread direction and abnormal fire behavior. Kang et al. (2022) further used Himawari‐8 observations with machine‐learning models to reduce detection latency, highlighting the advantage of geostationary satellites for early warning. Hong et al. (2023) improved this direction through SBT‐FireNet, which integrates spatial, spectral‐band, and time‐series features from Himawari‐8 imagery to overcome the limited spatial resolution of geostationary observations. Together, these studies show that geostationary satellite systems are valuable for active‐fire monitoring because they provide frequent temporal updates, even though their relatively coarse spatial resolution requires improved detection algorithms.

Deep‐learning‐based active‐fire detection has increasingly replaced traditional threshold‐based approaches because it can learn complex spectral, spatial, and contextual patterns directly from imagery. Landsat‐based studies have been particularly important for pixel‐level active‐fire detection. de Almeida Pereira et al. (2021) developed a large‐scale Landsat‐8 active‐fire data set and showed that CNN‐based segmentation could approximate and improve upon handcrafted active‐fire algorithms. Rostami et al. (2022) advanced this direction using MultiScale‐Net, which combines multiple convolution kernel sizes, dilated convolutions, and active‐fire spectral indices to improve detection of small and isolated fire pixels. Gao et al. (2025) further addressed false alarms and interference pixels in Landsat‐8 active‐fire detection by developing discerning active fire detection model, a CNN‐based model that incorporates land surface temperature and improved feature processing. These studies indicate that DL can improve active‐fire mapping by combining spectral fire information with spatial context and multiscale feature extraction.

Multisensor fusion has also become important for active‐fire and burned‐area detection because different sensors capture complementary fire characteristics. Optical data provide detailed spectral and spatial information, thermal bands capture heat signals, and radar can provide observations under cloud or smoke conditions. Rashkovetsky et al. (2021) used a U‐Net‐based semantic segmentation framework to combine Sentinel‐1, Sentinel‐2, Sentinel‐3, and MODIS data for fire‐affected area detection. Their results showed that optical–thermal fusion was useful under clear‐sky conditions, while radar–optical fusion improved performance under cloudy conditions. Such studies demonstrate that active‐fire detection can benefit from combining visible, infrared, thermal, and microwave observations, especially when smoke, cloud cover, or atmospheric variability reduces the reliability of single‐sensor approaches.

UAV and low‐altitude remote sensing have enabled more detailed active‐fire monitoring and small‐fire segmentation. UAV imagery can provide very‐high‐resolution observations that are useful for detecting small flames, fire fronts, and localized burning areas. Seydi et al. (2022) proposed Fire‐Net, which combines RGB and thermal imagery for active forest‐fire and burning‐biomass detection. Tong et al. (2024) developed FireFormer, a lightweight CNN–Transformer model for real‐time fire segmentation from low‐altitude imagery. X. Hu et al. (2024) addressed the severe class‐imbalance problem in UAV fire segmentation using AF‐Net, which improves object‐contextual representations for small active‐fire pixels. These studies show that UAV‐based detection is particularly useful for tactical monitoring, but also requires models that can handle small targets, smoke interference, illumination changes, and imbalanced fire‐background pixels.

Object‐detection and lightweight deep‐learning models have further expanded active‐fire and smoke detection toward real‐time and edge deployment. You Only Look Once (YOLO)‐based models have been adapted to detect flames and smoke in complex image scenes, while lightweight CNNs have been designed for embedded systems. He et al. (2024) proposed DCGC‐YOLO to improve early fire detection by enhancing bottleneck structures and anchor design, while Chaturvedi et al. (2025) developed a multiattention interlaced network for early wildfire smoke detection from satellite images under complex atmospheric conditions. Alam et al. (2025) introduced FireNet‐CNN with explainable AI tools such as Gradient Class Activation Maps (Grad‐CAM) to improve transparency in real‐time forest‐fire detection. These approaches indicate that modern active‐fire detection is moving beyond simple classification toward efficient object detection, semantic segmentation, smoke recognition, and explainable decision support.

Benchmark data sets have become essential for improving model generalization and comparing fire‐detection algorithms across platforms. M. Wang et al. (2025) introduced Flame And Smoke Detection Data set (FASDD), a cross‐domain flame and smoke detection data set with ground‐based, UAV, and satellite subsets, enabling evaluation across different observation scales. de Almeida Pereira et al. (2021) similarly provided a large Landsat‐8 active‐fire data set, while Das et al. (2026) reviewed recent computer‐vision‐based wildfire detection and emission‐quantification studies and highlighted the importance of standardized data sets for detection, segmentation, and downstream emission modeling. These dataset‐focused studies are important because many fire‐detection models previously relied on small or inconsistent data sets, limiting their transferability across regions, sensors, and fire conditions.

Wildfire spread forecasting has also advanced from empirical and physics‐based models toward machine‐learning surrogates and spatiotemporal deep‐learning frameworks. Hodges and Lattimer (2019) used a CNN to approximate Rothermel‐ and FARSITE‐generated fire‐spread simulations, showing that DL can serve as a fast surrogate for computational fire‐spread models. Huot et al. (2022) introduced the Next Day Wildfire Spread data set, which combines two‐dimensional fire progression with topography, vegetation, weather, drought, and population variables to support next‐day spread prediction. Cheng et al. (2022) combined physics‐based simulations, reduced‐order modeling, and satellite observations to enable both forward burned‐area prediction and inverse parameter estimation. These studies show that ML is increasingly being used not only to detect fires but also to approximate and accelerate physical fire‐spread simulation.

Recent deep‐learning spread‐forecasting models increasingly incorporate temporal context, multimodal predictors, and attention mechanisms. Marjani et al. (2026) developed a CNN‐BiLSTM model using VIIRS active‐fire data and environmental variables to predict daily wildfire spread by combining spatial feature extraction with temporal learning. Masrur et al. (2024) used attention‐based ConvLSTM networks to model wildfire spread dynamics, emphasizing the need to capture both local fire‐front propagation and longer‐range spread processes such as spotting. Anastasiou et al. (2026) showed that including temporal information before and after ignition improves burned‐area extent prediction, while Abbas et al. (2026) introduced FireCast‐Fusion, which combines UAV RGB–thermal imagery with environmental variables and physics‐guided learning to estimate near‐term fire‐front arrival time. These studies demonstrate that spread forecasting is moving toward spatiotemporal, physics‐aware, and multimodal deep‐learning systems.

Overall, active‐fire detection and wildfire spread forecasting have progressed from threshold‐based satellite methods and empirical spread equations to deep‐learning segmentation, object detection, multisensor fusion, UAV monitoring, benchmark data sets, and physics‐guided spatiotemporal forecasting. Active‐fire detection benefits from high‐frequency satellite observations, high‐resolution UAV imagery, and lightweight CNN or transformer‐based models, while spread forecasting increasingly relies on remote‐sensing time series, environmental predictors, simulation‐generated training data, and data assimilation. Despite these advances, key challenges remain, including cloud and smoke contamination, false alarms, small‐fire detection, class imbalance, sensor‐resolution trade‐offs, computational constraints, uncertainty propagation, and limited model transferability across ecosystems and fire regimes. Future work should integrate multisensor observations, explainable AI, uncertainty‐aware forecasting, edge deployment, and physics‐guided learning to build operational wildfire monitoring systems that are accurate, fast, interpretable, and useful for emergency decision‐making.

### 5.4 Burn Severity and Recovery Assessment

Post‐fire assessment refers to the evaluation of wildfire‐induced ecological and environmental changes following fire events, including burn severity, vegetation mortality, ecosystem degradation, soil disturbance, carbon loss, and post‐fire recovery dynamics. These assessments are essential for understanding wildfire impacts on ecosystems, guiding restoration planning, prioritizing management interventions, and evaluating ecosystem resilience under changing climate conditions. Post‐fire assessment operates across multiple temporal scales, ranging from immediate fire damage characterization to long‐term monitoring of vegetation regrowth and carbon recovery trajectories. Advances in remote sensing, AI, ML, and DL have substantially improved the efficiency, spatial coverage, and timeliness of post‐fire assessments, enabling large‐scale and near‐real‐time analysis of wildfire impacts.

Burn severity assessment is a major component of post‐fire analysis because it quantifies the degree of ecological damage caused by wildfire events. Traditionally, burn severity mapping has relied on satellite‐derived spectral indices, particularly the NBR, differenced NBR (dNBR), relative differenced NBR (RdNBR), NDVI, and related shortwave‐infrared‐based indicators. These indices are effective because fire alters vegetation structure, canopy moisture, soil exposure, and surface reflectance, especially in the near‐infrared and shortwave‐infrared regions. However, fixed threshold values may not transfer well across ecosystems, vegetation types, topographic settings, and fire regimes. To address this limitation, studies have proposed adaptive and site‐specific thresholding approaches. Kadakci Koca (2023), for example, showed that site‐specific statistical thresholding of Landsat‐8 spectral indices can improve burn‐severity class separation when compared with fixed thresholds. Similarly, Lasaponara et al. (2019, 2018) used Sentinel‐2 indices and unsupervised approaches to automatically map burned areas and burn‐severity levels, reducing dependence on manually defined thresholds.

Unsupervised learning has been particularly useful where field‐based burn severity data are limited. Holden and Evans (2010) demonstrated that fuzzy C‐means clustering and local spatial autocorrelation applied to dNBR images can produce burn‐severity classes comparable to field‐calibrated approaches, especially for moderate‐ and high‐severity classes. Negri et al. (2022) extended this idea by combining multitemporal and multispectral imagery with probabilistic unsupervised learning to map burned areas in Brazil and Bolivia. Such approaches are valuable because they reduce the need for extensive field calibration while still providing spatially coherent burned‐area products. They are especially relevant for rapid post‐fire assessment, where timely mapping is often more important than fully field‐calibrated severity classification.

Field‐based information remains important for validating and improving burn‐severity products. The Composite Burn Index has frequently been used as a ground reference to evaluate satellite‐derived burn severity. Karau et al. (2014) integrated RdNBR with outputs from the Fire Hazard and Risk Model and showed that combining satellite imagery with simulation modeling improved the explanation of field‐based burn severity relative to either source alone. This type of integration is important because satellite indices capture spectral change, while fire‐effects models can represent ecological processes and landscape‐scale fire behavior. Van and Lee (2025) further emphasized the importance of robust validation by comparing tree‐based regression models under spatially blocked validation schemes. Their results showed that models performing well within a fire event may not necessarily transfer well to unseen fires, highlighting the need for cross‐fire validation in operational burn‐severity mapping.

Recent studies have increasingly adopted ML and explainable AI approaches to improve burned‐area detection and severity classification. Gündüz et al. (2025) combined Sentinel‐2 spectral indices with ML models and SHAP analysis to classify burned and unburned areas and interpret the role of key predictors. Their results showed that ML models can outperform spectral‐index‐only approaches, while SHAP can identify influential variables such as dNBR, differenced NDVI (dNDVI), and short‐wave infrared (SWIR)/NIR bands. This illustrates a broader shift in post‐fire assessment: models are no longer evaluated only by classification accuracy but also by their interpretability and usefulness for management decisions. Explainable AI is particularly important in burn‐severity mapping because managers need to understand whether model outputs are driven by ecologically meaningful variables or by artifacts such as topographic shadow, background reflectance, or pre‐fire vegetation differences.

High‐resolution optical imagery and DL segmentation models have also improved burned‐area mapping. Cho et al. (2022) used PlanetScope imagery and a U‐Net‐based model to map burned areas from single‐date high‐resolution imagery, showing that fine spatial detail can support accurate delineation of burned patches when combined with vegetation indices, texture features, land‐cover information, and topographic normalization. DL architectures are especially useful for post‐fire mapping because burned areas often have irregular boundaries, heterogeneous severity patterns, and mixed pixels along fire perimeters. However, high‐resolution imagery also requires careful preprocessing to reduce shadow effects, topographic distortions, and spectral confusion with non‐burned dark surfaces.

Multisensor fusion has become another important direction in post‐fire burned‐area mapping. Optical imagery provides strong spectral information, while radar imagery can support mapping under cloud, smoke, or low‐visibility conditions. Khankeshizadeh et al. (2024) developed a dual‐path attention residual U‐Net that integrates Sentinel‐1 radar and Sentinel‐2 optical imagery for forest burned‐area detection. Xi et al. (2024) similarly proposed a dual‐path attention model using post‐fire Sentinel‐1 and Sentinel‐2 imagery, showing that optical–SAR fusion can preserve geometric details while improving burned‐area identification. Rana et al. (2026) further explored SAR‐based burned‐area mapping by comparing dual‐polarized vegetation indices, log‐ratio features, and U‐shaped deep‐learning architectures. These studies show that combining optical and radar observations can improve the robustness of burned‐area mapping, especially where clouds, smoke, or atmospheric conditions limit optical observations.

Beyond classification, recent work has also focused on improving the spatial quality of post‐fire imagery. Esmaeili et al. (2025) developed a super‐resolution framework for Sentinel‐2 imagery that uses burned‐area indicators to guide the generation of higher‐resolution images. This type of method is important because moderate‐resolution satellite imagery may not capture fine‐scale burn patterns, especially in heterogeneous landscapes or fragmented burned areas. By improving spatial detail, super‐resolution models can support more precise burned‐area delineation, better severity mapping, and improved assessment of post‐fire damage at management‐relevant scales.

Post‐fire assessment also extends beyond burned‐area and burn‐severity mapping to include secondary hazards and recovery processes. Friedel (2011) used a self‐organizing map to model post‐fire hydrologic and geomorphic hazards across burned basins in the western United States, linking burn conditions with runoff, landslide, debris‐flow, and flood responses. Indeed, post‐fire assessment is not only about mapping burn scars but also about forecasting subsequent landscape instability and recovery constraints. Orland et al. (2025) advanced near‐real‐time severity assessment by using VIIRS‐based active‐fire tracking, FRP, and rate‐of‐spread metrics to estimate burn severity before final fire containment. Such approaches can provide early indicators of post‐fire risk and help managers anticipate erosion, debris flows, water‐quality impacts, and rehabilitation needs.

Overall, burn severity and recovery assessment has progressed from index‐based thresholding toward adaptive, machine‐learning, deep‐learning, multisensor, and near‐real‐time frameworks. Spectral indices such as NBR, dNBR, RdNBR, NDVI, and dNDVI remain foundational because they provide physically interpretable measures of vegetation and surface change. However, recent studies show that adaptive thresholding, unsupervised clustering, ML, explainable AI, optical–SAR fusion, super‐resolution, and active‐fire tracking can substantially improve post‐fire mapping and interpretation. Despite these advances, several challenges remain, including threshold transferability, field‐data scarcity, sensor‐resolution limits, spectral confusion, cloud and smoke contamination, uncertainty propagation, and limited understanding of long‐term recovery trajectories. Future work should integrate multisensor time series, field validation, explainable modeling, near‐real‐time severity indicators, and ecosystem recovery metrics to produce post‐fire assessments that are timely, accurate, interpretable, and directly useful for restoration planning and hazard mitigation.

### 5.5 Subseasonal‐to‐Seasonal (S2S) Forecasts for Wildfires

Subseasonal‐to‐seasonal (S2S) forecasts at time scales from 2 weeks to a season offer critical lead times for wildfire management (Turner et al., 2024). They enable proactive resource allocation, prescribed burning, and community preparedness by anticipating fire‐conducive conditions such as elevated temperatures, low humidity, and drought (Di Giuseppe et al., 2020). As climate change lengthens fire seasons and intensifies their severity, S2S forecasts are an increasingly vital decision‐support tool (Abatzoglou et al., 2023; Y.Chen et al., 2020 ; Nikonovas et al., 2021; Turner et al., 2024). Recent advancements in S2S forecasting of wildfire risks integrate dynamical models, statistical approaches, and ML, and leverage teleconnections such as the El Niño‐Southern Oscillation (ENSO) and the boreal summer intraseasonal oscillation (BSISO) to enhance predictability (Y.Chen et al., 2020 ). Given that increased wildfire risk is a function of high temperatures, low humidity, and drought, using S2S forecasts of these conditions provides critical information for contingency planning and prescribed burning.

#### 5.5.1 Methods in S2S Wildfire Forecasting

S2S wildfire forecasting systems are based either on dynamical climate modeling, statistical and machine‐learning approaches, or multi‐source data assimilation (Y.Chen et al., 2020 ; Turner et al., 2024). Dynamical models constitute the physical foundation. They simulate the coupled atmosphere–land–ocean system under initial and boundary conditions and capture both short‐term variability and slowly evolving teleconnections such as the Madden–Julian Oscillation and ENSO (Turner et al., 2024). NOAA's Climate Forecast System version 2 (CFSv2) and ECMWF's SEAS5 generate ensemble forecasts of meteorological drivers that are critical for fire‐risk assessment, such as temperature, precipitation, wind, and humidity (Turner et al., 2024). When these forecasts are bias‐corrected and downscaled using observationally constrained products such as gridMET, they can be translated into operational fire‐danger indices, such as the Energy Release Component (ERC), the Burning Index, and the 100‐hr Fuel Moisture (FM100) (Abatzoglou et al., 2023). Recent evaluations show statistically significant skill for ERC up to 3 weeks ahead across large parts of the western United States, with ensemble reliability persisting to the fourth week, particularly in transition seasons when atmospheric persistence and fuel dryness co‐vary (Abatzoglou et al., 2023).

Dynamical S2S systems, such as the European Center for Medium‐Range Weather Forecasts (ECMWF) Seasonal Forecasting System 5 (SEAS5), provide global probabilistic forecasts of fire danger indices, including the Canadian FWI, U.S. National Fire‐Danger Rating System, and Australian McArthur Mark 5 (Di Giuseppe et al., 2020). These models demonstrate skillful predictions of anomalous fire weather up to 1–2 months ahead globally, with extended horizons to 6–7 months during strong ENSO or Indian Ocean Dipole events, as validated against ERA5 reanalysis data (Di Giuseppe et al., 2020). For instance, in regions such as Indonesia and Australia, such forecasts have informed responses to extreme events, such as the 2019–2020 Black Summer fires, by supporting early land management decisions (Nikonovas et al., 2021). S2S forecasts have shown promise in high‐risk areas such as the Pacific Northwest (PNW), where BSISO phases 6–7 increase heatwave and fire weather probabilities by 50%–120%, driven by tropical convection anomalies that propagate Rossby waves and induce anticyclonic ridges (Abatzoglou et al., 2023). Analysis of the unprecedented 2021 PNW heatwave revealed that ECMWF S2S ensembles show elevated risks of extreme temperatures and atmospheric blocking up to 11 days in advance, highlighting links to heightened wildfire ignition via cured vegetation and extreme FWI values (Abatzoglou et al., 2023).

Hybrid methods of forecasting integrate dynamical output with machine‐learning/statistical post‐processing to improve downscaling, extreme event detection, and operational utility by fusing mechanistic physics‐based understanding with data‐driven pattern recognition. Such physics‐informed and adaptive approaches have improved subseasonal prediction of key fire‐related variables. Predictor variables in these systems include meteorological factors (e.g., temperature, precipitation, wind speed, humidity from forecast models), fuel and vegetation metrics (e.g., live/dead biomass, moisture content, greenness indices), climate teleconnections (e.g., ENSO, PDO, soil moisture anomalies), and human/land use elements (e.g., ignition likelihood, land cover). However, the latter are less commonly integrated (Turner et al., 2024). Data sources span satellite observations (e.g., MODIS and VIIRS for active fires and vegetation indices), reanalysis products (e.g., ERA5 for historical climate context), and ground networks (e.g., weather stations for validation), forming a comprehensive input framework for robust S2S predictions. This multi‐source assimilation ensures that forecasts account for both large‐scale climate variability and local fire‐weather influences.

ML innovations further advance S2S wildfire forecasting, particularly in the western U.S. The SMLFire model, a stochastic mixture density network using 51 predictors (e.g., vegetation, topography, and climate indices) at 12 km resolution, captures monthly burn variations and trends. However, it underperforms for extremes (Randerson et al., 2021). Building on this, SEASFire integrates SEAS5 ensembles with SMLFire to produce probabilistic forecasts. SEASFire accurately identified 2022–2023 risks in the PNW and enabled decomposition of uncertainty between climate and fire components (Li et al., 2022). Statistical approaches such as autoregressive integrated moving‐average with exogenous variables (ARIMAX) have been applied globally, explaining 52% of fire emissions anomalies with a 1‐month lead. Ocean climate indices and VPD were used as predictors across over 1,300 fire regions. The model outperformed persistence baselines—however, skill declines at longer leads (Y.Chen et al., 2020 ).

DL approaches are emerging as powerful tools for global S2S wildfire forecasting. A segmentation‐based model treats wildfire prediction as an image segmentation task, using a global datacube of climate, vegetation, and oceanic indices to forecast the presence of burned areas 8–64 days ahead with high skill (Prapas et al., 2022). In the contiguous U.S., downscaled S2S forecasts from NOAA's CFSv2 show significant week‐3 skill for ERC (76% of area) and evaporative demand (30%) (Abatzoglou et al., 2023). NOAA workshops emphasize interagency collaboration to advance S2S wildfire outlooks, highlighting the need for integrating AI with physical models (United States National Weather Service, 2021).

Despite these progresses, challenges persist, including biases in dynamical models, underestimation of extremes, and limited integration of human factors like ignition sources (Randerson et al., 2021). Future directions emphasize hybrid systems combining ML with physics‐based models, improved downscaling to enhance regional skill, and leveraging teleconnections to extend predictability (Zhu et al., 2022). Such enhancements could bolster wildfire resilience by providing actionable insights for stakeholders.

## 6 Advances in Remote Sensing for Wildfire Detection and Assessment

Remote sensing serves as the primary observational backbone of integrated wildfire decision support systems, providing repeated spatial measurements of surface conditions, thermal activity, smoke emissions, burned landscapes, and post‐fire ecosystem change. Compared with ground‐based observations, remote sensing enables consistent monitoring across physically inaccessible terrain, large geographic regions, and rapidly evolving fire environments. Satellite, airborne, and UAV‐based platforms therefore play essential roles in observing wildfire‐relevant surface and atmospheric conditions, including pre‐fire vegetation stress and fuel conditions, active fire location, intensity, and smoke‐plume behavior, and post‐fire burned‐area extent, burn severity, and ecosystem recovery. Commonly used satellite systems, including MODIS, AVHRR, Landsat, VIIRS, and Sentinel, provide multispectral observations across visible, NIR, SWIR, and thermal infrared wavelengths, while UAVs offer flexible, high‐resolution observations for localized fire analysis and validation (Kallimani et al., 2014; Mohapatra & Trinh, 2022; Ramos et al., 2024).

The role of remote sensing differs substantially across wildfire phases because each stage requires distinct observational objectives and sensor capabilities. During the pre‐fire stage, remote sensing products are commonly used to characterize vegetation greenness, vegetation water content, live fuel moisture, drought stress, surface temperature, and fuel accumulation patterns that influence wildfire susceptibility. During active burning, thermal infrared observations, hotspot detection, FRP retrievals, smoke‐plume tracking, and fire‐perimeter mapping are critical for near‐real‐time situational awareness and emergency response. Following wildfire events, post‐fire remote sensing assessments rely on spectral, thermal, and structural information to quantify burned‐area extent, burn severity, vegetation mortality, soil exposure, canopy loss, and ecosystem recovery trajectories. Consequently, no single satellite platform can optimally support all wildfire applications, and sensor selection requires balancing spatial, temporal, spectral, and radiometric trade‐offs according to specific operational objectives.

Table 1 summarizes major satellite platforms and their corresponding wildfire applications, ranging from long‐term open‐access systems such as Landsat and MODIS to newer high‐resolution and commercial sensors such as WorldView. These systems differ considerably in revisit frequency, spatial resolution, thermal sensitivity, spectral coverage, and operational continuity, leading to important trade‐offs in wildfire monitoring performance. For example, MODIS established a long‐term global baseline for hotspot detection and FRP estimation through its high temporal coverage and continuous historical archive, whereas the next‐generation VIIRS sensor improved the detection of smaller and lower‐intensity fires through enhanced 375‐m spatial resolution and reduced pixel distortion effects. Similarly, geostationary systems such as GOES provide high‐frequency temporal observations suitable for rapid fire progression and smoke monitoring, while Landsat and Sentinel‐2 offer finer spatial detail for burned‐area mapping, burn severity assessment, and vegetation recovery analysis.

**Table 1:** Summary of Satellite Remote Sensing Products and Their Characteristics, Applications, and Indices for Wildfire Monitoring and Management

Product name	Spatial resolution	Temporal resolution	Spectral resolution	Radiometric	Durations	Key applications and indices
Landsat	30 m	8–16 days	Visible, NIR, SWIR, TIR	16‐bit	1972*–Present	Longest continuous record of space‐based surface observations.
						Post‐fire analysis
						NDVI, NDWI, NBR, dNBR, RdNBR
Sentinel‐2	10 m, 20 m, 60 m	∼5 days	13 Bands	12‐bit	2015–Present	Precise fire detection
						NDVI, NDWI, NBR, dNBR, RdNBR
MODIS	250 m–1 km	1–2 days	36 Bands	12‐bit	2000–Present	Source for active fire products
(Terra/Aqua)						NDVI, EVI, FRP, thermal anomaly detection
VIIRS	375–750 m	12 hr	22 Bands	14‐bit	2012–Present	Component of S2S forecasting frameworks
						FRP, active fire detection, thermal anomaly detection
GOES‐R	0.5–2 km	5–15 min	16 Bands	14‐bit	2016–Present	Detecting early thermal anomalies before ground reports
						Monitoring plume development, smoke transport, and PyroCb events.
						FRP
AVHRR	1.1 km	1 day	5‐6 Bands	10‐bit	1981–Present	Long‐term historical trend analysis
WorldView (2/3/4)	0.31–1.24 m	<1–4 days	8‐16 Bands	11/14‐bit	2009–Present	VHR post‐fire assessment.
						Damage mapping of buildings/infrastructure.
						Precision vegetation recovery

Despite major advances in remote sensing technologies, wildfire monitoring products remain subject to substantial operational and observational constraints. Detection accuracy may be affected by cloud cover, dense smoke, canopy obstruction, terrain complexity, sensor saturation, viewing geometry, mixed pixels, and differences in overpass timing. Furthermore, trade‐offs between spatial and temporal resolution can influence the sensitivity of fire detection systems, particularly for low‐intensity, short‐duration, or sub‐canopy fires. These limitations have accelerated the transition from single‐sensor approaches toward integrated multi‐satellite constellations and experimental high‐resolution missions such as FireBIRD, which aim to improve global wildfire monitoring through complementary spatial, temporal, spectral, and thermal observations (Nolde et al., 2021; Roy et al., 2024). The following subsections further examine remote sensing applications across pre‐fire fuel and vegetation monitoring, active fire and smoke observation, post‐fire spectral and structural assessment, smoke and air‐quality estimation, and the associated validation and operational challenges.

### 6.1 Pre‐Fire Fuel and Vegetation Stress

Pre‐fire monitoring through remote sensing is essential for characterizing fuel conditions and other environmental factors that influence wildfire ignition potential and subsequent fire behavior. Because field measurements of fuel moisture and vegetation condition are spatially sparse, satellite and airborne observations provide a practical way to monitor wildfire‐prone landscapes across broad spatial scales. Pre‐fire remote sensing primarily focuses on live fuel moisture, dead fuel moisture, canopy water stress, drought condition, biomass accumulation, fuel continuity, and vegetation structure. These variables influence both ignition likelihood and the potential intensity and/or severity of subsequent fires. Multispectral, thermal, microwave, and LiDAR observations therefore provide complementary information for wildfire risk assessment, fire‐danger monitoring, and early warning systems (Watt et al., 2025).

Early pre‐fire fuel monitoring focused largely on estimating live fuel moisture from optical reflectance. Danson and Bowyer (2004) showed that water‐sensitive spectral indices can be related to live fuel moisture, although retrieval accuracy depends on leaf structure, dry matter content, canopy properties, and vegetation type. Hao and Qu (2007) developed a semi‐physical MODIS‐based approach for near‐real‐time live fuel moisture retrieval, showing that multispectral satellite observations could support fire‐danger rating over large areas. These early studies established the foundation for later operational fuel moisture products based on optical satellite observations.

The next stage involved regional live fuel moisture monitoring using long‐term satellite records. Myoung et al. (2018) used MODIS vegetation indices to estimate live fuel moisture and showed that satellite‐derived vegetation dynamics can capture seasonal and interannual moisture variation. Argañaraz et al. (2018) used satellite‐derived fuel moisture and fire records to define land‐cover‐specific thresholds for fire hazard classification, emphasizing the need for locally calibrated early‐warning thresholds. García et al. (2020) developed a Landsat‐based live fuel moisture product for fire behavior modeling, showing that spatially variable fuel moisture can alter simulated fire spread and burned probability compared with spatially constant fuel‐moisture assumptions. These studies shifted pre‐fire monitoring from simple vegetation indices toward spatially explicit fuel moisture products that can support fire‐danger and fire‐behavior applications.

Higher‐resolution optical sensors then improved fuel moisture estimation in heterogeneous vegetation conditions. Marino et al. (2020) compared empirical models and radiative‐transfer simulations and showed that empirical models based on Sentinel‐2 and MODIS can provide practical live fuel moisture estimates in fire‐prone shrub and forest canopies. Costa‐Saura et al. (2021) combined Sentinel‐2 Normalized Difference Moisture Index with meteorological variables to estimate live fuel moisture in mixed vegetation conditions. Tanase et al. (2022) evaluated active and passive sensors and showed that optical data, especially when combined with vegetation‐cover information, can outperform radar alone for live fuel moisture estimation in some vegetation types. Cunill Camprubi et al. (2022) extended regional mapping by combining MODIS spectral bands, vegetation indices, land surface temperature, seasonal predictors, and Random Forest models to generate operational fuel moisture products. Together, these studies show that live fuel moisture estimation improves when spectral indices are combined with meteorological, seasonal, and vegetation‐structure information.

Microwave and radar observations have become increasingly important because they provide information on vegetation and surface moisture under conditions where optical imagery may be limited by cloud cover, smoke, or canopy effects. Pirotti et al. (2023) showed that Sentinel‐1 backscatter can distinguish wet and dry canopy conditions and can also capture canopy response before and after fire. Cho et al. (2025) used SMAP observations to retrieve vegetation water content as a proxy for live fuel moisture, showing the value of L‐band microwave observations for fuel moisture monitoring. Bourgeau‐Chavez et al. (2026) further demonstrated that SMAP reflectivity can provide useful information on organic fuel moisture, although standard soil moisture products may be biased in some ecosystems. These studies indicate that microwave observations can complement optical indices, but ecosystem‐specific calibration remains necessary.

Dead fuel moisture content has also become an important focus of pre‐fire remote sensing because dry fine fuels, litter, and forest‐floor material strongly influence ignition and early fire spread. Xing et al. (2023) used UAV multispectral imagery and DL to estimate forest‐floor dead fuel moisture at fine spatial scales. Fan et al. (2024) improved dead fuel moisture estimation by incorporating geostationary satellite‐derived humidity into a process‐based fuel moisture model. Li et al. (2024) used Landsat imagery and Random Forest models to estimate understory fine dead fuel moisture by combining meteorology, topography, vegetation indices, precipitation, and seasonal factors. Fan et al. (2026) advanced this direction using a physics‐guided LSTM framework that integrates geostationary satellite observations, reanalysis data, and fuel‐moisture physics for hourly dead fuel moisture estimation. These studies show a shift from static moisture mapping toward multiscale and physics‐informed fuel moisture monitoring.

Recent work has expanded pre‐fire assessment from fuel moisture estimation to broader vegetation stress and fuel‐structure characterization. Fang et al. (2018) showed that remotely sensed surface moisture availability, vegetation, and topography can explain potential fire severity, confirming that pre‐fire moisture conditions influence later burn intensity. Le et al. (2025) used long‐term Landsat observations and canopy dryness indices to monitor forest water stress under drying climates. Zhu et al. (2025) used ECOSTRESS‐derived water‐use efficiency and evaporative stress to link ecosystem water limitation with wildfire occurrence during extreme fire conditions. These studies demonstrate that pre‐fire drought stress and vegetation water limitation are not only related to ignition risk but also influence subsequent fire behavior and severity.

Fuel structure is another key component of pre‐fire hazard because canopy layering, ladder fuels, and horizontal and vertical fuel continuity affect fire spread and severity. Hakkenberg et al. (2024) showed that spaceborne LiDAR can characterize fuel structure and that ladder fuels may predict wildfire severity more consistently than canopy volume metrics. Torralba et al. (2026) used multi‐wavelength terrestrial laser scanning to estimate live fuel moisture by combining spectral and structural information at the plot scale. These lidar‐based approaches are important because they provide three‐dimensional fuel information that is difficult to obtain from optical imagery alone.

Finally, pre‐fire fuel and vegetation stress products are increasingly connected to predictive fire danger and early warning. Miller et al. (2023) used DL to project live fuel moisture several months in advance, moving from current fuel‐condition monitoring toward predictive fuel‐stress forecasting. McNorton et al. (2024) developed a probability‐of‐fire forecasting system that integrates satellite observations, vegetation characteristics, and meteorological forecasts to provide dynamic fire‐risk predictions. Overall, pre‐fire remote sensing has progressed from optical fuel moisture indices toward integrated, multisensor, and forecast‐oriented systems. Future progress will depend on combining optical, thermal, microwave, lidar, UAV, field, and meteorological data within physically informed and interpretable modeling frameworks that can support operational wildfire risk assessment and fuel management.

### 6.2 Real‐Time Fire Monitoring

Satellite remote sensing plays a central role in real‐time wildfire monitoring by observing thermal anomalies, tracking fire activity, and supporting tactical decision‐making during rapidly changing fire events (Koltunov et al., 2016). Because satellite observations are constrained by spatial resolution, thermal sensitivity, overpass timing, and atmospheric interference, they generally identify detectable thermal anomalies and fire hotspots rather than ignitions (Schroeder et al., 2010). However, recent studies have used higher temporal resolution sensors to detect ignitions of larger fire complexes (Scholten et al., 2026). Thermal infrared observations from satellite platforms enable repeated monitoring of fire activity across large geographic regions, providing critical situational awareness for emergency response and fire management operations (Xu & Zhong, 2017). Enhanced image‐processing algorithms and AI‐assisted detection systems further improve the accuracy and timeliness of fire identification under complex environmental conditions, including heterogeneous land surfaces, smoke contamination, and rapidly changing fire behavior (Zhao & Ban, 2022).

Early real‐time fire monitoring primarily focused on detecting active fire pixels and quantifying fire intensity from thermal anomalies. Wooster et al. (2003) established FRE as a physically meaningful satellite‐derived measure of biomass burning and combustion intensity. Ichoku et al. (2008) later used MODIS FRP observations to characterize global fire intensity patterns and classify active fires by fire strength. Schroeder et al. (2010) showed that FRP, fire area, and fire temperature estimates can be affected by sensor resolution, background characterization, subpixel fire structure, and omission of small fire lines. These early studies were important because they moved active fire monitoring beyond simple fire/no‐fire detection toward quantitative characterization of fire intensity and biomass burning.

The next major development was the use of geostationary satellites for faster fire detection and tracking. Koltunov et al. (2016) developed the GOES Early Fire Detection algorithm to reduce the time between fire development and first satellite detection using contextual hotspot tests, dynamic event classification, and tracking. Xu and Zhong (2017) demonstrated that Himawari‐8 observations can be used to monitor wildfire spread direction and rate in near real time because of their high temporal frequency. Li et al. (2018) further showed that geostationary and polar‐orbiting FRE retrievals provide complementary information for estimating biomass consumption. These studies established the operational value of geostationary satellites for early fire detection, continuous fire tracking, and fire‐intensity monitoring.

As finer‐resolution sensors became more widely used, real‐time monitoring expanded from coarse thermal hotspot detection to more detailed active fire and burned‐area mapping. Oliva and Schroeder (2015) showed that VIIRS 375 m active fire detections can be aggregated to support direct burned‐area mapping, particularly for long‐duration fires. Briones‐Herrera et al. (2020) used aggregated MODIS and VIIRS active fire detections with convex hull methods to estimate early large‐fire perimeters before standard burned‐area products became available. Sousa et al. (2020) also showed that thermal cameras can be integrated into robotic and UAV‐based perception systems for localized autonomous fire surveillance and early warning. These developments demonstrated that real‐time fire monitoring can support both regional satellite‐based products and finer‐scale tactical monitoring systems.

Recent work has increasingly used medium‐resolution optical sensors and DL to improve active fire detection. Hu et al. (2021) proposed a Sentinel‐2 multi‐criteria active fire algorithm using visible, near‐infrared, and shortwave‐infrared bands with adaptive thresholds to detect active fires at 20 m resolution. Q. Zhang et al. (2021) developed a Sentinel‐2 DL framework using an attention‐enhanced HRNet architecture to automate active fire detection from SWIR–NIR imagery. P. Zhang et al. (2021) fused Sentinel‐1 SAR and Sentinel‐2 optical time series with a U‐Net framework to update wildfire progression maps when new observations became available. These studies show that medium‐resolution optical and SAR data can improve fire‐front mapping and wildfire progression monitoring, although revisit frequency remains lower than that of geostationary satellites.

The integration of geostationary time series with ML has further improved early fire detection. Zhao and Ban (2022) used GOES‐R time‐series observations with a Gated Recurrent Units (GRU)‐based deep‐learning network to detect early‐stage wildfires more rapidly than many polar‐orbiting active fire products. D. Zhang et al. (2023) combined VIIRS fire‐product labels with Himawari‐8 multiband observations in a Random Forest framework to improve real‐time fire detection and small‐fire identification. Padilla et al. (2026) combined Sentinel‐2 reflectance and VIIRS active fire detections to generate near‐real‐time burned‐area maps with low latency. These approaches show that real‐time fire monitoring is increasingly shifting toward data fusion, where high‐frequency geostationary observations, high‐resolution optical imagery, and active fire detections are combined to improve both timeliness and spatial detail.

Recent studies have also improved the tracking of fire progression and persistence. Liu et al. (2024) developed the GOES‐Observed Fire Event Representation product using GOES active fire detections to reconstruct hourly wildfire progression and fill temporal gaps between polar‐orbiting satellite overpasses. Schiks et al. (2024) used MODIS and VIIRS active fire detections with geospatial interpolation to reconstruct day‐of‐burn progression for large wildfires. Coffield et al. (2026) showed that including additional VIIRS candidate fire pixels can improve tracking of persistent, smoldering, and partially obscured fire activity that may be missed by standard active fire products. These studies are important because they show that active fire detections can be used not only to locate fires but also to reconstruct fire growth, persistence, and combustion dynamics.

More recent algorithmic advances have focused on improving active fire detection under complex backgrounds and extracting more detailed fire information from high‐resolution imagery. Zhou et al. (2024) showed that adding Gray Level Co‐occurrence Matrix texture features to Sentinel‐2 spectral information improves Random Forest‐based active fire detection in heterogeneous land‐cover conditions. Xu and Wooster (2023) expanded operational FRP monitoring by describing and validating the Sentinel‐3 Sea and Land Surface Temperature Radiometer daytime active fire and FRP product against established MODIS, VIIRS, and Landsat data sets. Mazzeo et al. (2025) demonstrated that enhanced Normalized Hotspot Indices from Sentinel‐2 and Landsat 8/9 can improve active fire mapping, fire‐front identification, and small‐fire detection. Gui et al. (2025) developed a Sentinel‐2 and Landsat SWIR‐based method that reduces false detections from bright surfaces and industrial thermal anomalies while enabling fine‐scale active fire detection. Galván‐Hernández et al. (2025) further showed that long‐wave infrared imaging and spatial–temporal object detection can support early fire identification from ground‐based systems.

Overall, real‐time fire monitoring has progressed from coarse thermal anomaly detection toward integrated systems that detect active fires, track fire progression, map fire perimeters, and quantify fire intensity. Early developments established FRP and FRE as key indicators of fire intensity, while geostationary satellites improved temporal monitoring and early detection. More recent work has added medium‐resolution optical imagery, SAR–optical fusion, ML, DL, and multisensor active fire aggregation to improve fire‐front mapping and fire progression tracking. Future real‐time fire monitoring systems will likely depend on combining geostationary, polar‐orbiting, optical, thermal, microwave, and UAV observations with efficient and interpretable algorithms to support rapid wildfire response, fire behavior assessment, and emissions monitoring.

### 6.3 Post‐Fire Impact Assessment and Recovery

Satellite‐based post‐fire assessment provides critical information for evaluating wildfire impacts, monitoring ecosystem condition, and guiding recovery planning after fire events. Remote sensing enables spatially continuous mapping of fire‐affected landscapes, including burned‐area extent, burn severity, vegetation mortality, canopy loss, soil exposure, and post‐fire recovery trajectories. Compared with field‐based assessments alone, satellite observations provide broader spatial coverage and repeated temporal measurements, making them especially valuable for large, inaccessible, or rapidly changing burned landscapes. These capabilities support emergency damage evaluation, ecological restoration planning, watershed protection, and long‐term assessment of ecosystem resilience following wildfire disturbance.

Burned‐area mapping is one of the most widely used post‐fire remote sensing applications because it provides the spatial foundation for subsequent burn severity, erosion, recovery, and emissions analyses. Sparks et al. (2015) showed that burned‐area products such as Monitoring Trends in Burn Severity (MTBS) can contain commission errors in shrub–steppe environments, especially when unburned islands or fingers are included within fire perimeters. Picotte et al. (2020) further demonstrated that updates to MTBS mapping procedures can affect burn‐severity class distributions, emphasizing the importance of product version consistency for long‐term post‐fire analyses. Sismanis et al. (2023) developed an unsupervised Sentinel‐2 burned‐area mapping approach using adaptive thresholding, showing that automated methods can produce results comparable to operational emergency mapping products. Zhang et al. (2024) compared Sentinel‐2, Sentinel‐1, and ALOS‐2 PALSAR‐2 data for large‐scale burned‐area mapping and found that optical Sentinel‐2 data generally provided stronger burned–unburned separability than SAR alone. These studies show that burned‐area products are essential but must be carefully validated because mapping accuracy can vary with vegetation type, sensor resolution, fire size, and product design.

Burn severity assessment is another major component of post‐fire impact analysis because it links fire effects to ecological damage, vegetation mortality, soil alteration, and recovery potential. NBR, dNBR, and RdNBR remain among the most widely used spectral indices for evaluating fire‐induced vegetation and surface change. Heward et al. (2013) showed that FRP can help explain landscape‐scale burn severity, although per‐pixel comparisons between fire intensity and severity are limited by spatial and temporal undersampling. Cardil et al. (2019) calibrated RdNBR with field data and showed that satellite‐derived burn severity corresponds to field indicators such as char height, lichen loss, and vegetation‐cover change. Saulino et al. (2020) found that standard dNBR thresholds may perform poorly across heterogeneous forest types, while locally calibrated Sentinel‐2 thresholds substantially improved burn‐severity classification. Sobrino et al. (2019) linked Sentinel‐2 and Landsat spectral indices with field‐measured soil burn severity, showing that Sentinel‐2 dNBR can support rapid post‐fire soil severity assessment.

Several studies emphasize that burn‐severity indices are not universally transferable across ecosystems, sensors, or fire histories. Cai and Wang (2022) compared dNBR and RdNBR and found that dNBR performed better in their case study, indicating that RdNBR is not always a superior estimator of burn severity. van Gerrevink and Veraverbeke (2021) used hyperspectral Airborne Visible/Infrared Imaging Spectrometer (AVIRIS) data to evaluate the spectral sensitivity of dNBR and showed that optimal near‐infrared and shortwave‐infrared band combinations can differ from standard Landsat and Sentinel‐2 bands. D. Chen et al. (2020) demonstrated that Landsat‐derived dNBR can be affected by image‐pair selection, especially when pre‐ and post‐fire images differ in season or phenological condition. Chen et al. (2021) further showed that optimal Landsat image pairs are often limited in high northern latitude regions, reducing the consistency of dNBR, RdNBR, and relativized burn ratio products in some wildfire assessments. Together, these studies highlight that burn‐severity estimates depend not only on the selected index but also on sensor characteristics, image timing, field calibration, and ecological context.

The increasing availability of Sentinel‐2 has improved the spatial detail of post‐fire burn severity and vegetation impact assessment. Amos et al. (2019) showed that Sentinel‐2A shortwave‐infrared‐based indices were more effective than visible bands for distinguishing burned and unburned areas, supporting the use of Sentinel‐2 for fire severity detection. Howe et al. (2022) compared Sentinel‐2 and Landsat 8 using field calibration plots and found that Sentinel‐2 generally performed as well as or better than Landsat for burn‐severity mapping. Han et al. (2021) used Sentinel‐2‐derived leaf area index and fractional vegetation cover to evaluate short‐term vegetation response after fire, showing that these biophysical variables respond clearly to burn severity. These studies demonstrate that Sentinel‐2 can support both severity mapping and early vegetation recovery assessment because of its higher spatial resolution and useful spectral information. However, they also show that severity estimates from Sentinel‐2 and Landsat should be compared carefully because differences in spatial resolution can influence patch‐level severity patterns.

Post‐fire impact assessment increasingly extends beyond vegetation damage to include soil burn severity and secondary hazards such as erosion. Mallinis et al. (2009) integrated ASTER, Landsat, and GIS‐based modeling to assess post‐fire soil erosion risk and showed that severe erosion‐risk classes expanded substantially after fire. Falaras et al. (2022) combined Sentinel‐2 NBR‐based burn‐severity mapping with Revised Universal Soil Loss Equation erosion modeling in a geospatial web application for operational post‐disaster hazard assessment. Seydi et al. (2024) developed a physics‐informed machine‐learning model to estimate soil burn severity from vegetation burn severity and environmental covariates, supporting scalable post‐fire hydrologic hazard assessment. These studies are important because soil burn severity and erosion risk directly influence runoff, sediment transport, debris‐flow potential, water quality, and emergency stabilization priorities.

Vegetation recovery monitoring is another critical part of post‐fire assessment because it provides information on ecosystem resilience, restoration success, and long‐term ecological change. Bright et al. (2019) used Landsat time‐series analysis to track post‐fire NBR recovery across multiple forest types and showed that recovery varies with burn severity, forest type, and post‐fire climate conditions. Bousquet et al. (2022) used multi‐wavelength satellite observations to monitor recovery across vegetation biomes and showed that L‐band vegetation optical depth better captures woody biomass recovery than shorter‐wavelength indicators. Lee and Park (2026) reconstructed Sentinel‐2 NDVI time series using a double logistic model and found that baseline NDVI better represents structural recovery than peak or integrated NDVI, which can saturate quickly after understory regrowth. These time‐series approaches show that recovery cannot be fully captured by single‐date post‐fire images and requires repeated observations across multiple years.

Recent work has also shown that fire history and pre‐fire vegetation structure influence post‐fire severity and recovery. Saberi and Harvey (2023) found that the CBI and RdNBR can under‐ or overestimate burn severity in short‐interval reburns, depending on the severity of the prior fire and the vegetation components being assessed. Lee et al. (2024) showed that pre‐fire NDVI, canopy cover, and tree density can predict later burn severity, demonstrating the value of integrating pre‐fire vegetation structure into post‐fire severity assessment. Costa‐Saura et al. (2022) used Google Earth Engine and Random Forest models to map potential fire severity, showing how regional‐scale severity modeling can support risk analysis and post‐fire planning. These studies indicate that post‐fire impacts are shaped not only by the most recent fire event but also by prior disturbance history, pre‐fire vegetation condition, and landscape structure.

Overall, post‐fire remote sensing has progressed from basic burned‐area mapping toward integrated assessment of burn severity, soil burn severity, erosion risk, vegetation recovery, and ecosystem resilience. Optical indices such as NBR, dNBR, RdNBR, and NDVI remain widely used, but their reliability depends on sensor choice, field calibration, image timing, vegetation type, fire history, and ecological context. Newer approaches using Sentinel‐2, hyperspectral imagery, SAR, vegetation optical depth, lidar, time‐series reconstruction, ML, and physics‐informed ML modeling are improving the ability to quantify post‐fire impacts across scales. Future post‐fire assessment systems should integrate burned‐area products, field validation, multisensor time series, soil and hydrologic hazard models, fire history information, and recovery indicators to support emergency response, restoration prioritization, and long‐term ecosystem management.

### 6.4 Smoke, PM_2.5_, and Air‐Quality Monitoring

Remote sensing technologies play a vital role in monitoring wildfire‐generated air pollution across local, regional, and continental scales. Wildfire smoke can travel far beyond the fire perimeter, affecting visibility, atmospheric chemistry, public health, and transportation safety. Satellite observations are especially valuable because they provide repeated spatial coverage of smoke plumes, aerosol loading, and fire‐emission indicators in areas where ground‐based monitoring networks are sparse or unavailable. These observations support smoke‐plume tracking, air‐quality forecasting, exposure assessment, and public‐health warnings during active wildfire periods.

A major application is the monitoring of smoke transport and fine PM with aerodynamic diameter less than 2.5 μm, commonly referred to as PM_2.5_. Early studies combined satellite observations, surface monitoring, and chemical transport models to evaluate smoke transport and pollutant formation. Herron‐Thorpe et al. (2014) showed that wildfire air‐quality simulations in the Pacific Northwest captured general plume transport but underestimated surface PM_2.5_, aerosol optical depth (AOD), nitrogen dioxide, and plume‐top height. Baker et al. (2018) evaluated the 2013 Rim Fire and found that smoke‐plume transport was reasonably represented, but wildfire ozone was overestimated and organic carbon was underestimated near the fire. Yang et al. (2022) further showed that transported Canadian wildfire smoke increased ozone and particle pollution over the Mid‐Atlantic United States. These studies demonstrate that wildfire smoke affects both particulate pollution and atmospheric chemistry.

Satellite AOD is widely used for wildfire smoke monitoring because it represents the amount of aerosol loading in the atmospheric column. Geng et al. (2018) combined high‐resolution AOD, air‐quality model simulations, and ground observations to estimate daily PM_2.5_ during Colorado fire seasons. Xue et al. (2021) used AOD and meteorological information to estimate wildfire‐driven PM_2.5_ across the United States, showing that active fire years can increase PM_2.5_ over large regions far from the fire source. Mirzaei et al. (2018) integrated AOD with land‐use regression to estimate smoke‐related PM_2.5_ in southern Alberta, showing that separating pre‐fire, during‐fire, and post‐fire periods improves wildfire exposure assessment. Stowell et al. (2019) linked wildfire smoke PM_2.5_ exposure in Colorado with increased asthma and respiratory disease, highlighting the public‐health importance of satellite‐informed smoke exposure models.

AOD alone, however, does not directly indicate how much smoke reaches the surface. AOD measures total‐column aerosol loading, while human exposure depends on whether smoke is mixed into the near‐surface air. Cheeseman et al. (2020) showed that the PM_2.5_‐to‐AOD relationship changes with smoke plume height relative to the planetary boundary layer. T. Zhao et al. (2024) similarly found that aerosol vertical distribution is a major uncertainty when converting satellite AOD to surface PM_2.5_ during Alaskan wildfire seasons. Huang, Ji, et al. (2024) evaluated satellite plume‐height products using airborne LiDAR observations and showed that various products can represent different definitions of plume height. These studies show that plume height and boundary‐layer structure are essential for converting satellite smoke observations into reliable surface PM_2.5_ estimates.

Recent satellite products have improved the monitoring of rapidly changing smoke events. Huff et al. (2021) used geostationary satellite AOD to track a prescribed‐fire smoke plume and confirmed local PM_2.5_ degradation using surface monitors and vertical aerosol profiles. Ye et al. (2022) showed that updated satellite AOD retrievals performed better during the severe 2020 western U.S. wildfire season, especially under thick smoke conditions. Daniels et al. (2024) found that satellite AOD agreed well with ground‐based AOD during medium and heavy smoke, although mixed smoke–dust conditions and barren land introduced retrieval biases. These studies indicate that satellite AOD products are useful for wildfire smoke monitoring but must be evaluated carefully under dense smoke, complex surfaces, and mixed aerosol conditions.

Satellite observations are increasingly used to improve wildfire emission estimates. Anderson et al. (2023) showed that wildfire emission composition changes as fires evolve from flaming to smoldering combustion, suggesting that air‐quality models should not use constant emission factors throughout an event. Voshtani et al. (2025) used satellite and ground‐based carbon monoxide observations to show that several fire emission inventories underestimated emissions from the extreme 2023 Canadian boreal wildfires. Suo et al. (2025) developed an ultraviolet smoke index to improve smoke detection and particulate emission estimation under conditions where traditional AOD retrievals may fail. Li et al. (2025) used AOD and ultraviolet aerosol information to adjust wildfire emissions, reducing PM_2.5_ model bias during a major U.S. wildfire event.

Overall, wildfire smoke monitoring has advanced from simple plume detection toward integrated assessment of smoke transport, aerosol loading, plume height, emissions, PM_2.5_ exposure, and health impacts. AOD remains a central satellite variable, but it must be interpreted with information on plume height, boundary‐layer depth, smoke density, land surface conditions, and retrieval uncertainty. Future wildfire air‐quality monitoring should combine satellite observations, ground sensors, plume‐height information, air‐quality models, and health‐response data to improve smoke forecasts and exposure assessment during increasingly severe wildfire seasons.

### 6.5 Validation and Operational Constraints of Remote Sensing Products

Although remote sensing has greatly improved wildfire detection, monitoring, impact assessment, and smoke tracking, the operational use of satellite‐derived wildfire products remains constrained by validation uncertainty, sensor limitations, algorithm transferability, and data latency. Across pre‐fire fuel assessment, active fire detection, post‐fire burn severity mapping, and smoke‐related air‐quality monitoring, product reliability depends on how well satellite retrievals are calibrated against independent field, airborne, lidar, or ground‐based observations. These constraints are especially important because wildfire products are increasingly used for emergency response, public‐health warnings, ecosystem recovery planning, and long‐term climate‐risk assessment.

A major validation challenge is the limited availability of high‐quality field observations. In pre‐fire fuel and vegetation‐stress monitoring, satellite estimates of live fuel moisture content, dead fuel moisture content, and vegetation water stress require ground measurements for calibration. Costa‐Saura et al. (2021) showed that Sentinel‐2 vegetation indices and meteorological variables can estimate live fuel moisture content, but model performance depends on site‐specific vegetation and weather conditions. Tanase et al. (2022) further showed that optical Sentinel‐2 data generally outperformed Sentinel‐1 radar for live fuel moisture estimation in Mediterranean environments, indicating that sensor performance can vary with canopy structure and fuel type. Cho et al. (2025) demonstrated the value of Soil Moisture Active Passive observations for live fuel moisture retrieval, but also emphasized the difficulty of representing subgrid vegetation heterogeneity. These studies show that fuel‐moisture products are promising but require ecosystem‐specific validation before operational use.

Operational fire detection products face additional constraints related to spatial resolution, revisit frequency, cloud cover, smoke obscuration, and fire intensity. Geostationary sensors provide high temporal resolution and are useful for near‐real‐time monitoring, but their coarser spatial resolution can miss small or low‐intensity fires. Koltunov et al. (2016) improved early fire detection using geostationary observations, but detection performance still depended on background conditions and sensor sensitivity. Xu and Zhong (2017) showed that Himawari‐8 can support real‐time fire detection and tracking, but geostationary products remain affected by cloud contamination and mixed pixels. Hu et al. (2021) demonstrated that Sentinel‐2 can improve active fire detection because of its finer spatial resolution, but lower temporal frequency limits near‐real‐time operational use. These tradeoffs indicate that no single sensor fully satisfies the needs of both rapid detection and detailed fire characterization.

Fire radiative power and FRE products are also affected by retrieval uncertainty. Schroeder et al. (2010) showed that FRP estimates vary across MODIS, GOES, ASTER, and Landsat because of differences in spatial resolution, saturation, and overpass timing. Li et al. (2018) used FRE to estimate biomass combustion, but such approaches depend on accurate fire detection, fuel‐consumption assumptions, and emission coefficients. Dong et al. (2026) showed that missing FRP detections under heavy smoke or cloudy conditions can contribute to underestimation of smoke‐related PM_2.5_ in operational forecasts. These studies indicate that FRP products are valuable for emissions and smoke modeling, but they can propagate uncertainty into downstream air‐quality forecasts.

Post‐fire products face another set of validation constraints because burn severity indices are sensitive to field calibration, sensor choice, vegetation type, and image‐pair selection. Cardil et al. (2019) showed that RdNBR can be calibrated with field indicators of burn severity, but between‐fire variability remains important. Saulino et al. (2020) found that standard burn‐severity thresholds may not transfer well across Mediterranean forest types, while locally calibrated Sentinel‐2 thresholds improved classification accuracy. Sobrino et al. (2019) showed that Sentinel‐2 dNBR can estimate soil burn severity when validated with field observations. These studies demonstrate that burn‐severity products should not be applied with universal thresholds without local or regional validation.

Image selection is a major operational constraint in post‐fire assessment. D. Chen et al. (2020) showed that burn‐severity indices can be biased when pre‐ and post‐fire images differ in season or phenological condition. Chen et al. (2021) further showed that optimal Landsat image pairs are often limited in high northern latitude regions, reducing the consistency of burn‐severity products. Saberi and Harvey (2023) showed that short‐interval reburns can cause CBI and RdNBR to under‐ or overestimate severity depending on prior fire history. These findings indicate that post‐fire products require careful image selection, phenological matching, and consideration of previous disturbance history.

Smoke and air‐quality products have their own validation challenges because satellite observations often measure column‐integrated properties rather than surface exposure. AOD is widely used to monitor wildfire smoke, but it represents total‐column aerosol loading and does not directly measure surface PM_2.5_. Cheeseman et al. (2020) showed that the PM_2.5_‐to‐AOD relationship changes with smoke plume height relative to the planetary boundary layer. T. Zhao et al. (2024) showed that aerosol vertical distribution is a major uncertainty when converting satellite AOD to surface PM_2.5_ during Alaskan wildfire seasons. Huang, Ji, et al. (2024) further demonstrated that satellite plume‐height products can represent different plume‐height definitions, creating ambiguity for air‐quality and climate models. These studies show that AOD‐based smoke products must be interpreted with plume‐height and boundary‐layer information.

Satellite smoke retrievals are also affected by dense smoke, clouds, mixed aerosols, and land‐surface background. Ye et al. (2022) showed that updated Multi‐Angle Implementation of Atmospheric Correction (MAIAC) AOD retrievals improved performance during the 2020 western U.S. wildfire season, but retrieval quality still varied under thick smoke and near clouds. Daniels et al. (2024) found that MAIAC AOD agreed well with ground‐based AOD during medium and heavy smoke, but mixed smoke–dust aerosol and barren land introduced retrieval biases. Marey et al. (2024) showed that carbon monoxide retrievals from different satellite instruments agreed under normal conditions but diverged during intense wildfire smoke events with high aerosol loading. These studies demonstrate the need for quality filtering and product intercomparison during extreme smoke events.

Model‐based operational systems also remain constrained by emission uncertainty, plume‐rise representation, chemical mechanisms, and meteorological errors. Herron‐Thorpe et al. (2014) showed that regional air‐quality simulations underestimated wildfire PM_2.5_, AOD, nitrogen dioxide, and plume‐top height in the Pacific Northwest. Baker et al. (2018) found that the Community Multiscale Air Quality model captured Rim Fire plume transport but overestimated wildfire ozone and underestimated organic carbon near the fire. Li et al. (2025) showed that satellite AOD and ultraviolet aerosol index can be used to adjust wildfire emissions and improve PM_2.5_ forecasts. Dong et al. (2026) demonstrated that machine‐learning bias correction can reduce High‐Resolution Rapid Refresh Smoke PM_2.5_ underestimation, but also showed that missed smoke vertical structure and temperature inversions remain important sources of forecast error.

Validation uncertainty also affects health‐impact assessment. Geng et al. (2018) showed that satellite AOD, air‐quality modeling, and ground observations can improve high‐resolution fire‐season PM_2.5_ exposure estimates. Stowell et al. (2019) used wildfire‐specific PM_2.5_ exposure estimates to show significant associations with asthma and respiratory disease in Colorado. Johnson and Garcia‐Menendez (2022) showed that estimates of the health impacts of wildfire smoke are uncertain because they depend on both smoke PM_2.5_ exposure fields and epidemiological concentration–response parameters. These studies show that exposure uncertainty must be carried through to public‐health interpretation rather than treated as a separate technical issue.

Overall, validation and operational constraints remain central challenges for remote sensing‐based wildfire products. Fuel‐moisture retrievals require field calibration across vegetation types, active fire products must balance temporal and spatial resolution, burn‐severity products require local thresholds and careful image selection, and smoke products require vertical‐profile information to estimate surface exposure. Future operational systems should integrate multisensor satellite observations, field campaigns, ground monitoring networks, low‐cost sensors, LiDAR plume‐height products, chemical transport models, and uncertainty‐aware machine‐learning methods. Such integration will improve the reliability of wildfire products for early warning, fire management, post‐fire recovery planning, air‐quality forecasting, and public‐health decision‐making.

## 7 Strategies for Wildfire Mitigation and Resilient Design

Minimizing wildfire‐related threats is fundamental to national security and requires effective monitoring and management strategies (AghaKouchak et al., 2025). Key approaches include early detection and monitoring, fuel management and control burns, and fire‐resistant infrastructure (Bot & Borges, 2022; Chuvieco et al., 2023; Gonzalez‐Mathiesen et al., 2021; Miller & Ager, 2012; Moritz et al., 2014; Taylor et al., 2013; Thompson et al., 2013).

Early wildfire detection enables swift responses, reducing potential destruction. However, many wildfires occur in remote areas, creating significant monitoring and suppression challenges. Earlier detection can Technological advances have introduced several innovative remote detection and prediction methods. Satellite imagery has become a valuable tool for wildfire monitoring, with recent improvements in image resolution and satellite coverage allowing for faster and more accurate detection of fires. Advanced image processing techniques now enable not only heat detection but also the identification of burned areas using DL techniques (Hawkins et al., 2023; Rashkovetsky et al., 2021). UAVs or drones provide another effective solution for detecting, monitoring, and even suppressing wildfires. These drones excel at identifying heat sources that may invisible to naked eye, making them especially useful in hard‐to‐reach areas (Yuan et al., 2015). Additionally, predictive wildfire risk models offer early warning capabilities, helping to identify high risk areas and guide precautionary (Bot & Borges, 2022; Miller & Ager, 2012).

Fuel management is also critical for reducing wildfire risks. In recent years, fire management strategies have shifted from solely reducing wildfire risk to incorporating biodiversity objectives within increasing novel fire regimes, characterized by altered fire frequencies, intensities, and spatial patterns due to climate change and land‐use transformations (Puig‐Gironès et al., 2025). Using simulation models, Thompson et al. (2013) demonstrated that large‐scale fuel treatments can substantially reduce wildfire sizes and suppression costs over the long term. However, these findings depend on assumptions about the sustained effectiveness of treatments and the reliability of fire behavior and cost projections. In practice, agencies like the U.S. Forest Service aim to balance multiple objectives, restoring fire‐adapted ecosystems, minimizing risks to life and property, and prioritizing the safety of both firefighters and the public (Ingalsbee et al., 2015; Pietruszka et al., 2023). These competing goals often require nuanced decision‐making during wildfire events and may lead to alternative strategies beyond full suppression (Hai et al., 2023; Pietruszka et al., 2023).

Investing in fire‐resistant infrastructure is critical to enhance resilience against wildfires. The use of fireproof building materials and structural reinforcements can slow the spread of fires, facilitate control efforts, and mitigate potential damage. Modern wildfire protection regulations, such as California's building code Chapter 7A, Australia's Bushfire Attack Level system, and Portugal's new laws emphasize these elements to mitigate risks in WUI areas (Canosa et al., 2023; Cantor et al., 2023; Dossi et al., 2022; Ellery et al., 2023; Nikolakis & Roberts, 2022). While construction materials are important, factors such as defensible space, housing density, and location play a critical role in structure survival during wildfires (Syphard et al., 2017). The Physical Vulnerability Index identifies roof type, structure design, and nearby vegetation as key vulnerability indicators (Papathoma‐Köhle et al., 2022). Smith et al. (2025) highlight the value of local governance, noting that community‐led fire management is often more effective than top‐down approaches in wildfire‐prone areas.

To address the escalating complexity of wildfires as socio‐ecological crisis, Figure 11 illustrates the shift in wildfire governance is shifting from traditional suppression models toward a multi‐level, collaborative framework. This transition integrates a diverse array of stakeholders, ranging from government agencies and scientific institutions to private NGOs and local communities, to break down jurisdictional silos and enhance ecological resilience (Aldworth & Schultz, 2023; Kirschner et al., 2023; Miller et al., 2022). Within this framework, institutional and regulatory bodies provide the foundational support, with government agencies establishing funding and policy (Steelman, 2016), while local governments and urban planners manage the high‐risk WUI through strategic land‐use design (Schultz & Moseley, 2019). These formal structures are complemented by the financial and scientific sectors, where insurance companies incentivize risk reduction (Kousky & You, 2024) and researchers provide the climate projections and fire behavior models necessary for evidence‐based decision‐making (Cullen et al., 2023).

**Figure 11:**
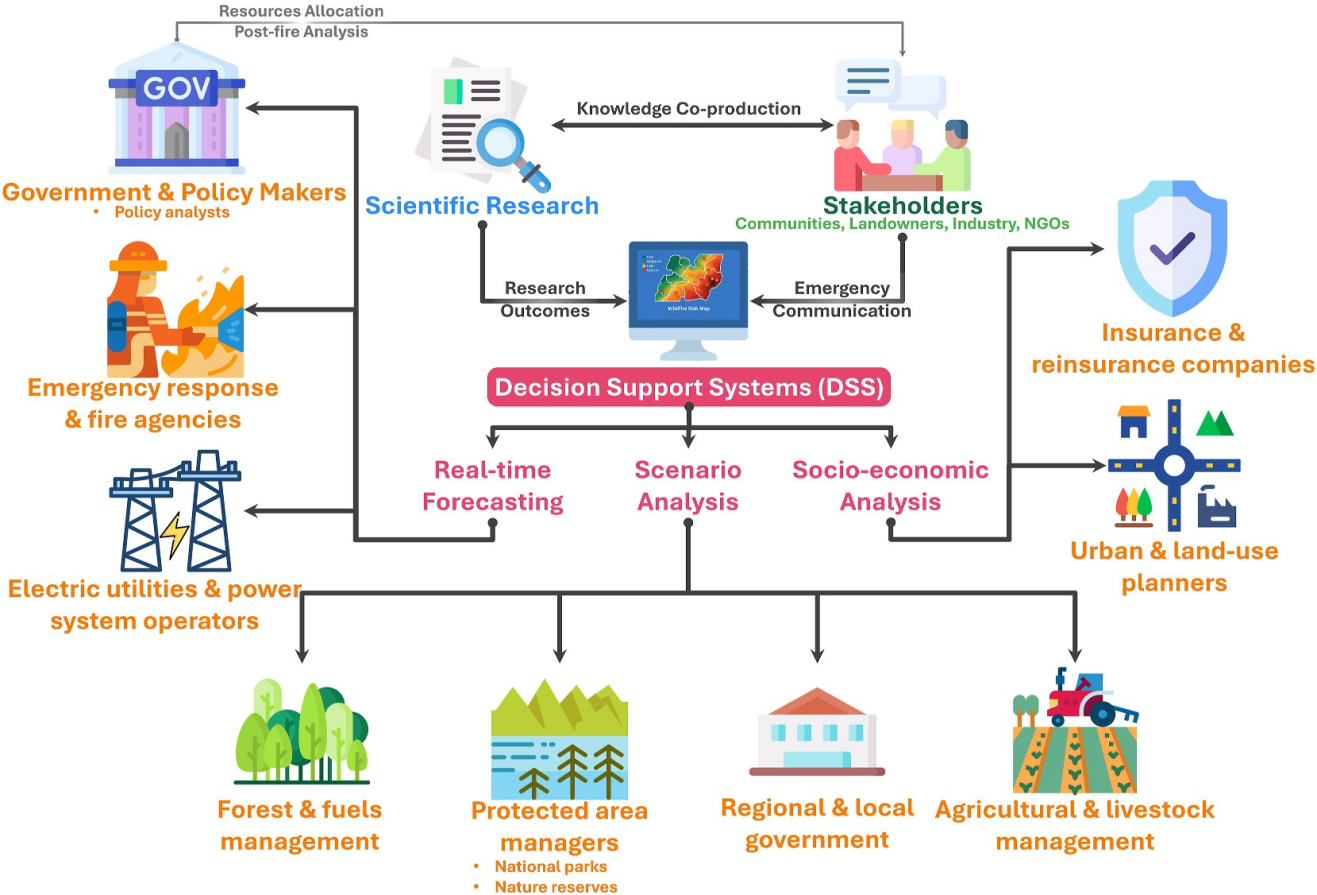
An integrated framework linking wildfire science, stakeholder engagement, and decision support systems for adaptive risk management. Scientific research co‐produced with stakeholders forms the foundation of the DSS. The DSS facilitates real‐time forecasting for immediate response by authorities and utilities, while scenario analysis supports long‐term planning for land and resource managers. Additionally, socio‐economic insights inform insurance strategies and urban land‐use planning.

On the operational front, land management and response actors execute the on‐the‐ground strategies essential for risk mitigation. Fire agencies manage immediate suppression efforts (Martell, 2015), while forest managers and agricultural stakeholders utilize nature‐based solutions, such as prescribed burns and mechanical thinning, to restore natural fire regimes and reduce fuel loads (A Mickler et al., 2013; Schultz & Moseley, 2019). This is further supported by electric utilities, which implement vegetation management and shutdown protocols to prevent infrastructure‐led ignitions (A Mickler et al., 2013; Abatzoglou et al., 2020; Castellon et al., 2024; Schultz & Moseley, 2019). Crucially, the DSS seves not only as a technological platform but as critical bridge that aligns scientific rigor (Section 5 and 6) with local and indigenous knowledge, ensuring that risk management strategies are culturally grounded and community‐respective (Hoffman et al., 2022; Jakes et al., 2007). However, despite these integrated efforts, governance remains hindered by institutional fragmentation, bureaucratic hurdles, and the displacement of local actors (Bacciu et al., 2025; Kirschner et al., 2024). Overcoming these gaps requires a co‐produced strategy that aligns institutional authority with local experience to mitigate community vulnerability (Essen et al., 2023). By fostering a co‐produced governance model, the DSS transforms raw data into a resilient, adaptive, and equitable framework for long‐term wildfire coexistence.

## 8 Current Challenges and Future Directions

Despite progress in identifying the key drivers of wildfire occurrence and spread, understanding the influence of climate and weather, and advancing forecasting technologies, critical knowledge gaps persist. Closing these gaps is essential to improve prediction, mitigation, and management strategies. This section presents the major challenges and emerging opportunities in wildfire research, highlighting future directions that can strengthen both scientific insight and practical response.

1. *Wildfire Behavior at the Human‐Environment Interface*: Research highlights that wildfire behavior arises from a complex, dynamic interplay among meteorological conditions, fuel characteristics, and human activities. While environmental drivers set the baseline flammability of landscapes and antecedent moisture controls regional fire potential, a critical challenge lies in addressing the growing influence of anthropogenic ignitions and Wildland‐Urban Interface (WUI) fuels. Unlike natural, seasonality‐bound fires, human‐driven ignitions increasingly contribute to economic and structural losses. Notably, research across the Western United States indicates that human‐started wildfires are responsible for approximately 76% if all structural losses (Higuera et al., 2023), particularly within the WUI. Additionally, research has indicated the impacts of the proximity of burnable materials to buildings, as well as the characteristics of the buildings themselves for example, in terms of roofing, siding and deck materials, in the destruction of structures and communities in wildfires (Zamanialaei et al., 2025) In general, the complex interactions of WUI fuel structures and wildfire propagation and behavior are not yet sufficiently resolved in physics‐based models, and WUI fire propagation data is limited to train ML models. This factors collectively underscore the urgent need to integrate human activity and land‐use patterns into wildfire prediction and management frameworks, moving beyond a purely biophysical perspective (O’Connor et al., 2016; Pourmohamad et al., 2026; Shuman et al., 2022). Future research must focus on quantifying these human‐environment interactions and developing strategies that reduce risk (e.g., fire prevention strategies, targeted fuel breaks) while enhancing landscape resilience.
2. *Machine Learning versus Physics‐based Models*: Any geoscientific model, physics‐based or data‐driven, conditions an n‐dimensional decision space of all relevant processes to predict a set of variables (Shojaeezadeh et al., 2018). Physics‐based models confine this n‐dimensional space through a set of regularizing relationships (e.g., ordinary/partial differential equations), also including a number of parameters that are tuned to fit observations. Such parameterizations are generally not scalable across domains (Khoshkalam et al., 2023; Xu & Liang, 2021). Furthermore, process representations in physics‐based models often require expert knowledge to modify, and may be associated with commission and omission errors, among other constraints (Clark et al., 2015). These factors limit the ability of physics‐based models to be transferable across diverse geographic areas and scales (Hrachowitz et al., 2013). Advanced ML models, however, do not regularize the n‐dimensional decision space a priori, and enable exploration of the entire space to find generalizable and transferable relationships between drivers and response (Nearing et al., 2021). Advanced ML models readily scale up with additional data and can learn both short‐term dynamics and decadal‐scale trends (Khoshkalam et al., 2023). They can learn regionally consistent and location‐specific forcing‐response relationships using “big data.” There is a notable opportunity to employ the “big data” ML approach to develop actionable and scalable wildfire models.
3. *Climate Change and Emerging Wildfire Extremes*: Climate change is reshaping wildfire regimes through shifts in temperature and precipitation, with fire behavior strongly influenced by regional vegetation and local climate characteristics (Bao et al., 2024; Sun et al., 2023). Recent megafires in Texas, Southern Europe, and Maui highlight that fire behavior increasingly exceeds historical precedents. A key challenge lies in the declining predictive utility of static, historical indices and measures of risk (e.g., burn probability, flame lengths). As climate change drives more anomalies, including those outside of the retrospective range of variability, traditional models struggle to capture the non‐linear interactions between rapid vegetation drying and extreme weather events. Predicting the “top 0.01%” of extreme fire events remains a significant barrier. Cunningham et al. (2024) conducted a global analysis indicating that the frequency and intensity of these most energetic wildfires have more than doubled over the past two decades, even as the total burned area remains stable or is declining in some regions. This presents a clear opportunity to replace static decision support systems with dynamic, ML‐powered frameworks. Future research must integrate real‐time live and dead fuel moisture dynamics derived from remote sensing with retrospective climate data to better anticipate extreme fire events.
4. *Subseasonal‐to‐Seasonal (S2S) Forecasts for Wildfires*: S2S forecasts provide lead times ranging from 2 weeks to several months, supporting proactive resource allocation, prescribed burning, and operational planning. These forecasting capability are largely underpinned by the strong teleconnections between global Sea Surface Temperature anomalies and regional burned areas (Chen et al., 2016; Turco et al., 2018). Despite these advances, forecast reliability decreases in regions where local terrain, vegetation, and fuel moisture differ markedly from large‐scale atmospheric patterns, limiting accuracy for extreme, low‐probability events. Future research should focus on addressing the decline in statistical predictive skill at extended leads and integrating real‐time meteorological data and high‐resolution environmental variables, as well as developing hybrid ML‐physics models that combine mechanistic fire behavior understanding with interpretable, actionable risk predictions.
5. *Decision Support Systems (DSS) for Wildfire Management*: DSS form the backbone of modern wildfire management by integrating high‐resolution remote sensing, real‐time environmental data, and community‐informed strategies to support detection, assessment, and mitigation. Geostationary satellites (e.g., GOES‐R) provide near‐instantaneous ignition detection, while polar‐orbiting platforms (e.g., Landsat, Sentinel‐2) enable multispectral burn severity mapping using advanced vegetation indices such as NIRv and EVI to assess ecosystem resilience. UAVs and ground‐based sensors enhance localized monitoring, though challenges remain in real‐time integration, spatial‐temporal trade‐offs, and deployment in remote, high‐risk terrains. DSS also bridges scientific modeling with WUI planning, fuel management, and community engagement, promoting resilience through early detection, defensible infrastructure, and adaptive governance. Future directions focus on advancing UAV swarm coordination, developing integrated multi‐platform sensor networks, deploying high‐resolution predictive tools, and strengthening socio‐economic resilience and inter‐agency collaboration to improve operational decision‐making under increasingly extreme fire regimes.

## 9 Conclusion

Wildfire research has undergone a fundamental transformation, evolving from a narrow focus on biophysical processes to a more holistic understanding of wildfire as a complex socio‐ecological challenge. This shift reflects the growing influence of intensifying climatic extremes and diverse anthropogenic pressures that collectively shape wildfire behavior, impacts, and management strategies. As a result, wildfires have become a major global threat, with far‐reaching consequences for ecosystems, air quality, public health, economies, and communities, often compounded by post‐fire hazards such as debris flows and water contamination. This review synthesizes current knowledge of the interconnected climatic, ecological, and human drivers of wildfire occurrence, emphasizing how climate change is amplifying risks through rising temperatures, altered precipitation patterns, prolonged drought, and more frequent extreme events.

Advances in remote sensing, satellite observations, unmanned aerial systems, and ML have significantly improved wildfire prediction, monitoring, and early warning capabilities. These technologies have enhanced the ability to detect ignitions, track fire behavior, and assess impacts across multiple spatial and temporal scales. However, critical challenges remain in real‐time monitoring, subseasonal‐to‐seasonal forecasting, data integration, and accessibility—particularly in remote and high‐risk regions. Addressing these limitations requires adaptive and integrated modeling frameworks that can assimilate diverse data streams while remaining responsive to rapidly evolving environmental conditions. In parallel, wildfire management has shifted from reactive, suppression‐based approaches toward more proactive strategies that emphasize integrated decision support systems (DSS), adaptive governance, and community‐centered resilience. This transition reflects a broader recognition that wildfire impacts extend well beyond immediate fire damage, encompassing cascading effects such as post‐fire flooding and debris flows, economic disruption, ecosystem degradation, public health risks, and disproportionate impacts on vulnerable populations. These interconnected challenges underscore the importance of holistic, cross‐sectoral approaches that support long‐term coexistence with wildfire.

Looking ahead, advancing predictive, transparent, and actionable DSS will be essential for bridging scientific innovation with socio‐economic resilience. Key priorities include improving the detection and prediction of rare, high‐impact extreme events, reducing uncertainties in subseasonal‐to‐seasonal forecasts, strengthening understanding of cascading wildfire risks, and more effectively integrating human–environment feedbacks—particularly within the WUI. Continued progress in DL, hybrid physics–machine learning approaches, and multi‐source remote sensing, including UAV and emerging satellite systems, offers strong potential to enhance predictive accuracy and early warning capabilities. In addition, adopting compound‐hazard frameworks can improve risk assessment by capturing interactions between wildfires and other climate extremes.

Overall, wildfire research now stands at a critical juncture, where advancing scientific capability must keep pace with escalating environmental and societal risks. Addressing the increasing complexity of wildfire regimes—driven by climate change and human activity—requires sustained interdisciplinary collaboration, improved data integration, and continued methodological innovation. By prioritizing actionable insights and bridging persistent knowledge gaps, future research can play a pivotal role in reducing risk, strengthening resilience, and enabling more sustainable coexistence with wildfire in an increasingly dynamic and uncertain world.

## Conflict of Interest

The authors declare no conflicts of interest relevant to this study.

## Supporting information

Supporting Information S1

## Data Availability

The wildfire occurrence data used in this study were obtained from the National Interagency Fire Center through the Integrated Reporting of Wildland–Fire Information (InFORM) database. The InFORM Fire Occurrence Data provide nationwide records of wildfire events across the United States, including ignition location, date, burned area, and cause of ignition. The data set is publicly available at https://data‐nifc.opendata.arcgis.com/datasets/nifc::inform‐fire‐occurrence‐data‐records/about.
