# Supplementary material for: Wildfire Drivers, Impacts, and Predictive Modeling: Opportunities in Sensing and Machine Learning
Source: Geohealth. 2026 Nov 11;10(11):e2025GH001734. doi: 10.1029/2025GH001734 (PMC13753081; doi:10.1029/2025GH001734)
Supplement: Supplementary file 1 [file GH2-10-e2025GH001734-s001.docx]

# Wildfire Drivers, Impacts, and Predictive Modeling: Opportunities in Sensing and Machine Learning

Jasmin Yujen Tung^1^; Ashok Mishra^1^; Mojtaba Sadegh^2,5^; Amitesh Sabut1; Amir Aghakouchak^3,4,5^; John Abatzoglou^6^; Naresh Devineni^7^; A. Sankarasubramanian^8^

^1^Zachry Department of Civil and Environmental Engineering, Texas A&M University, College Station, TX 77843, USA

^2^Department of Civil Engineering, Boise State University, Boise, ID, USA

^3^Department of Civil & Environmental Engineering, University of California, Irvine, CA, USA

^4^Department of Earth System Science, University of California, Irvine, CA, USA

^5^Institute for Water, Environment, and Health (UNU-INWEH), United Nations University, Richmond Hill, ON, L4B 3P4, Canada

^6^Department of Management of Complex Systems, University of California, Merced, Merced, CA, USA.

^7^Department of Civil Engineering, City University of New York (City College), New York, NY, USA

^8^Department of Civil, Construction, and Environmental Engineering, North Carolina State University, USA

Corresponding Author: Ashok Mishra ([ashok_mishra@tamu.edu](mailto:ashok_mishra@tamu.edu))

This PDF file Includes:

Text S1

Table S1 to S3

**Text S1**

### **S1.1 Support Vector Machine (SVM)**

Support Vector Machines (SVM) are supervised learning algorithms widely applied in wildfire prediction tasks, particularly for classification problems such as predicting fire occurrence, fire severity, or fuel type (Jain et al., 2020). SVM operates by identifying an optimal hyperplane that maximizes the margin between different classes, enabling accurate separation of data points (Mehra & Gupta, 2015). With kernel functions, SVM can effectively handle non-linear relationships by projecting data into higher-dimensional feature spaces (Mittal et al., 2024). For example, in the Guangxi Autonomous Region of China, SVM predicted forest fires with an accuracy of 89.89% based on satellite monitoring data and environmental factors, meeting the required standards for effective prediction despite being slightly outperformed by neural networks (Li et al., 2020). In California, an SVM model using remote sensing data and fire incident records achieved a county-level prediction accuracy of 96%, demonstrating its strong performance in regional wildfire prediction (Pham et al., 2022).

### **S1.2 Random Forest**

Random Forest (RF), an ensemble learning method, is highly effective for both classification and regression tasks (Dudek, 2022). The model operates by constructing multiple decision trees using randomly selected subsets of data and features. Each tree makes independent predictions, which are then aggregated either through majority voting for classification tasks or averaging for regression tasks, resulting in improved accuracy and reduced overfitting.

In wildfire prediction, Random Forests have demonstrated strong predictive capabilities across diverse applications. For example, in wildfire susceptibility mapping in Irkutsk Oblast, Russia, RF achieved an accuracy of 89% and an AUC of 0.96 by incorporating meteorological, topographic, and vegetation variables (Nikolaychuk et al., 2024). For wildfire occurrence prediction, Thakkar et al. (2022) applied RF in Portugal’s Montesinho Natural Park, yielding a correlation matrix with a mean prediction error of 0.66. Moreover, Makowski (2023) reported a 9% improvement in predicting fire frequency and burned area compared to previous models using a simple RF framework, highlighting its operational value for decision-makers.

One of the key strengths of Random Forests is their ability to handle continuous variables and capture nonlinear relationships between predictors, making them particularly useful for wildfire prediction. However, it is crucial to compare their performance with alternative models and carefully tune parameters to ensure optimal results, as they may not always offer the best fit for every scenario (Cheng et al., 2022; Dong et al., 2022; Kondylatos et al., 2022; Malik et al., 2021). By leveraging their robust performance and adaptability, Random Forests continue to be a valuable tool in wildfire prediction research and applications.

### **S1.3 XGBoost**

XGBoost (eXtreme Gradient Boosting) is a powerful and scalable tree boosting system that has become widely adopted in the machine learning community due to its high efficiency and predictive performance (Chen & Guestrin, 2016). XGBoost builds a series of decision trees, where each corrects the errors of the previous one by minimizing a loss function using gradient descent. This method allows XGBoost to capture complex patterns in the data while mitigating overfitting.

In the context of wildfire research, XGBoost has shown strong performance in various applications. For instance, in wildfire susceptibility mapping in Turkey’s Antalya province, XGBoost achieved an accuracy of 85.4%, outperforming alternative models such as Random Forest and AdaBoost (Bilucan et al., 2022). Similarly, Abujayyab et al. (2022) applied XGBoost to the Mediterranean region and obtained an accuracy of 88.8% in predicting wildfire-prone areas. These models incorporated environmental, topographic, and vegetation-related variables, and their superior performance demonstrates XGBoost’s capability to model spatial wildfire risk with high precision.

Beyond mapping, XGBoost also contributes to strategic wildfire management. Dong et al. (2022) highlighted that the algorithm’s high predictive accuracy allows stakeholders to develop targeted mitigation strategies, which may reduce the ecological and health-related impacts of large-scale fires.

### **S1.4 Convolutional Neural Networks (CNN)**

Convolutional Neural Networks (CNN) are particularly effective for analyzing gridded data, such as satellite imagery, making them valuable tools in wildfire predictions and monitoring. Specialized architectures, such as ConvLSTM, build upon CNN to extract features from complex imagery, enabling wildfire risk prediction, fire propagation modeling, and severity classification. A key advantage of CNN-based models is their ability to incorporate multi-type data, including satellite images and environmental variables. CNN comprises multiple layers, including convolutional layers for feature extraction, pooling layers for dimensionality reduction, and fully connected layers for classification or regression tasks. However, they require substantial training data, which may not always be available for specific study areas or scenarios (Deng et al., 2023; Kanwal et al., 2023; Yu et al., 2024).

CNNs have shown promising results in various wildfire-related applications. For instance, a CNN model developed using Sentinel-2 imagery achieved an accuracy of 92.5% in wildfire detection tasks (Abujayyab et al., 2023). In the Amazon rainforest, CNNs were successfully applied to classify fire-affected regions using Landsat imagery (Eleutério et al., 2025), while in Australia, Himawari-8 satellite data enabled CNN models to detect wildfire locations and intensities with over 80% accuracy (Ding et al., 2022). Additionally, Mythili et al. (2023) employed CNNs for real-time wildfire occurrence prediction by analyzing historical weather and fire occurrence data, improving emergency response planning.

### **S1.5 Long Short-Term Memory (LSTM)**

Long Short-Term Memory (LSTM) is a specialized type of recurrent neural network (RNN) architecture designed to process and learn from sequential data (Okut, 2021; Ravikumar & Sriraman, 2024). Unlike traditional RNNs, which often face challenges with long-term dependencies due to vanishing or exploding gradients, LSTMs are designed to maintain information over extended sequences. This capability makes them especially well-suited for tasks involving time-series analysis and sequential pattern recognition.

LSTMs are composed of memory cells, that serve as the core building blocks for information processing. Each memory cell includes three types of gates (Okut, 2021): (a) Forget Gate: Determines which information from the previous state should be discarded, (b) Input Gate: Decides what new information should be stored in the memory cell, (c) Output Gate: Regulates the amount of the cell’s stored information that is utilized at the current time step or forwarded to the next layer. This gated mechanism allows LSTMs to selectively retain or forget information, enabling them to effectively capture long-term dependencies in sequential data.

In wildfire modeling, LSTMs are widely used to analyze temporal datasets, such as historical climate variables, vegetation indices, and fire events. For example, Li et al. (2021) developed the Multi-AM-LSTM model, which integrated an attention mechanism and achieved over 96% accuracy in predicting burned areas based on meteorological and fire history data. In Thailand’s Mae Hong Son region, LSTM models demonstrated strong performance in estimating wildfire-affected areas using climatic inputs (Sawetsuthipan & Wongchaisuwat, 2023). Similarly, LSTM networks trained on satellite-derived time-series data achieved near-real-time wildfire detection with high accuracy, outperforming traditional methods (Zhang et al., 2022).

LSTM has also proven useful in mapping wildfire susceptibility. Bahadori et al. (2023) applied LSTM with remote sensing inputs to generate risk maps that identify high-vulnerability zones. Furthermore, many LSTM applications in wildfire prediction integrate convolutional neural networks (CNNs) to leverage both spatial and temporal data (Marjani et al., 2024; Mirones et al., 2025). Hybrid models, such as SLA-ConvLSTM, ConvLSTM, and 3D CNN-ConvLSTM, combine the spatial feature extraction capabilities of CNNs with the sequential learning power of LSTMs, enhancing accuracy in risk map generation (Bhowmik et al., 2023; Deng et al., 2023; Ji et al., 2024; Kondylatos et al., 2022).

Table S 1. Summary of the 30 representative wildfire risk modeling studies

| **#** | **Year & (ref)** | **Variables** | **Study Area** |
| --- | --- | --- | --- |
| 1 | 2024  (Sivrikaya et al., 2024) | NBR NBRT NDVI NDWI | Türkiye |
| 2 | 2025  (Sivrikaya & Demirel, 2025) | Tree species NDVI Forest cover Slope Aspect Topographic Wetness Index Distance from settlement and agriculture areas Distance from road Distance from river Population density Precipitation Wind speed Solar radiation Annual mean temperature Mean diurnal range Isothermality Temperature seasonality Precipitation of wettest month Precipitation of driest month Precipitation seasonality | Türkiye |
| 3 | 2021  (Zhao et al., 2021) | Altitude Slope Aspect TWI Temperature NDVI Distance to Road Distance to Populated Areas | Nanjing, China |
| 4 | 2021  (Michael et al., 2021) | Altitude Slope Aspect NDVI NDWI | Greece |
| 5 | 2020  (Ziccardi et al., 2020) | T RH Wind Speed P Dew point Historical Wildfire | Brazil |
| 6 | 2020  (Parajuli et al., 2020) | Aspect Slope Elevation Land cover Land Surface Temperature Distance to Road Proximity to Settlement Fire Incidents Burned Area | Nepal |
| 7 | 2020  (Kumari & Pandey, 2020) | Fuel Type Distance from Settlement Distance from Road Bare Soil Index, BI Aspect Elevation Slope | India |
| 8 | 2023  (Cetin et al., 2023) | Stand Characteristics Slope Aspect Elevation Distance from Intermittent Streams Distance from Roads Distance from Settlements | Türkiye |
| 9 | 2023  (Shi & Zhang, 2023) | Slope Aspect TWI Altitude Distance to Roads Distance to Population Centers NDVI Mean Monthly Temperature | China |
| 10 | 2024  (Dhakal et al., 2024) | Historical fire Slope Aspects Topographic Wetness Index, TWI Elevation Land Cover NDVI Land Surface Temperature, LST Wind speed wind direction P Distance from Road Proximity to Settlement | Nepal |
| 11 | 2020  (Çolak & Sunar, 2020) | Fuel Type LST Canopy closure Slope Aspect Proximity to Settlement and Road | Türkiye |
| 12 | 2020  (Bürger et al., 2020) | wind speed wind direction fuel T | NA |
| 13 | 2025  (Wang et al., 2025) | Daily Temperature Dew Point Temperature Average Air Pressure Average Wind Speed Precipitation Altitude TWI NDVI Slope Distance to Industrial Areas Distance to Road Distance to River Distance to Residential Areas Population Density | Anhui, China |
| 14 | 2023  (Shafapourtehrany, 2023) | Altitude Slope Aspect TWI, Topographic Wetness Index NDVI Distance to Road Distance to River Precipitation Wind Speed Forest Types LULC Geology Children and Elderly Population Density Residential Building Density Commercial Building Density Universities, Colleges, and Schools Emergency Services Facilities Populated Places Power Generation Facilities Protected Areas Tourist Points | Australia |
| 15 | 2023  (Feizizadeh et al., 2023) | Elevation Slope Aspect LULC NDVI Distance to burnt sites Wind speed Wind direction Land Surface Temperature, LST Soil moisture Precipitation Distance to road Distance to residences | Iran |
| 16 | 2025  (Sapkota et al., 2025) | Longitude Latitude Altitude Slope Aspect Precipitation Air Temperature Dewpoint Temperature LST Soil Moisture Wind Speed VPD Relative Humidity, RH Sunlight Hours Distance to Nearest Highway NDVI | Nepal |
| 17 | 2023  (Tan & Feng, 2023) | Temperature Precipitation Pressure RH sunlight Wind speed altitude slope aspect NDVI distance to resident distance to highway distance to railroad population density GDP holiday longitude latitude | China |
| 18 | 2021  (Juvanhol et al., 2021) | LCLU Proximity to Roads Slope Relief Orientation Proximity to Residences | Brazil |
| 19 | 2022  (Santos et al., 2022) | LCLU DEM population density road network Viewshed historical wildfire NDVI S2-NDVI | Portugal |
| 20 | 2022  (Mohammed et al., 2022) | Altitude Slope Percentage Slope Direction/Aspect Distance from Roads Distance from Rivers Distance from Settlements Land Use Slope Curvature Average Annual Rainfall Maximum Annual Temperature | Iran |
| 21 | 2022  (Maniatis et al., 2022) | Land Cover, LC Altitude Aspect Slope Topographic Wetness Index, TWI Distance from Roads Distance from Settlements | Greece |
| 22 | 2020  (Pourghasemi et al., 2020) | Slope angle Altitude Aspect Plan curvature Profile curvature TWI Geology LULC Distance to rivers Distance to faults Distance to roads Mean annual temperature Mean annual rainfall Distance to residential areas Soil features Drainage density | Iran |
| 23 | 2023  (Heo et al., 2023) | Temperature Wind speeds Rainfall Distance to roads Distance to river Population Aspect Elevation Slope TWI Land cover historical drought historical forest fires | Indonesia |
| 24 | 2022  (Casallas et al., 2022) | FWI NDVI NDWI LC | Colombia |
| 25 | 2024  (Peng et al., 2024) | T RH Wind Speed P Drought factors Slope Aspect distance from road historical forest fire | China |
| 26 | 2023  (Xu et al., 2023) | temperature wind speed RH precipitation vegetation type population density distance from road distance from water distance from settlement | China |
| 27 | 2020  (Coban & ERDİN, 2020) | Proximity to settlements Proximity to the road network Proximity to the cultivated areas Proximity to the power lines Vegetation type Age of stand development Crown closure Altitude Slope Aspect temperature precipitation wind speed | Türkiye |
| 28 | 2024  (Fernández-Manso et al., 2024) | topography climate weather fuel | Spain |
| 29 | 2020  (Zhang et al., 2020) | Altitude Slope Aspect TWI, Topographic Wetness Index temperature NDVI distance to populated areas distance to roads | China |
| 30 | 2020  (Novo et al., 2020) | Elevation Slope Aspect NDVI Fuel model type FWI Road Distance Settlement Distance Historical Fire Regimes | Spain |

Table S 2. Frequency of use of the variables listed in Table S1

| **Paper #** | **1** | **2** | **3** | **4** | **5** | **6** | **7** | **8** | **9** | **10** | **11** | **12** | **13** | **14** | **15** | **16** | **17** | **18** | **19** | **20** | **21** | **22** | **23** | **24** | **25** | **26** | **27** | **28** | **29** | **30** | **sum** |
| --- | --- | --- | --- | --- | --- | --- | --- | --- | --- | --- | --- | --- | --- | --- | --- | --- | --- | --- | --- | --- | --- | --- | --- | --- | --- | --- | --- | --- | --- | --- | --- |
| **slope** |  | 1 | 1 | 1 |  | 1 | 1 | 1 | 1 | 1 | 1 |  | 1 | 1 | 1 | 1 | 1 | 1 |  | 1 | 1 | 1 | 1 |  | 1 |  | 1 |  | 1 | 1 | 23 |
| **distance from a road** |  | 1 |  |  |  | 1 |  | 1 | 1 | 1 | 1 |  | 1 | 1 | 1 | 1 | 1 | 1 | 1 | 1 | 1 | 1 | 1 |  | 1 | 1 | 1 |  | 1 | 1 | 22 |
| **altitude** |  |  | 1 | 1 |  | 1 | 1 | 1 | 1 | 1 |  |  | 1 | 1 | 1 | 1 | 1 |  | 1 | 1 | 1 | 1 | 1 |  |  |  | 1 | 1 | 1 | 1 | 21 |
| **aspect** |  | 1 | 1 | 1 |  | 1 | 1 | 1 | 1 | 1 | 1 |  |  | 1 | 1 | 1 | 1 |  |  | 1 | 1 | 1 | 1 |  | 1 |  | 1 |  | 1 | 1 | 21 |
| **temperature** |  | 1 | 1 |  | 1 | 1 |  |  | 1 | 1 | 1 | 1 | 1 |  | 1 | 1 | 1 |  |  | 1 |  | 1 | 1 |  | 1 | 1 | 1 | 1 | 1 |  | 20 |
| **distance from a village** |  | 1 | 1 |  |  | 1 | 1 | 1 | 1 | 1 | 1 | 1 | 1 |  | 1 |  | 1 | 1 |  | 1 | 1 | 1 |  |  |  | 1 | 1 |  | 1 | 1 | 20 |
| **precipitation** |  | 1 |  |  | 1 |  |  |  |  | 1 |  |  | 1 | 1 | 1 | 1 | 1 |  |  | 1 |  | 1 | 1 |  | 1 | 1 | 1 | 1 |  |  | 15 |
| **NDVI** | 1 | 1 | 1 | 1 |  |  |  |  | 1 | 1 |  |  | 1 | 1 | 1 | 1 | 1 |  | 1 |  |  |  |  | 1 |  |  |  |  | 1 | 1 | 15 |
| **wind speed** |  | 1 |  |  | 1 |  |  |  |  | 1 |  | 1 | 1 | 1 | 1 | 1 | 1 |  |  |  |  |  | 1 |  | 1 | 1 | 1 |  |  |  | 13 |
| **land cover** |  |  |  |  |  | 1 |  |  |  | 1 |  |  |  | 1 | 1 |  |  | 1 | 1 | 1 | 1 | 1 | 1 |  |  |  |  |  |  |  | 10 |
| **distance from a river** |  | 1 | 1 |  |  |  | 1 | 1 |  |  |  |  | 1 | 1 |  |  |  |  |  | 1 |  | 1 | 1 |  |  | 1 |  |  |  |  | 10 |
| **TWI** |  | 1 | 1 |  |  |  |  |  | 1 | 1 |  |  | 1 | 1 |  |  |  |  |  |  | 1 | 1 | 1 |  |  |  |  |  | 1 |  | 10 |
| **historical wildfire records** |  |  |  |  | 1 | 1 |  |  |  | 1 |  |  |  |  | 1 |  |  |  | 1 |  |  |  | 1 |  | 1 |  |  |  |  | 1 | 8 |
| **vegetation type** |  | 1 |  |  |  |  | 1 |  |  |  |  | 1 |  | 1 |  |  |  |  |  |  |  |  |  |  |  | 1 | 1 | 1 |  | 1 | 8 |
| **population density** |  | 1 |  |  |  |  |  |  |  |  |  |  | 1 |  |  |  | 1 |  | 1 |  |  |  | 1 |  |  | 1 |  |  |  |  | 6 |
| **relative humidity** |  |  |  |  | 1 |  |  |  |  |  |  |  |  |  |  | 1 | 1 |  |  |  |  |  |  |  | 1 | 1 |  |  |  |  | 5 |
| **dew point** |  |  |  |  | 1 |  |  |  |  |  |  |  | 1 |  |  | 1 |  |  |  |  |  |  |  |  |  |  |  |  |  |  | 3 |
| **wind direction** |  |  |  |  |  |  |  |  |  | 1 |  | 1 |  |  | 1 |  |  |  |  |  |  |  |  |  |  |  |  |  |  |  | 3 |
| **NDWI** | 1 |  |  | 1 |  |  |  |  |  |  |  |  |  |  |  |  |  |  |  |  |  |  |  | 1 |  |  |  |  |  |  | 3 |
| **sunlight hour** |  |  |  |  |  |  |  |  |  |  |  |  |  |  |  | 1 | 1 |  |  |  |  | 1 |  |  |  |  |  |  |  |  | 3 |
| **soil moisture** |  |  |  |  |  |  |  |  |  |  |  |  |  |  | 1 | 1 |  |  |  |  |  |  |  |  |  |  |  |  |  |  | 2 |
| **surface pressure** |  |  |  |  |  |  |  |  |  |  |  |  | 1 |  |  |  | 1 |  |  |  |  |  |  |  |  |  |  |  |  |  | 2 |
| **latitude** |  |  |  |  |  |  |  |  |  |  |  |  |  |  |  | 1 | 1 |  |  |  |  |  |  |  |  |  |  |  |  |  | 2 |
| **longitude** |  |  |  |  |  |  |  |  |  |  |  |  |  |  |  | 1 | 1 |  |  |  |  |  |  |  |  |  |  |  |  |  | 2 |
| **FWI** |  |  |  |  |  |  |  |  |  |  |  |  |  |  |  |  |  |  |  |  |  |  |  | 1 |  |  |  |  |  | 1 | 2 |
| **land use** |  |  |  |  |  |  |  |  |  |  |  |  |  |  |  |  |  |  |  |  |  |  |  | 1 |  |  |  |  |  |  | 1 |
| **vapor pressure deficit** |  |  |  |  |  |  |  |  |  |  |  |  |  |  |  | 1 |  |  |  |  |  |  |  |  |  |  |  |  |  |  | 1 |
| **surface solar radiation downwards** |  | 1 |  |  |  |  |  |  |  |  |  |  |  |  |  |  |  |  |  |  |  |  |  |  |  |  |  |  |  |  | 1 |
| **powerline** |  |  |  |  |  |  |  |  |  |  |  |  |  |  |  |  |  |  |  |  |  |  |  |  |  |  | 1 |  |  |  | 1 |
| **NBR** | 1 |  |  |  |  |  |  |  |  |  |  |  |  |  |  |  |  |  |  |  |  |  |  |  |  |  |  |  |  |  | 1 |
| **Bare soil index (BI)** |  |  |  |  |  |  | 1 |  |  |  |  |  |  |  |  |  |  |  |  |  |  |  |  |  |  |  |  |  |  |  | 1 |
| **geology** |  |  |  |  |  |  |  |  |  |  |  |  |  | 1 |  |  |  |  |  |  |  |  |  |  |  |  |  |  |  |  | 1 |
| **GDP** |  |  |  |  |  |  |  |  |  |  |  |  |  |  |  |  | 1 |  |  |  |  |  |  |  |  |  |  |  |  |  | 1 |
| **drainage density** |  |  |  |  |  |  |  |  |  |  |  |  |  |  |  |  |  |  |  |  |  | 1 |  |  |  |  |  |  |  |  | 1 |

Table S 3. Machine Learning Models for Wildfire Applications

| **Model** | **Task Type** | **Wildfire Applications** | **Reference** |
| --- | --- | --- | --- |
| Support Vector Machine  (SVM / SVR) | Classification & Regression | Fire occurrence modeling,  burned area estimation | (Jaafari & Pourghasemi, 2019; Pereira et al., 2017) |
| Random Forest | Classification & Regression | Fire risk zoning,  burned area estimation | (Chen et al., 2025; Shimabukuro et al., 2023) |
| Decision Tree | Classification | Fire susceptibility mapping | (Pham et al., 2020) |
| Gradient Boosting  (XGBoost, LightGBM, CatBoost) | Classification & Regression | Fire risk prediction,  Burn severity modeling | (Huang et al., 2024; Pourmohamad et al., 2026; Vanderhoof et al., 2025) |
| Artificial Neural Network  (ANN) | Classification & Regression | Fire occurrence modeling,  burned area estimation | (de Bem et al., 2019; Pérez-Sánchez et al., 2019) |
| Convolutional Neural Network  (CNN) | Classification | Fire detection  (from satellite images) | (Goel et al., 2023) |
| Recurrent Neural Network  (RNN / LSTM) | Regression | Temporal fire spread & behavior | (Masrur et al., 2024) |
| Logistic Regression | Classification | Fire occurrence modeling,  ignition probability | (de Bem et al., 2019) |
| Linear Regression | Regression | Burned area estimation | (Marsha & Larkin, 2022; Moreno Ruiz et al., 2014) |

# References

Abujayyab, S. K., Karas, I. R., Hashempour, J., Emircan, E., Orçun, K., & Ahmet, G. (2023). Wildfire detection from sentinel imagery using convolutional neural network (cnn). The Proceedings of the International Conference on Smart City Applications,

Abujayyab, S. K., Kassem, M. M., Khan, A. A., Wazirali, R., Coşkun, M., Taşoğlu, E., Öztürk, A., & Toprak, F. (2022). Wildfire susceptibility mapping using five boosting machine learning algorithms: the case study of the mediterranean region of Turkey. *Advances in Civil Engineering*, *2022*(1), 3959150.

Bahadori, N., Razavi-Termeh, S. V., Sadeghi-Niaraki, A., Al-Kindi, K. M., Abuhmed, T., Nazeri, B., & Choi, S.-M. (2023). Wildfire susceptibility mapping using deep learning algorithms in two satellite imagery dataset. *Forests*, *14*(7), 1325.

Bhowmik, R. T., Jung, Y. S., Aguilera, J. A., Prunicki, M., & Nadeau, K. (2023). A multi-modal wildfire prediction and early-warning system based on a novel machine learning framework. *Journal of Environmental Management*, *341*, 117908.

Bilucan, F., Teke, A., & Kavzoglu, T. (2022). Susceptibility mapping of wildfires using xgboost, random forest and adaboost: A case study of mediterranean ecosystem. international conference on Mediterranean Geosciences Union,

Bürger, R., Gavilán, E., Inzunza, D., Mulet, P., & Villada, L. M. (2020). Exploring a convection–diffusion–reaction model of the propagation of forest fires: Computation of risk maps for heterogeneous environments. *Mathematics*, *8*(10), 1674.

Casallas, A., Jiménez-Saenz, C., Torres, V., Quirama-Aguilar, M., Lizcano, A., Lopez-Barrera, E. A., Ferro, C., Celis, N., & Arenas, R. (2022). Design of a forest fire early alert system through a deep 3D-CNN structure and a WRF-CNN bias correction. *Sensors*, *22*(22), 8790.

Cetin, M., Isik Pekkan, Ö., Ozenen Kavlak, M., Atmaca, I., Nasery, S., Derakhshandeh, M., & Cabuk, S. N. (2023). GIS-based forest fire risk determination for Milas district, Turkey. *Natural Hazards*, *119*(3), 2299-2320.

Chen, D., Zeng, A., He, Y., Ouyang, Y., Li, C., Tigabu, M., Wang, W., Ni, R., Zhang, J., & Guo, F. (2025). Study on Small-Scale Forest Fire Risk Zoning Based on Random Forest and the Fuzzy Analytic Network Process. *Forests*, *16*(1), 97.

Chen, T., & Guestrin, C. (2016). Xgboost: A scalable tree boosting system. Proceedings of the 22nd acm sigkdd international conference on knowledge discovery and data mining,

Cheng, S., Jin, Y., Harrison, S. P., Quilodrán-Casas, C., Prentice, I. C., Guo, Y.-K., & Arcucci, R. (2022). Parameter flexible wildfire prediction using machine learning techniques: Forward and inverse modelling. *Remote Sensing*, *14*(13), 3228.

Coban, H., & ERDİN, C. (2020). Forest fire risk assessment using GIS and AHP integration in Bucak forest enterprise, Turkey. *Applied Ecology and Environmental Research*, *18*(1).

Çolak, E., & Sunar, F. (2020). Evaluation of forest fire risk in the Mediterranean Turkish forests: A case study of Menderes region, Izmir. *International journal of disaster risk reduction*, *45*, 101479.

de Bem, P. P., de Carvalho Júnior, O. A., Matricardi, E. A. T., Guimarães, R. F., & Gomes, R. A. T. (2019). Predicting wildfire vulnerability using logistic regression and artificial neural networks: a case study in Brazil’s Federal District. *International Journal of Wildland Fire*, *28*(1), 35-45.

Deng, J., Wang, W., Gu, G., Chen, Z., Liu, J., Xie, G., Weng, S., Ding, L., & Li, C. (2023). Wildfire susceptibility prediction using a multisource and spatiotemporal cooperative approach. *Earth Science Informatics*, *16*(4), 3511-3529.

Dhakal, M., Bhatta, B., Lamichhane, P., & Parajuli, A. (2024). Synergistic approaches in forest fire risk mapping using fuzzy AHP and machine learning models in the Chure Tarai Madhesh Landscape (CTML) of Nepal. *Geomatics, Natural Hazards and Risk*, *15*(1), 2436540.

Ding, C., Zhang, X., Chen, J., Ma, S., Lu, Y., & Han, W. (2022). Wildfire detection through deep learning based on Himawari-8 satellites platform. *International journal of remote sensing*, *43*(13), 5040-5058.

Dong, H., Wu, H., Sun, P., & Ding, Y. (2022). Wildfire prediction model based on spatial and temporal characteristics: A case study of a wildfire in Portugal’s Montesinho Natural Park. *Sustainability*, *14*(16), 10107.

Dudek, G. (2022). A comprehensive study of random forest for short-term load forecasting. *Energies*, *15*(20), 7547.

Eleutério, C. L., Filizola, N. P., de Brito, A. P., Galiceanu, M., & Mendes, C. F. (2025). Identifying wildfires with convolutional neural networks and remote sensing: application to Amazon Rainforest. *International journal of remote sensing*, *46*(7), 2665-2688.

Feizizadeh, B., Omarzadeh, D., Mohammadnejad, V., Khallaghi, H., Sharifi, A., & Karkarg, B. G. (2023). An integrated approach of artificial intelligence and geoinformation techniques applied to forest fire risk modeling in Gachsaran, Iran. *Journal of Environmental Planning and Management*, *66*(6), 1369-1391.

Fernández-Manso, A., Quintano, C., Fernández-Guisuraga, J. M., & Roberts, D. (2024). Next-gen regional fire risk mapping: Integrating hyperspectral imagery and National Forest Inventory data to identify hot-spot wildland-urban interfaces. *Science of The Total Environment*, *940*, 173568.

Goel, A., Bhende, M., Lal, M., Pathak, A., Tamrakar, P., Thorat, P., & Sharma, S. (2023). An emerging fire detection system based on convolutional neural network and aerial-based forest fire identification. 2023 IEEE International Conference on Computer Vision and Machine Intelligence (CVMI),

Heo, S., Park, S., & Lee, D. K. (2023). Multi-hazard exposure mapping under climate crisis using random forest algorithm for the Kalimantan Islands, Indonesia. *Scientific Reports*, *13*(1), 13472.

Huang, S., Ji, J., Wang, Y., Li, W., & Zheng, Y. (2024). Development and validation of a soft voting-based model for urban fire risk prediction. *International journal of disaster risk reduction*, *101*, 104224.

Jaafari, A., & Pourghasemi, H. R. (2019). Factors influencing regional-scale wildfire probability in Iran: an application of random forest and support vector machine. In *Spatial modeling in GIS and R for Earth and environmental sciences* (pp. 607-619). Elsevier.

Jain, P., Coogan, S. C., Subramanian, S. G., Crowley, M., Taylor, S., & Flannigan, M. D. (2020). A review of machine learning applications in wildfire science and management. *Environmental Reviews*, *28*(4), 478-505.

Ji, Y., Wang, D., Li, Q., Liu, T., & Bai, Y. (2024). Global Wildfire Danger Predictions Based on Deep Learning Taking into Account Static and Dynamic Variables. *Forests*, *15*(1), 216.

Juvanhol, R. S., Fiedler, N. C., Santos, A. R. D., Silva, G. F. D., Omena, M. S., Eugenio, F. C., Pinheiro, C. J. G., & Ferraz Filho, A. C. (2021). Gis and fuzzy logic applied to modelling forest fire risk. *Anais da Academia Brasileira de Ciências*, *93*(suppl 3), e20190726.

Kanwal, R., Rafaqat, W., Iqbal, M., & Weiguo, S. (2023). Data-Driven Approaches for Wildfire Mapping and Prediction Assessment Using a Convolutional Neural Network (CNN). *Remote Sensing*, *15*(21), 5099.

Kondylatos, S., Prapas, I., Ronco, M., Papoutsis, I., Camps‐Valls, G., Piles, M., Fernández‐Torres, M. Á., & Carvalhais, N. (2022). Wildfire danger prediction and understanding with deep learning. *Geophysical Research Letters*, *49*(17), e2022GL099368.

Kumari, B., & Pandey, A. C. (2020). Geo-informatics based multi-criteria decision analysis (MCDA) through analytic hierarchy process (AHP) for forest fire risk mapping in Palamau Tiger Reserve, Jharkhand state, India. *Journal of Earth System Science*, *129*(1), 204.

Li, Y., Feng, Z., Chen, S., Zhao, Z., & Wang, F. (2020). Application of the artificial neural network and support vector machines in forest fire prediction in the guangxi autonomous region, China. *Discrete Dynamics in Nature and Society*, *2020*(1), 5612650.

Li, Z., Huang, Y., Li, X., & Xu, L. (2021). Wildland fire burned areas prediction using long short-term memory neural network with attention mechanism. *Fire technology*, *57*(6), 1-23.

Makowski, D. (2023). Simple random forest classification algorithms for predicting occurrences and sizes of wildfires. *Extremes*, *26*(2), 331-338.

Malik, A., Rao, M. R., Puppala, N., Koouri, P., Thota, V. A. K., Liu, Q., Chiao, S., & Gao, J. (2021). Data-driven wildfire risk prediction in northern California. *Atmosphere*, *12*(1), 109.

Maniatis, Y., Doganis, A., & Chatzigeorgiadis, M. (2022). Fire risk probability mapping using machine learning tools and multi-criteria decision analysis in the gis environment: A case study in the National Park Forest Dadia-Lefkimi-Soufli, Greece. *Applied Sciences*, *12*(6), 2938.

Marjani, M., Mahdianpari, M., & Mohammadimanesh, F. (2024). CNN-BiLSTM: A novel deep learning model for near-real-time daily wildfire spread prediction. *Remote Sensing*, *16*(8), 1467.

Marsha, A. L., & Larkin, N. K. (2022). Evaluating satellite fire detection products and an ensemble approach for estimating burned area in the United States. *Fire*, *5*(5), 147.

Masrur, A., Yu, M., & Taylor, A. (2024). Capturing and interpreting wildfire spread dynamics: attention-based spatiotemporal models using ConvLSTM networks. *Ecological Informatics*, *82*, 102760.

Mehra, N., & Gupta, S. (2015). Maximal margin multi-classifier based on SVM hyperparameter tuning. 2015 International Conference on Computer, Communication and Control (IC4),

Michael, Y., Helman, D., Glickman, O., Gabay, D., Brenner, S., & Lensky, I. M. (2021). Forecasting fire risk with machine learning and dynamic information derived from satellite vegetation index time-series. *Science of The Total Environment*, *764*, 142844.

Mirones, Ó., Baño‐Medina, J., Brands, S., & Bedia, J. (2025). Toward spatio‐temporally consistent multi‐site fire danger downscaling with explainable deep learning. *Journal of Geophysical Research: Machine Learning and Computation*, *2*(1), e2024JH000331.

Mittal, M., Al-Jawahry, H. M., Varshney, N., Kumar, S. P., Michaelson, J., & Reddy, R. A. (2024). Improving Support Vector Machine Performance with Advanced Kernel Methods. 2024 7th International Conference on Contemporary Computing and Informatics (IC3I),

Mohammed, O. A., Vafaei, S., Kurdalivand, M. M., Rasooli, S., Yao, C., & Hu, T. (2022). A comparative study of forest fire mapping using GIS-based data mining approaches in Western Iran. *Sustainability*, *14*(20), 13625.

Moreno Ruiz, J. A., García Lázaro, J. R., del Águila Cano, I., & Leal, P. H. (2014). Burned area mapping in the North American boreal forest using terra-MODIS LTDR (2001–2011): A comparison with the MCD45A1, MCD64A1 and BA GEOLAND-2 products. *Remote Sensing*, *6*(1), 815-840.

Mythili, R., Abinav, K., Singh, S. K., & Krishna, S. S. (2023). Estimation of wildfire conditions via perimeter and surface area optimization using convolutional neural network. International Conference on Micro-Electronics and Telecommunication Engineering,

Nikolaychuk, O., Pestova, J., & Yurin, A. (2024). Wildfire susceptibility mapping in baikal natural territory using random forest. *Forests*, *15*(1), 170.

Novo, A., Fariñas-Álvarez, N., Martínez-Sánchez, J., González-Jorge, H., Fernández-Alonso, J. M., & Lorenzo, H. (2020). Mapping forest fire risk—a case study in Galicia (Spain). *Remote Sensing*, *12*(22), 3705.

Okut, H. (2021). Deep learning for subtyping and prediction of diseases: Long-short term memory. In *Deep learning applications*. IntechOpen.

Parajuli, A., Gautam, A. P., Sharma, S. P., Bhujel, K. B., Sharma, G., Thapa, P. B., Bist, B. S., & Poudel, S. (2020). Forest fire risk mapping using GIS and remote sensing in two major landscapes of Nepal. *Geomatics, Natural Hazards and Risk*, *11*(1), 2569-2586.

Peng, Y., Su, H., Sun, M., & Li, M. (2024). Reconstructing historical forest fire risk in the non-satellite era using the improved forest fire danger index and long short-term memory deep learning-a case study in Sichuan Province, southwestern China. *Forest Ecosystems*, *11*, 100170.

Pereira, A. A., Pereira, J. M., Libonati, R., Oom, D., Setzer, A. W., Morelli, F., Machado-Silva, F., & De Carvalho, L. M. T. (2017). Burned area mapping in the Brazilian Savanna using a one-class support vector machine trained by active fires. *Remote Sensing*, *9*(11), 1161.

Pérez-Sánchez, J., Jimeno-Sáez, P., Senent-Aparicio, J., Díaz-Palmero, J. M., & Cabezas-Cerezo, J. d. D. (2019). Evolution of burned area in forest fires under climate change conditions in southern Spain using ANN. *Applied Sciences*, *9*(19), 4155.

Pham, B. T., Jaafari, A., Avand, M., Al-Ansari, N., Dinh Du, T., Yen, H. P. H., Phong, T. V., Nguyen, D. H., Le, H. V., & Mafi-Gholami, D. (2020). Performance evaluation of machine learning methods for forest fire modeling and prediction. *Symmetry*, *12*(6), 1022.

Pham, K., Ward, D., Rubio, S., Shin, D., Zlotikman, L., Ramirez, S., Poplawski, T., & Jiang, X. (2022). California wildfire prediction using machine learning. 2022 21st ieee international conference on machine learning and applications (icmla),

Pourghasemi, H. R., Gayen, A., Edalat, M., Zarafshar, M., & Tiefenbacher, J. P. (2020). Is multi-hazard mapping effective in assessing natural hazards and integrated watershed management? *Geoscience Frontiers*, *11*(4), 1203-1217.

Pourmohamad, Y., Abatzoglou, J. T., Fleishman, E., Belval, E., Short, K. C., Williamson, M., Perlmutter, M., Seydi, S. T., & Sadegh, M. (2026). Predictive understanding of Wildfire ignitions across the western United States. *Earth's Future*, *14*(1), e2025EF006935.

Ravikumar, A., & Sriraman, H. (2024). A Deep Understanding of Long Short-Term Memory for Solving Vanishing Error Problem: LSTM-VGP. In *Machine Learning Algorithms Using Scikit and TensorFlow Environments* (pp. 74-90). IGI Global Scientific Publishing.

Santos, L., Lopes, V., & Baptista, C. (2022). MDIR monthly ignition risk maps, an integrated open-source strategy for wildfire prevention. *Forests*, *13*(3), 408.

Sapkota, S., Joshi, K. P., Kuikel, S., Kuinkel, D., Bhandari, B., Wu, Y., Bing, H., Marahatta, S., Aryal, D., & Wang, S. S. (2025). Advancing wildfire prediction in Nepal using machine learning algorithms. *Environmental Research Communications*, *7*(5), 055003.

Sawetsuthipan, T., & Wongchaisuwat, P. (2023). Forecasting Burned Areas of Wildfires: A Case Study of Mae Hong Son Province in Thailand. 2023 4th International Conference on Computers and Artificial Intelligence Technology (CAIT),

Shafapourtehrany, M. (2023). Geospatial wildfire risk assessment from social, infrastructural and environmental perspectives: A case study in Queensland Australia. *Fire*, *6*(1), 22.

Shi, C., & Zhang, F. (2023). A forest fire susceptibility modeling approach based on integration machine learning algorithm. *Forests*, *14*(7), 1506.

Shimabukuro, Y. E., de Oliveira, G., Pereira, G., Arai, E., Cardozo, F., Dutra, A. C., & Mataveli, G. (2023). Assessment of burned areas during the pantanal fire crisis in 2020 using Sentinel-2 images. *Fire*, *6*(7), 277.

Sivrikaya, F., & Demirel, D. (2025). Fire risk mapping using machine learning method and remote sensing in the Mediterranean region. *Advances in Space Research*, *75*(7), 5402-5419.

Sivrikaya, F., Günlü, A., Küçük, Ö., & Ürker, O. (2024). Forest fire risk mapping with Landsat 8 OLI images: Evaluation of the potential use of vegetation indices. *Ecological Informatics*, *79*, 102461.

Tan, C., & Feng, Z. (2023). Mapping forest fire risk zones using machine learning algorithms in Hunan province, China. *Sustainability*, *15*(7), 6292.

Thakkar, R., Abhyankar, V., Reddy, P. D., & Prakash, S. (2022). Environmental fire hazard detection and prediction using random forest algorithm. 2022 International conference for advancement in technology (ICONAT),

Vanderhoof, M. K., Menick, C. E., Picotte, J. J., Robertson, K. M., Nowell, H. K., Matechik, C., & Hawbaker, T. J. (2025). Modelling and mapping burn severity of prescribed and wildfires across the southeastern United States (2000–2022). *International Journal of Wildland Fire*, *34*(1).

Wang, C., Liu, H., Xu, Y., & Zhang, F. (2025). A forest fire prediction framework based on multiple machine learning models. *Forests*, *16*(2), 329.

Xu, Y., Guo, H., Liu, J., Sun, R., & Li, X. (2023). Wildfire risks under a changing climate: Synthesized assessments of wildfire risks over southwestern China. *Frontiers in Environmental Science*, *11*, 1137372.

Yu, Q., Zhao, Y., Yin, Z., & Xu, Z. (2024). Wildfire Susceptibility Prediction Based on a CA-Based CCNN with Active Learning Optimization. *Fire*, *7*(6), 201.

Zhang, F., Zhao, P., Xu, S., Wu, Y., Yang, X., & Zhang, Y. (2020). Integrating multiple factors to optimize watchtower deployment for wildfire detection. *Science of The Total Environment*, *737*, 139561.

Zhang, H., Zheng, Z., & Wen, G. (2022). Wildfire monitring based on LSTM and deep learning. IGARSS 2022-2022 IEEE International Geoscience and Remote Sensing Symposium,

Zhao, P., Zhang, F., Lin, H., & Xu, S. (2021). GIS-Based Forest Fire Risk Model: A Case Study in Laoshan National Forest Park, Nanjing. *Remote Sensing*, *13*(18), 3704.

Ziccardi, L. G., Thiersch, C. R., Yanai, A. M., Fearnside, P. M., & Ferreira-Filho, P. J. (2020). Forest fire risk indices and zoning of hazardous areas in Sorocaba, São Paulo state, Brazil. *Journal of forestry research*, *31*(2), 581-590.
